# Allelic sequence variation in the *Sub1A, Sub1B* and *Sub1C* genes among diverse rice cultivars and its association with submergence tolerance

**DOI:** 10.1038/s41598-020-65588-8

**Published:** 2020-05-25

**Authors:** Anuradha Singh, Yashi Singh, Ajay K. Mahato, Pawan K. Jayaswal, Sangeeta Singh, Renu Singh, Neera Yadav, A. K. Singh, P. K. Singh, Rakesh Singh, Rajesh Kumar, Endang M. Septiningsih, H. S. Balyan, Nagendra K. Singh, Vandna Rai

**Affiliations:** 1 ICAR-National Institute for Plant Biotechnology, Pusa Campus, New Delhi, India; 2 International Rice Research Institute, DAPO 7777, Metro Manila, Philippines; 3 Department of Genetics and Plant Breeding, Chaudhary Charan Singh University, Meerut, India; 4 Department of Crop Physiology, Narendra Deo University of Agriculture & Technology, Ayodhya, UP India; 5 Department of Genetics and Plant Breeding, Banaras Hindu University, Varanasi, India; 6 ICAR-National Bureau of Plant Genetic Resources, Pusa Campus, New Delhi, India; 7 Department of Genetics and Plant Breeding, Dr. Rajendra Prasad Central Agricultural University, Samastipur, Bihar India; 8 Present Address: Department of Soil and Crop Sciences, Texas A & M University, TX, 77843 USA

**Keywords:** Plant sciences, Plant sciences, Plant signalling, Plant signalling, Plant stress responses

## Abstract

Erratic rainfall leading to flash flooding causes huge yield losses in lowland rice. The traditional varieties and landraces of rice possess variable levels of tolerance to submergence stress, but gene discovery and utilization of these resources has been limited to the *Sub1A-1* allele from variety FR13A. Therefore, we analysed the allelic sequence variation in three *Sub1* genes in a panel of 179 rice genotypes and its association with submergence tolerance. Population structure and diversity analysis based on a 36-plex genome wide genic-SNP assay grouped these genotypes into two major categories representing Indica and Japonica cultivar groups with further sub-groupings into Indica, Aus, Deepwater and Aromatic-Japonica cultivars. Targetted re-sequencing of the *Sub1A*, *Sub1B* and *Sub1C* genes identfied 7, 7 and 38 SNPs making 8, 9 and 67 SNP haplotypes, respectively. Haplotype networks and phylogenic analysis revealed evolution of *Sub1B* and *Sub1A* genes by tandem duplication and divergence of the ancestral *Sub1C* gene in that order. The alleles of *Sub1* genes in tolerant reference variety FR13A seem to have evolved most recently. However, no consistent association could be found between the *Sub1* allelic variation and submergence tolerance probably due to low minor allele frequencies and presence of exceptions to the known *Sub1A-1* association in the genotype panel. We identified 18 cultivars with non-*Sub1A-1* source of submergence tolerance which after further mapping and validation in bi-parental populations will be useful for development of superior flood tolerant rice cultivars.

## Introduction

Rice is the most important staple food crop for more than half of the global population and accounts for approximately 20% of the daily food calorie intake^1^. Indian sub-continent and South-East Asia are the most important centers of diversity for rice (*Oryza sativa* L.), where rice is widely cultivated on the flood plains of the major river basins and deltas. Rice cultivation in these areas is not only crucial for the world food security but is also closely associated with the tradition, culture and customs of the people^2,3^. It is a semi-aquatic plant that can be grown in a wide range of environments varying from dry rain-fed upland to deep-water lowland rice ecosystems. Submergence at vegetative stage is quite often followed by reproductive stage drought in the rain-fed lowland river plains of India and South-East Asia. The majority of rice growing areas in this region suffers from abiotic stresses such as drought, flooding and salinity^4^. Worldwide, 15 million hectares of rice suffers from flooding stress of one kind or other causing huge economic losses^5^. Different kinds of flooding include: (i) flooding during seed germination, (ii) complete submergence for 1–2 weeks due to flash flooding at seedling or tillering stage, (iii) stagnant water flooding of 25–50 cm in the areas with poor drainage, near the banks of overflowing rivers, (iv) saline water flooding in the coastal and tidal areas, and (v) deep water flooding lasting for a prolonged period of more than a month with water levels ranging from a 50 cm to several meters^5,6^. Traditional varieties and landraces of rice are available for cultivation in each of these situations. These are low yielding but can be useful source of genes for breeding high yielding stress tolerant rice cultivars. Several QTLs and genes for different kinds of flooding stress have been identified from these germplasm^7–16^. During 1970s hundreds of landraces of rice collected from the flood-prone areas of the world were screened for flash flood submergence tolerance at IRRI. Consequently, FR13A, FR43B, Kurkarruppan, Goda Heenati, Thuvalu, etc. were recognized as flash flooding tolerant.

Aus cultivar FR13A was found to be one of the best sources for submergence tolerance. Genetic studies using a population derived from FR13A identified a major QTL Submergence 1 (*SUB1*) on the chromosome 9^17^. This was followed by map-based cloning of three ethylene response factor-like genes, *Sub1A*, *Sub1B*, and *Sub1C*, and demonstration of the role of *Sub1A* in submergence tolerance by genetic transformation of a sensitive variety Liaogeng^18,19^. Analysis of 21 rice accessions showed that all of them possessed *Sub1B* and *Sub1C* genes, but only a sub-set of 12 cultivars possessed the *Sub1A* gene, responsible for the submergence tolerance in FR13A^19^. The *Sub1A* positive varieties possessed one of the two alleles, *Sub1A-1* or *Sub1A-2*, which were associated with the tolerant or intolerant phenotype, respectively^19^. At the protein sequence level, the only difference is that *Sub1A-1* allele codes for amino acid Ser_186_, whereas *Sub1A-2* codes for a Pro_186_ at this position, the remaining protein is identical. Phylogenetic analysis of the *Sub1* genes from wild rice accessions has revealed that *Sub1A* arose from the duplication of *Sub1B* gene^20^. However, there are limited studies on sequence variation of the *Sub1B* and *Sub1C* genes and *Sub1A*-independent mechanisms of submergence tolerance in flood-tolerant rice germplasm. Earlier, a set of 109 rice genotypes including A and C genome wild rice species were analyzed for stem elongation index and percent survival after 14 days of submergence followed by seven days of recovery and identified *Sub1A*-dependent and *Sub1A*-independent mechanisms of survival^21^. Further, a set of 160 rice varieties representing deep water, flood and tidal prone areas were analyzed for haplotype variation in the *Sub1A* and *Sub1C* genes using CAPS markers to identify three and four haplotypes, respectively^22^. Recently, Zhao *et al*.^23^ developed a pan-genome of rice based on deep coverage re-sequencing of 66 diverse rice genotypes, representing different rice cultivar groups and 14 *Oryza rufipogon* accessions, but they analyzed the presence of *Sub1A* gene only and not *Sub1B* and *Sub1C* genes in the set.

Submergence tolerant rice cultivars have been developed in the background of several popular mega varieties of rice and deployed for commercial cultivation across South and South-East Asia using marker-assisted backcross breeding to transfer 0.8 to 7.8 Mbp of the donor chromosome 9 segment around the *Sub1* locus with varying degree of success^24,25^. However, there is need to find novel genes and alleles for tolerance to submergence and other kinds of flooding stresses including anaerobic germination, deep water and prolonged water stagnation. Collection and characterization of rice landraces and traditional varieties adapted to flood-prone environments is important for the identification of donors with new genes and alleles. Present study aimed to analyze rice accessions from flood-prone areas of South and South East Asia, obtained through Genetic Resource Center (GRC), International Rice Research Institute (IRRI), Philippines and National Bureau of Plant Genetic Resources (NBPGR), New Delhi, India, along with additional genotypes obtained directly from rice breeders and farmers for population structure and sequence variation in the *Sub1* genes. A set of 179 diverse rice accessions was assembled and screened for vegetative stage submergence tolerance at multiple locations to identify new donors. Further, the three *Sub1* genes were amplified and sequenced to obtain SNP haplotype information and its possible association with the performance of the varieties under submergence stress.

## Methods

### Plant material

A set of 179 diverse rice genotypes was used (Supplementary Table S1). The seeds were obtained from GRC, International Rice Research Institute (IRRI), Las Banos, Philippines; ICAR-National Bureau of Plant Genetic Resources (NBPGR), New Delhi; Narendra Dev University of Agriculture and Technology (NDUAT), Kumarganj, Ayodhya; ICAR-Indian Agricultural Research Institute (IARI), New Delhi; ICAR-National Rice Research Institute (NRRI), Cuttack; and ICAR- National Institute for Plant Biotechnology (NIPB), New Delhi, including some varieties collected directly from the farmer’s field and maintained by ICAR-NIPB.

### Genotyping with 36-plex SNP assay and analysis of population structure and diversity

DNA extraction was performed using QIAGEN DNeasy Plant Mini Kit (Hilden, Germany) from de-husked rice seeds as per manufacturer’s protocol. Sequenom MassARRAY system was used for the SNP genotyping (Sequenom Inc., San Diego, CA, USA; www. sequenom.com). The Sequenom MassARRAY multiplex assay, based on iPLEX gold chemistry, was designed for 36 SNPs, representing conserved single-copy rice genes^26^, taking three genes (two near the telomeres and one near the centromere) from each of the 12 rice chromosomes^27^. The 36-plex premixed assays were manufactured and validated by Sequenom Corporation (San Diego). The 30-mer pre-amplification primers and variable length genotyping primers were designed by AssayDesign 3.1 software. Sequenom MassARRAY Typer v3.4 Software was employed for SNP visualization and allele calling. The STRUCTURE v2.3.1 software was used to infer ancestral lineages and clusters of similar genotypes^28^. The clustering of genotypes were run for a range of K values from 1 to 9 with the admixture model and correlated allele frequency, replicated three times for each K value. A burn-in time span of 25,000 steps followed by 100,000 Monte Carlo Markov Chain replicates was implemented for each run. The ΔK values were plotted with Ln(PD) derived for each K value to generate Evanno plot^29^. Structure harvester software was used to calculate the optimum population structure (http://taylor0.biology.ucla.edu). The proportion of genome of an individual that belongs to each inferred population (admixture) was also estimated. A dendrogram of the 179 rice cultivars was constructed based on their 36 SNP scores. Concatenated SNP markers were aligned using ClustalW programme inbuilt in the BioEdit tool^30^. The tree was constructed using neighbor-joining method and phylogeny of the tree was tested using interior-branch test approach with 10,000 bootstrap replications. The genetic distance between sequences were analyzed using proportional (p) distances algorithm inbuilt in the MEGAX software^31^. The developed tree was visualized in figtree v1.4.3 programme (http://tree.bio.ed.ac.uk/software/figtree/)^32^.

### Targeted re-sequencing of the *Sub1* genes using Ion Torrent PGM

Reference sequence of the three *Sub1* genes was downloaded from the NCBI GenBank (Sequence Id. for *Sub1A, Sub1B*, and *Sub1C* were DQ011598.1:3885-6447, DQ453964.1:2472-4557 and DQ453965.1:14767-16504, respectively) and PCR primers were designed using the Primer3 software (Table 1). Full-length genes were amplified and sequenced using Ion Torrent PGM system after random fragmentation, followed by assembly of the full gene sequence by mapping on the reference sequence using Ion Torrent PGM software. The PCR parameters for *Sub1A* gene amplification was initial denaturation at 95 °C for 5 min followed by 35 cycles of denaturation at 98 °C for 10 s, annealing at 68.5 °C for 10 s, and extension at 72 °C for 2.30 min, and then a final extension at 72 °C for 10 min. For *Sub1B* and *Sub1C* genes, initial denaturation at 95 °C for 5 min was followed by 35 cycles of denaturation at 98 °C for 10 s, annealing at 65.5 °C for 10 s, and extension at 72 °C for 2 min, and a final extension at 72 °C for 10 min. The PCR amplified products were checked by electrophoresis in 0.8% agarose gel. A flow diagram for the pooled amplicon sequencing of bar coded samples is shown in Supplementary Fig. S1. Pooled PCR products (510 ng) were end-repaired, and Ion Torrent adapters P1 and A were ligated using DNA ligase. Following AMPure bead (Beckman Coulter, Brea, CA, USA) purification, adapter-ligated products were nick-translated and PCR-amplified for 10 cycles. The resulting library was purified using AMPure beads (Beckman Coulter) and the concentration and size determined by Fluorometry and E–gel, respectively. The sample was then prepared for sequencing using the Ion Sequencing Kit protocol. The pooled sample was loaded on an Ion 316 chip and sequenced for 65 cycles.

**Table 1:** Primers used for PCR amplification of full-length *Sub1A, Sub1B* and *Sub1C* genes.

Gene	Primer	Sequence (5′ to 3′)	Product size (bp)
*Sub1A*	Forward Reverse	CGATCATACAGGCAGCACAGAGTTA GGGTTACACGACCCAACGTACAC	2637
*Sub1B*	Forward Reverse	TCCTTATGTAGCATTGGGAAGTCTG CATCAATTGAAGTCCAAGCTAGGTAAC	2075
*Sub1C*	Forward Reverse	CCATTGCAATCCTTGTTAAATTCT TTTCAATGAACAAAATGGCCTTC	1724

### Identification of SNPs, haplotypes and haplotype networks of the *Sub1* genes

Data from the Ion Torrent PGM sequencing runs were processed using the Ion Torrent platform-specific software to generate sequence reads, trim adapter sequences and SNP calling by aligning with the reference sequence of the *Sub1* genes. The consensus sequence for each variety was obtained using an in-house script; the heterozygous base positions were represented by actual international codes for two nucleotide combinations. DnaSP software was used for combined analysis of intervarietal comparisons. Sliding window analysis was performed to examine nucleotide polymorphism for the *Sub1A*, *Sub1B* and *Sub1C* genes in all the varieties with sequence information (DnaSP software version 5.10). Gene-specific PCR marker AEX1 was used to crosscheck the presence of *Sub1A-1* allele^25^. The haplotype information based on high-quality SNPs in the three *Sub1* genes was compiled manually for each rice genotype. Haplotype networks were constructed for analysis of genealogical relationship using Network software^33^ and haplotype diversity was calculated using DnaSP v5.10^34^.

### Analysis of the *Sub1* genes in 66 re-sequenced genomes

Draft genome assemblies of 66 rice genotypes, including 52 cultivars and 14 *O. rufipogon* wild rice accessions, were downloaded from the RicePanGenome database (http://db.ncgr.ac.cn/RicePanGenome/) and a local database was created. The reference sequence of *Sub1A*, *Sub1B* and *Sub1C* genes was used for BLASTN search^35^ against a locally created BLAST database of 67 genomes (including Nipponbare reference genome) using the parameters: -word size = 5, -perc identity = 80, -qcovs = 80. The results were tabulated and the hits were filtered at a bit score of ≥1000, nucleotide sequences of all the filtered hits were extracted and fasta files were created. Multiple sequence alignment of the fasta files was performed using Clustalw^36^, using default parameters and the SNP/InDel information was tabulated manually.

### Phylogenetic analysis of the rice *Sub1* genes

We searched for the presence of rice *Sub1A*, *Sub1B* and *Sub1C* gene homologs in the non-redundant “Green plant” database downloaded from NCBI (https://www.ncbi.nlm.nih.gov/). For similarity search a standalone NCBI-BLASTX programme was used with default search parameters. Further, we also searched for the copy numbers of rice *Sub1C* gene homologs in the reference genomes of 20 different monocotyledonous, dicotyledonous, moss and algal species with BLASTN programme using search parameters optimized earlier for such analysis^37^. Full-length sequence of the *Sub1A*, *Sub1B* and *Sub1C* genes present in each of the 13 rice varieties and *Sub1C* gene homolog from *Sorghum bicolor*, used as an outgroup, were aligned using Clustalw inbuilt in the BioEdit software^30^. The maximum likelihood based phylogenetic tree of the three genes was developed using RAxML v8.2.12 software^38^ and statistical reliability for each node support was estimated from 1000 replicates of non-parametric bootstrap with HKY85 model. The *Sub1C* gene homolog in *Sorghum bicolor* was used as outgroup for rooting the tree. The tree was visualized in figtree v. 1.4.3^32^. The divergence times of *Sub1A*, *Sub1B* and *Sub1C* genes were estimated using the formula, T_2_ = (K_AB_T1/K_AX_ + K_BX_), where T_2_ = divergence time of *Sub1A* and *Sub1B*, T_1_ = divergence time of sorghum and rice, taken as 50–70 Mya^39–41^, K_AB_, K_AX,_ and K_BX_ are the synonymous substitution values between *Sub1A and Sub1B* of rice, *Sub1A* of rice and *Sub1C* of sorghum, and *Sub1B* of rice and *Sub1C* of sorghum, respectively^42^. The synonymous substitution values for each pair of genes were estimated using DnaSP programme^34,43^.

### Phenotypic evaluation of vegetative stage submergence tolerance

Screening for survival under submergence was carried out at International Rice Research Institute (IRRI), Las Banos, Philippines; Narendra Deo University of Agriculture and Technology (NDUAT), Ayodhya, India; Banaras Hindu University (BHU), Varanasi, India; and Dr. Rajendra Prasad Central Agricultural University (RPCAU), Samastipur, India. At IRRI in 2012, the vegetative stage submergence screening was performed using an established protocol^25^. Seeds were sown in plastic trays filled with soil, each tray accommodating 10 entries with 30 plants each in Alpha Plus design to enable randomization of all entries in replication. After 14 days from sowing, the number of seedlings was recorded and the trays containing seedlings were transferred to a concrete submergence tank filled up to 1.5 m height with tap water. Once the susceptible IR42 plants showed severe stress symptoms (about 15 days of submergence) the plants were de-submerged and left for regeneration. Plant survival was recorded 21 days after de-submergence. At NDUAT, in *Kharif* season of 2012, experiment was laid out in augmented block design with two replications in concrete submergence pond of 20 × 10 × 1.2 m dimensions. Each block had 14 test entries plus two tolerant (Samba Mahsuri-Sub1 and FR13A) and one susceptible (Swarna) check. One-month old seedlings were transplanted in the ponds with row lengths of 2 m, and after establishment for two weeks the plants were submerged for 18 days. In one set as the plants grew under submerged conditions the canopies of highly elongating plants were cut to prevent breathing from the air. Fifteen days after de-submergence the number of surviving plants were recorded for both with and without canopy. The submergence screening was repeated at NDUAT in 2013 but in a lowland field with natural flooding. Experimental design and checks were similar to 2012 but this time with direct seeded rice with spacing of 20 × 10 cm. One month after sowing the fields were naturally flooded under 45–50 cm depths of water for 15 days. Then flood receded and plant survival was recorded 15 days after de-submergence. At BHU, in *Kharif* season of 2012, rice seeds were planted directly in a muddy pond and after 21 days of growth submergence was imposed for 14 days and then water was pumped out and plant survival was recorded one week after de-submergence. At RPCAU, in *Kharif* season of 2012, one-month old direct seeded rice plants in augmented design with FR13A and Samba Mahsuri-Sub1 as tolerant checks and IR42 as sensitive check replicated in every block were flooded with 1 m depth of water in a submergence tank. Percent survival of seedlings was recorded after 12 days of submergence followed by 15 days of recovery on de-submergence.

### Association between *Sub1* SNP haplotypes and submergence tolerance

The phenotypic performance as percent survival after submergence treatment, and SNP data for each of the three *Sub1* genes along with its position in the sequence was fed into TASSEL v3 (2011) software^44^ to find association between *Sub1* SNPs and submergence tolerance. A General Linear Model (GLM) analysis was employed, as MLM model did not produce any significant association, and Manhattan plots were prepared. In addition, the relationship of SNP haplotypes of *Sub1A*, *Sub1B* and *Sub1C* genes, Structure sub-populations and percent survival after submergence of the rice cultivars was also visualized in 3D scatter plots drawn using NCSS v19.0.3 software (https://www.ncss.com, date of access 28 Dec 2019) on IRRI 2012 phenotypic data. All the SNP haplotypes of *Sub1A* and *Sub1B* genes were used in the 3D scatter plot, but for *Sub1C* gene only those haplotypes with cultivar frequency of ≥3 were taken for clear visualization.

## Results

### Genetic diversity and population structure of 179 rice cultivars

**Figure 1:**
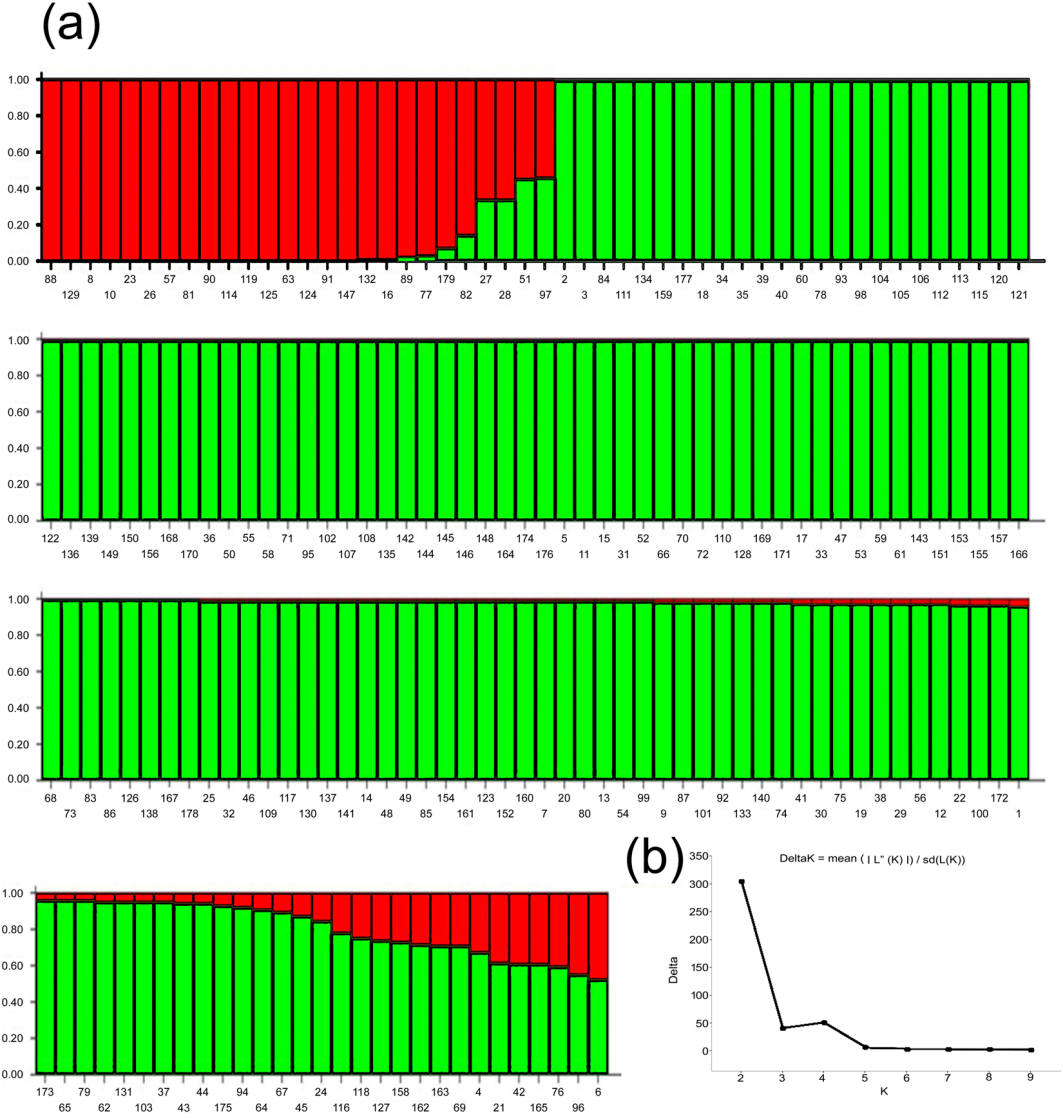
Population structure of 179 rice cultivars based 36 genome wide unlinked SNP markers. **(a)** Two sub-populations corresponding to ‘Aromatic-Japonica’ (red) and ‘Indica’ (green) cultivar groups. Admixture types in the two sub-populations (with part red and green shades) were ‘Aus’ cultivars that formed a separate group at K = 4, **(b)** Delta *K* vs *K* Evanno plot showing peak delta at *K* = 2.

Genetic variability of the 179 rice genotypes was assessed using a genome wide 36-plex SNP assay. SNP marker 12-1794 showed the highest major allele frequency of 0.9665, while 07-4304 showed the lowest major allele frequency of 0.4804 (Table 2). Polymorphism information content (PIC) values of the SNP markers ranged from 0.0636 for 12-1794 to 0.4436 for 10-1192-7_C_178 with an average of 0.2788 (Table 2). Analysis of population structure of the rice genotypes revealed two major sub-populations based on the results of Structure Harvester Evanno Plot between delta K and K (Fig. 1a,b). The optimum number of sub-populations (K) was determined after conducting multiple independent runs with K values ranging from 2 to 10. Most of the genotypes clustering in the two groups possessed Fst values of ≥0.80 indicating more than 80 percent inferred ancestry. Four varieties of sub-population 1, namely Aus 287, Ausboro, Jalmagna and Kasalath, and 14 varieties of sub-population 2, namely ARC 12172, Bagaikra, Dihawa, Dubgelong, Dular, FR13A, Kajrahawa, Kalonchi, Kharsu 80A, Khaiyan, Meghi, NCS 348, Suraha and Tundahia with Fst values of <0.80 possessed substantial admixture from the other sub-population. Sub-population 1 comprised 26 genotypes, mostly belonging to Japonica and Aromatic cultivar groups along with some locally adapted cultivars of unknown ancestry such as Badkodi, Baddhana, Bagagoha, Jokhru and Meghraj. Sub-population 2 comprised 151 genotypes belonging to Aus and Indica cultivar groups along with locally adapted Deep-water cultivars specific to flood-prone areas, e.g. Dudhaladu, Kalabunde, Kariyawa, Sugapankh, Suraha and Tundahia (Fig. 1a, Supplementary Tables S1 and S2). The bar diagram and Fst values clearly show that four genotypes in sub-population 1 (red shade), have more than 20% admixture from sub-population 2, whereas 14 genotypes in sub-population 2 (green shade) have more than 20% admixture from sub-population I. Since most of the molecular marker-based studies have reported five groups of rice cultivars, namely Aromatic, Aus, Indica, Temperate Japonica and Tropical Japonica, we further analyzed the population structures at K values of 4 and 5. Interestingly, the combined Aromatic-Japonica cluster in Fig. 1a was not separated even at K value of 5; therefore we restricted our analysis to K value of 4. This separated the Deep-water (sub-population 1), Indica (sub-population 2), Aus (Sub-population 3) and Aromatic-Japonica cultivars together in sub-population 4 (Supplementary Fig. S2, Table S3). Significantly, 17 of the 18 genotypes showing admixture of ancestry between Indica and Japonica cultivar groups at K = 2 (Fig. 1, lighter shades in Table S2), exclusively made the sub-population 3 representing Aus cultivars at K = 4 (orange shade in Table S3), only cultivar Jalmagna with Fst value 0.663 still stayed in the Aromatic-Japonica group. The new sub-population 1 (blue shade in Table S3), representing mostly flood tolerant deep-water rice cultivars of geographically diverse origin in India, Bangladesh, Guinea, Indonesia, Sierra Leone, Sri Lanka and Thailand was separated from the large Indica sub-population at K = 2.

**Table 2:** Major allele frequency and polymorphism information content (PIC) of 36 SNPs used in population structure and genetic diversity analysis of 179 rice cultivars.

Marker Id.	Major Allele Frequency	PIC
01-3916-1_C_156	0.7095	0.3399
01-608-4_C_375	0.5978	0.4309
01-6351-1_C_202	0.8324	0.2484
02-267	0.6313	0.4164
02-3029-1_C_474	0.7207	0.3597
02-4333-1_C_293	0.7430	0.3259
03-1691-1_C_373	0.7039	0.3364
03-3478-1_C_206	0.6592	0.3686
03-4660-1_C_355	0.6369	0.3628
04-1801-20_C_428	0.9609	0.0723
04-19-4_C_240	0.9609	0.0745
04-3787-3_C_358	0.6927	0.4168
05-2692-1_C_109	0.8994	0.1645
05-4192-1_C_280	0.6592	0.3686
05-48-1_C_279	0.5363	0.4320
06-1256-1_C_147	0.9497	0.0909
06-1776-1_C_501	0.7989	0.3014
06-2509-1_C_497	0.8492	0.2508
07-2904-39_C_299	0.9218	0.1414
07-293-12_C_368	0.8547	0.2175
07-4304	0.4804	0.4425
08-2765-2_C_360	0.9441	0.1039
08-4218-5_C_129	0.5363	0.4187
08-847-6_C_113	0.4916	0.4216
09-209	0.8603	0.2244
09-2107-5_C_145	0.6983	0.3729
09-2716-4_C_457	0.8939	0.1773
10-1192-7_C_178	0.5531	0.4436
10-188-1	0.6927	0.4265
10-2723	0.9274	0.1324
11-1849	0.8771	0.1924
11-3935	0.9330	0.1207
11-522-1_C_214	0.6872	0.3683
12-1794	0.9665	0.0636
12-3200-2_C_389	0.8715	0.2055
12–400	0.8715	0.2023
**Mean**	**0.7668**	**0.2788**

**Figure 2:**
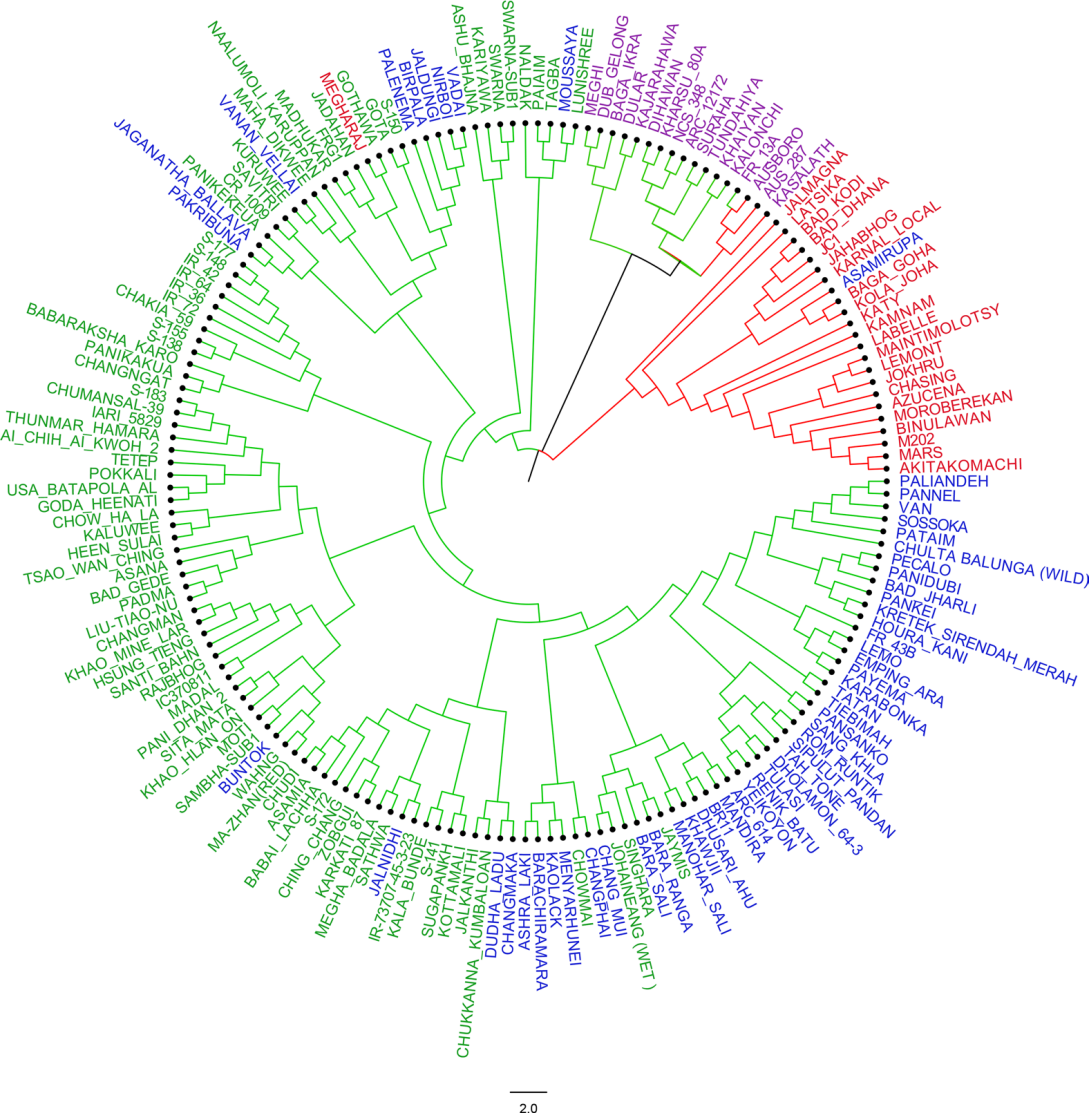
NJ diversity tree of 179 rice cultivars used for allele mining of the *Sub1* genes based on dissimilarity index of 36 genome wide unlinked SNP markers. Clade lines are colored according to groupings at K = 2, and cultivar names are colored according to groupings at K = 4. ‘Indica’ (Green), ‘Aromatic-Japonica’ (Red), ‘Aus’ (Magenta), and ‘Deep-water’ (Blue).

A NJ diversity tree of 179 rice cultivars developed based on the 36 genome-wide SNP markers formed a separate cluster of 22 Aromatic-Japonica cultivars corresponding to the Structure sub-population 4 (red fonts in Fig. 2). Only one Aromatic-Japonica cultivar Megharaj was excluded and one deep-water cultivar Asamirupa was included in this cluster. All the 17 Aus cultivars of the Structure sub-population 3 formed a separate cluster in the NJ tree without exception (purple font in Fig. 2). Most of the lowland deep-water rice cultivars of Structure sub-population 1 were also grouped in a single clade (blue font in Fig. 2). The Indica group of cultivars of Structure sub-populations 3 formed six different neighboring sub-clades (green colour in Fig. 2). The Indica and Deep-water cultivars were part of a single clade separated from Aus and Aromatic-Japonica clades at the base of the NJ tree. The red and green clade colors in the NJ tree clearly show how three cultivars from the Japonica (Ausboro, Aus 287 and Kasalath) and 14 cultivars from the Indica (Dular, FR 13 A, Kalonchi, Khaiyan etc.) sub-populations with low Fst values at K = 2 (Fig. 1, Table S2) formed the separate clade of Aus sub-population at K = 4.

### Nucleotide sequence variation in the *Sub1A*, *Sub1B* and *Sub1C* genes

**Figure 3:**
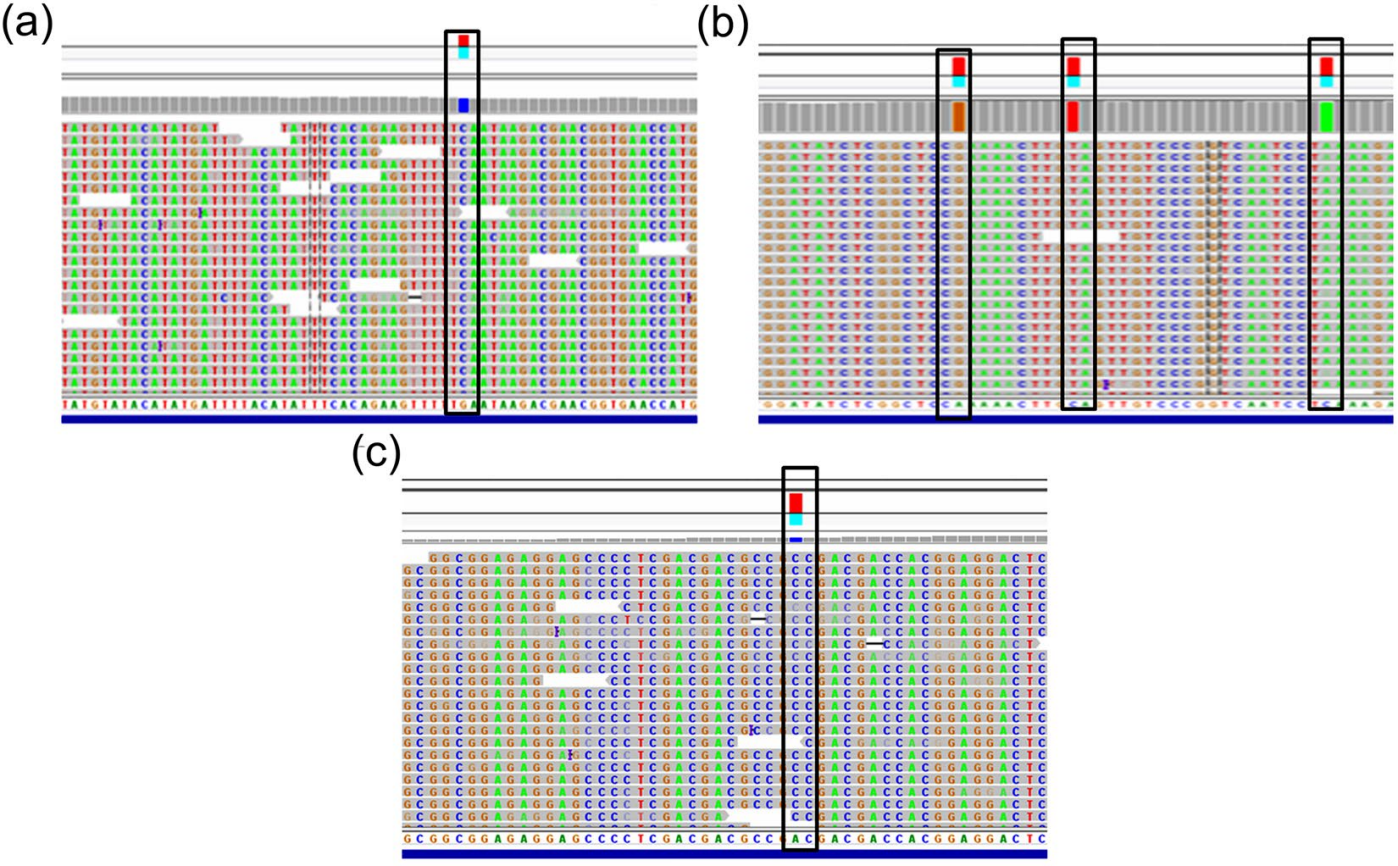
Sequence alignment and SNP calling for the *Sub1* genes in Indica cultivar ‘Madhukar’ using IonTorrent PGM software. High-quality SNPs are tagged with red and blue squares in the consensus sequence on top of the alignment panel. **(a)** *Sub1A*, **(b)** *Sub1B*, **(c)** *Sub1C*.

**Figure 4:**
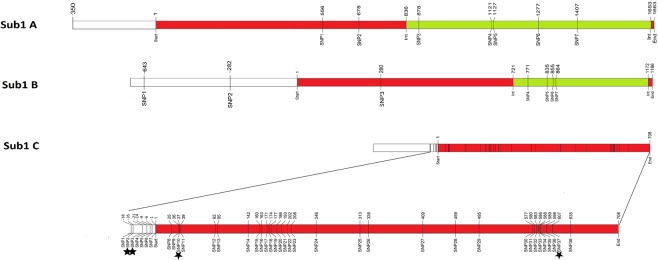
Positions of high-quality SNPs/InDels in the *Sub1* genes. Numbering of bases starts at the ATG start codon. *Sub1C* has 34 SNPs and four InDels indicated by *, two each in the 5′ upstream and exonic regions.

Three pairs of PCR primer were designed and optimized for amplification of full-length *Sub1A*, *Sub1B* and *Sub1C* genes (Table 1, Supplementary Fig. S3). As some of the rice cultivars are known to be null for *Sub1A* gene, amplification was not expected in all the genotypes for *Sub1A*, and indeed it amplified in only 96 of the 179 cultivars (Supplementary Table S4). For unknown reasons even *Sub1B* was amplified in 110 cultivars only despite repeating the experiment at least three times. This could be either due to absence of *Sub1B* gene in these genotypes, or more likely due to sequence mismatches at the 3′ end of the primers in these cultivars due to presence of SNPs/InDels. Best results were obtained with *Sub1C* where amplification was obtained in 174 of the 179 cultivars. The lack of amplification of *Sub1C* in five cultivars may also be because of sequence mismatches due to presence of mutations at the primer position or in rare case absence of the gene. The Ion Torrent PGM re-sequencing of pooled barcoded amplicons from 179 cultivars generated high-quality sequence data and polymorphism information. The assembled gene sequences have been deposited in NCBI public repository with Accession numbers: BankIt1844231: KT766732-KT766826; BankIt1856637: KT766827-KT766937; BankIt1856643: KT766938- KT767110, for the *Sub1A*, *Sub1B* and *Sub1C* genes, respectively. The SNP calls were made and exported in Excel file format using the Ion Torrent software suite by alignment with the reference BAC sequence of variety FR13A, which has all the three *Sub1* genes^19^. An example of this is shown for Indica rice cultivar Madhukar, which also has the three *Sub1* genes sequenced (Fig. 3). The SNP calls were of very high quality due to deep sequence coverage with high quality bases for each call (>10 sequence reads, each with minimum base quality score of Q20), and visual cross verification of the sequence alignment and SNP calls through the Ion PGM sequence analysis suite (Fig. 3). SNPs with low coverage or quality of sequence reads and all InDels, except four large InDels in *Sub1C* gene, were not considered for haplotype analysis. Only high quality variants, 7 SNPs each in *Sub1A*, *Sub1B* genes and 38 SNPs/InDels in *Sub1C* gene were taken for haplotype analysis. The positions of these high quality variants in the 5′ upstream promoter, intronic and exonic regions of the three genes with respect to the ATG start codon of the FR13A reference genes are shown in Fig. 4. In the protein coding exonic region, there were 2 SNPs in *Sub1A* and 1 SNP in *Sub1B* as compared to 29 SNPs and 2 InDels in *Sub1C* gene. In the 5′ promoter region there was no SNP in *Sub1A* up to −350 bp position, two SNPs in *Sub1B* at positions −643 and −282 as compared to 5 SNPs and 2 InDel in *Sub1C* at positions −38, −35 (InDel), −21 (InDel), −14, −9, −6 and −1. In the intronic region there were 5 SNPs in *Sub1A* and 4 SNPs in *Sub1B*, whereas *Sub1C* has no intron (Fig. 4).

After the initial work by Xu *et al.*^19^, there has been no targeted re-sequencing study to identify the allelic sequence variations in the rice *Sub1* genes. However, whole genome re-sequence data are available in public domain e.g. 3000 rice varieties with average sequence depth of 14 × (3 K RGP 2014)^45^. Recently, a high-depth of coverage (average 115×) sequence data on 66 rice genomes, including 52 cultivars and 14 wild rice accessions, has been analyzed for the presence of *Sub1A* gene^23^, but not for allelic sequence variation in any of the three *Sub1* genes. We analyzed these 66 genomes along with the reference Nipponbare genome and FR13A *Sub1* genes for allelic sequence variation and compared it with our targeted re-sequencing results. Detailed results for the three *Sub1* genes are presented in Supplementary Figs. S5–S7 and Table S5, but results with *Sub1A* gene is shown in Table 3. Six of the seven SNPs identified by targeted re-sequencing of *Sub1A* gene were common to the 66 genomes but one SNP at nucleotide position 878 was unique to the 179 cultivars set. Two SNPs were unique to the 66 genomes set but both of these were contributed by wild rice genotypes e.g. W0141. For the *Sub1B* gene only three of the seven SNPs identified by targeted re-sequencing were common to the 66 genomes, the remaining four SNPs were unique to the 179 cultivars set. Twenty-nine SNPs, mostly contributed by wild rice genotypes, were unique to the 66 genomes set (Supplementary Table S5). Most of the unique SNPs in the 66 genomes set were present in the 5′ upstream region of the *Sub1B* gene between −449 to −74 bp positions, where sequence coverage in the 66 genomes was poor, hence some of these could be due to sequencing errors rather than true SNPs. For the *Sub1C* gene, 28 of the 38 SNPs/InDels identified by targeted re-sequencing were common to the 66 genomes but 8 SNPs and two InDels were unique to the 179 cultivars. We found 22 SNPs unique to the 66 genomes that were spread throughout the gene but these were also contributed mostly by the wild rice genotypes (Supplementary Table S5).

**Table 3:** Comparison of SNPs in the *Sub1A* gene identified by targeted re-sequencing of 96 rice cultivars from which the gene could be amplified and sequenced, and analysis of 66 whole genome sequences of cultivated and wild rice (Zhao *et al*. 2018). SNP positions are indicated with respect to the ATG start codon.

Sr. no.	SNP position (66 Genome)	SNP position (179 cv.)	Reference base	Variant base	Common/Unique to genotype set
1.	556	556	T	C	Common
2,	678	678	A	G	Common
3.	—	878	C	G	179 cultivars
4.	1121	1121	G	C	Common
5.	1127	1127	C	G	Common
6.	1277	1277	G	A	Common
7.	1385–1386	—	CG	TG/CA	66 genomes (W0141)
8.	1407	1407	A	G	Common
9.	1567	—	T	C	66 genome (W0141)

### *Sub1* homologs in other plant species and evolution of rice *Sub1* genes

A BLASTX search in the NCBI NR database using the reference sequence of three *Sub1* genes from cultivar FR13A resulted in only 22 significant matches with species other than *Oryza sativa* at a cutoff bit score of 100. Four of these matches were with *Sub1A*, one match with *Sub1B*, and 17 matches with *Sub1C* in 12 different species, namely *Ipomoea batatas, Ipomoea nil, Lycoris longituba, Mucuna pruriens, Nicotiana tabacum, Nicotiana tomentosiformis, Oryza brachyantha, Oryza eichingeri, Oryza rhizomatis, Oryza rufipogon, Tarenaya hassleriana* and *Zostera marina* (Supplementary Table S6). All the matches with *Sub1A* and *Sub1B* genes were limited to *Oryza rufipogon* and *Oryza brachyantha*. However, *Sub1C* homologs were found in eight other non-*Oryza* species, suggesting that *Sub1C* is the most ancient of the three *Sub1* genes. Therefore, we searched for the presence of *Sub1C* homologs and its copy number in 20 completed plant genomes. Only nine of the 20 species analyzed possessed the *Sub1C* homologs and the copy number varied from one to three. Interestingly, only *Camellia sinensis* and *Glycine max* of the ten selected dicot species possessed *Sub1C* homologs. The duplicate matches in non-*Oryza* species were on different chromosomes ruling out tandem duplication (Supplementary Table S7).

**Figure 5:**
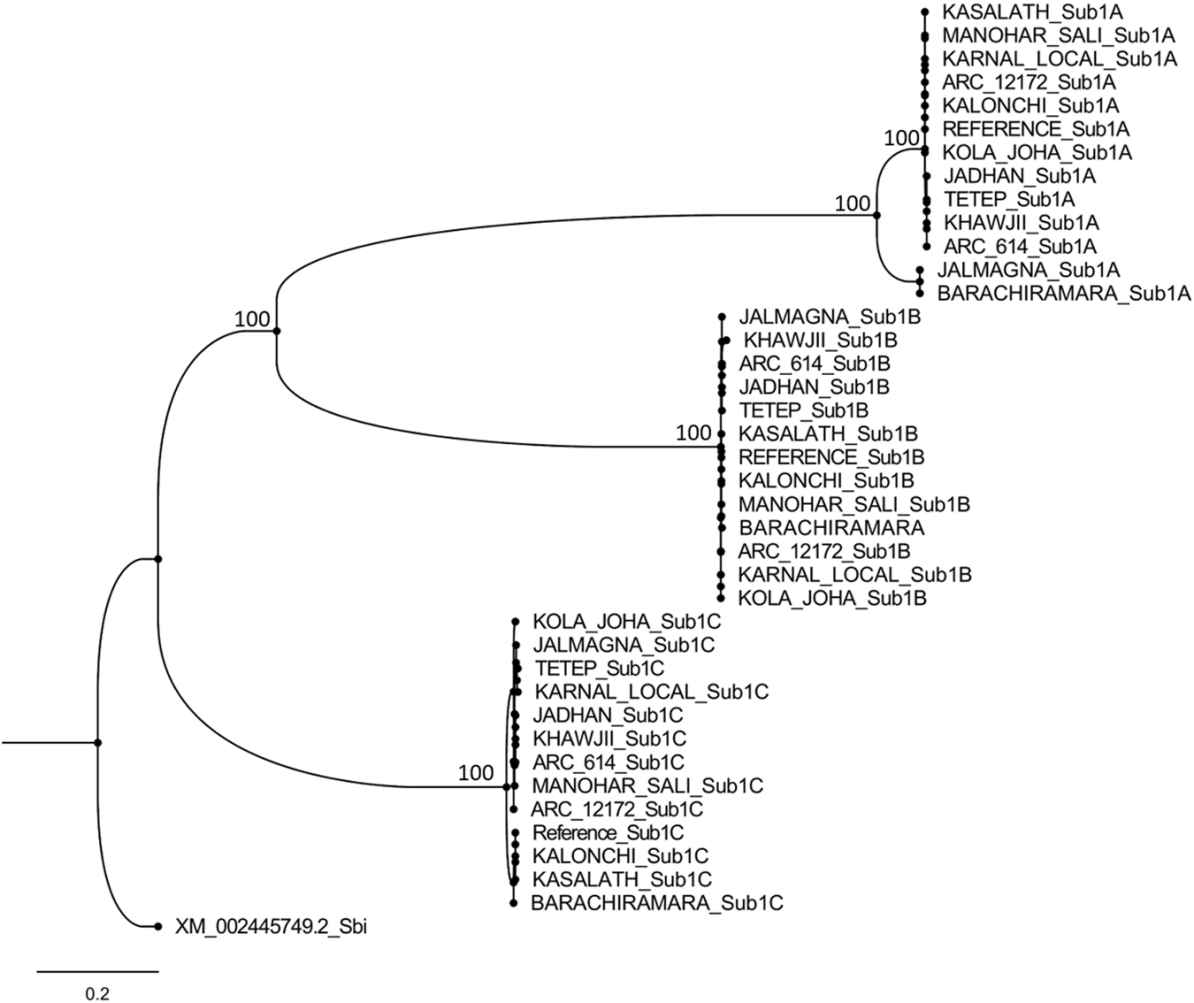
ML phylogenetic tree of *Sub1A, Sub1B* and *Sub1C* genes based on their sequence in 13 rice cultivars, and using *Sorghum bicolor* as outgroup. The tree was generated employing RAxML v8.2.12 algorithm and rooted with *Sorghum bicolor* as outgroup. Bootstrap analysis was performed with 1000 reiterations.

To study the evolution of *Sub1* genes in rice a maximum likelihood (ML) phylogenetic tree of the three genes was developed. Full-length sequence of the *Sub1A*, *Sub1B* and *Sub1C* genes present in each of the 13 rice varieties were taken along with the *Sub1C* homolog in *Sorghum bicolor*, used as an outgroup (Fig. 5). The phylogenetic tree indicated evolution of *Sub1B* and *Sub1A* genes from the ancestral *Sub1C* gene in that order by tandem duplication and divergence in the genus *Oryza* as only *Sub1C* homologs were present in the species other than genus *Oryza*. The divergence involved insertion of an intron towards the end of single exon in the *Sub1C* gene to give rise to *Sub1B*. Subsequently, the size of this intron has almost doubled from 451 bp in *Sub1B* to 817 bp in *Sub1A*. The estimated divergence time based on synonymous substitution rates showed that *Sub1A* diverged from *Sub1B* 6.44–9.02 Mya, taking monocot-dicot divergence time of 50–70 Mya based on fossil records for calibration of the tree. The estimated divergence time between *Sub1B* and *Sub1C* was 12.14–17.00 Mya.

### SNP haplotypes and haplotype networks of the *Sub1* genes

Seven high-quality SNPs identified in *Sub1A* gene by targeted re-sequencing of 96 rice cultivars made eight haplotypes designated H1 to H8, with submergence tolerant cultivar FR13A having reference haplotype H8 (Table 4). Two of the SNPs in the intronic region, namely SNP3 and SNP6 are novel that have not been reported previously. As reported earlier a threonine (T) at position 556 (SNP1) is the functional SNP coding for amino acid serine_186_ that confers submergence tolerance to FR13A, whereas the alternate base cytosine (C) at this position codes for amino acid proline_186_ resulting in submergence susceptible phenotype^19^. Since there was no new SNP in the exonic region in any of the varieties sequenced, no novel protein haplotype of the *Sub1A* gene was found. The eight SNP haplotypes resulted in two protein haplotypes, corresponding to the tolerant allele *Sub1A-1* and intolerant allele *Sub1A-2*. SNP haplotypes H3-H8 coded for protein haplotype 1, whereas SNP haplotypes H1 and H2 produced protein haplotype 2 (Supplementary Table S9). Similarly, seven high-quality SNPs were identified in the *Sub1B* gene by re-sequencing of 110 cultivars where the gene could be amplified. Two of these SNPs are in the 5′ upstream region, one in the exon and four in the intron (Fig. 4). The seven SNPs made nine gene haplotypes and two protein haplotypes. All the SNP haplotypes of *Sub1B*, except H4, code for protein haplotype 1 (base guanine at position 280, SNP3), while H4 (base adenine at position 280, SNP3) codes for protein haplotype 2 (Table 5). This functional mutation leads to a change in amino acid residue from arginine_94_ in the reference protein haplotype 1 to tryptophan_94_ in protein haplotype 2 (Supplementary Table S9). The *Sub1C* gene amplified and sequenced from 174 genotypes revealed 38 high-quality variations (34 SNPs and 4 InDels), seven of which including two InDels were in the 5′ upstream region and 31 were in the exon (Fig. 4). Total size of the *Sub1C* amplicon sequenced was 1866 bp, including 110 bp of 5′ upstream region, 708 bp of exon and 1048 bp of 3′ downstream region but the 3′ non-coding region was not considered for SNP haplotype analysis. Excluding the downstream region total 67 SNP haplotypes were identified resulting in 36 protein haplotypes (Supplementary Tables S8 and S9).

**Table 4:** Nucleotide positions of seven high-quality SNPs with respect to the ATG start codon in the *Sub1A* gene and SNP haplotype frequencies in 96 rice cultivars from which the gene could be amplified and sequenced.

Haplotype	Exon		Intron					Freq-uency
	SNP1	SNP2	SNP3	SNP4	SNP5	SNP6	SNP7	
	556	678	878	1121	1127	1277	1407	
H1	C	G	G	G	C	A	G	3
H2	C	G	C	G	C	A	G	28
H3	T	G	C	G	C	A	G	9
H4	T	A	C	G	T	A	G	1
H5	T	A	C	G	C	A	G	4
H6	T	A	C	G	C	G	G	1
H7	T	A	C	G	C	G	A	1
H8 (Ref.)	T	A	C	C	C	G	A	49

**Table 5:** Nucleotide positions of seven high-quality SNPs with respect to the ATG start codon in *Sub1B* gene and SNP haplotype frequencies in 110 cultivars from which the gene could be amplified and sequenced.

Haplotype	Promoter		Exon	Intron				Freq-uency
	SNP1	SNP2	SNP3	SNP4	SNP5	SNP6	SNP7	
	−643	−282	280	771	835	855	864	
H1	T	T	C	T	T	A	C	1
H2	C	G	C	T	T	A	C	1
H3	C	G	C	T	G	G	C	1
H4	C	T	T	T	T	A	C	4
H5	C	T	C	C	T	A	C	1
H6	C	T	C	T	T	A	C	61
H7	C	T	C	T	G	A	C	1
H8	C	T	C	T	G	G	C	4
H9 (Ref.)	C	T	C	T	G	G	T	37

**Figure 6:**
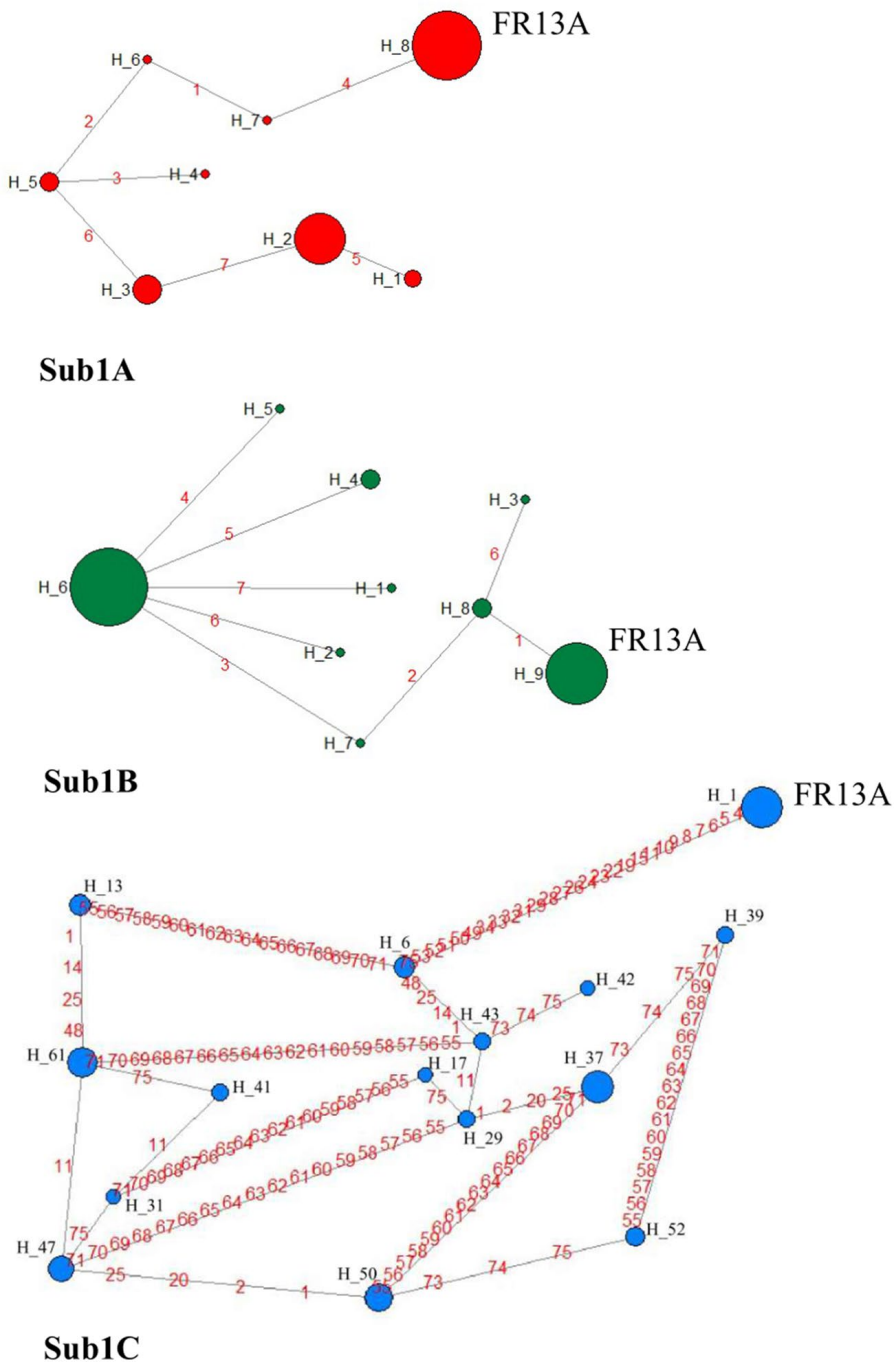
Haplotype networks for *Sub1A*, *Sub1B* and *Sub1C* genes. Size of the circles reflects allele frequency in the cultivar set. Haplotype names (H1, H2…) correspond to that in Tables 4, 5 and Supplementary Table S8.

Initially, haplotype analysis was performed directly based on the nucleotide sequence of *Sub1A*, *Sub1B* and *Sub1C* genes using DnaSP programme. Haplotypes were identified (http://cmpg.unibe.ch/software/arlequin35) and networks were constructed using Hapstar software version 0.6^46,47^. But, due to presence of additional InDels and heterozygous calls, much larger number of haplotypes was obtained (39 each for *Sub1A*, *Sub11B* and 140 for the *Sub1C*, (data not shown). Hence, for simplicity only manually curated high-quality variants, i.e. eight haplotypes for *Sub1A* gene in 96 cultivars, nine haplotypes for *Sub1B* gene in 110 cultivars and 67 haplotypes for *Sub1C* gene in 174 cultivars were considered for the network analysis. Although 38 high-quality SNPs/InDels in *Sub1C* gene of 174 cultivars formed total 67 haplotypes (Supplementary Table S8), network analysis was performed only on 15 haplotypes with genotype frequencies of three or more for a clear visualization of the network (Fig. 6). Network analysis of *Sub1A* gene clearly showed the evolutionary relationship and frequencies of its eight haplotypes (Fig. 6, Sub1A), where H5 was the ancestral haplotype from which other haplotypes have evolved through three independent routes. The most frequent haplotype H8 representing tolerant cultivar FR13A seems to have evolved most recently from the ancestral haplotype H5 through H6 and H7. Submergence sensitive haplotype H2 has followed a different evolutionary route from H5 through H3 and has further diversified into H1, whereas H4 has evolved directly from H5. Network analysis of *Sub1B* showed that the most frequent haplotype H6 representing 61 cultivars is the ancestral type from which other haplotypes have evolved through five independent routes (Fig. 6, Sub1B). The second most frequent haplotype H9, representing 37 cultivar including reference variety FR13A has evolved from H6 through H7 and H8 and has further diverged into H3. Haplotypes H1, H2, H4, and H5 have evolved directly from H6. Five of the nine haplotypes were present in just one cultivar each while H4 and H7 were present in 4 cultivars each (Table 5). Network analysis of *Sub1C* gene based on 15 most frequent haplotypes showed that the H47 was the most ancestral haplotype from which other haplotypes have evolved through multiple complex routes (Fig. 6, Sub1C). The reference variety FR13A possessed haplotype H1, which seems to have evolved recently through H61, H13 and H6. The most frequent haplotypes of *Sub1C* with genotype frequency of ten or higher were H1, H37, H61 and H50.

### Association of submergence tolerance with *Sub1* haplotypes of the cultivars

**Figure 7:**
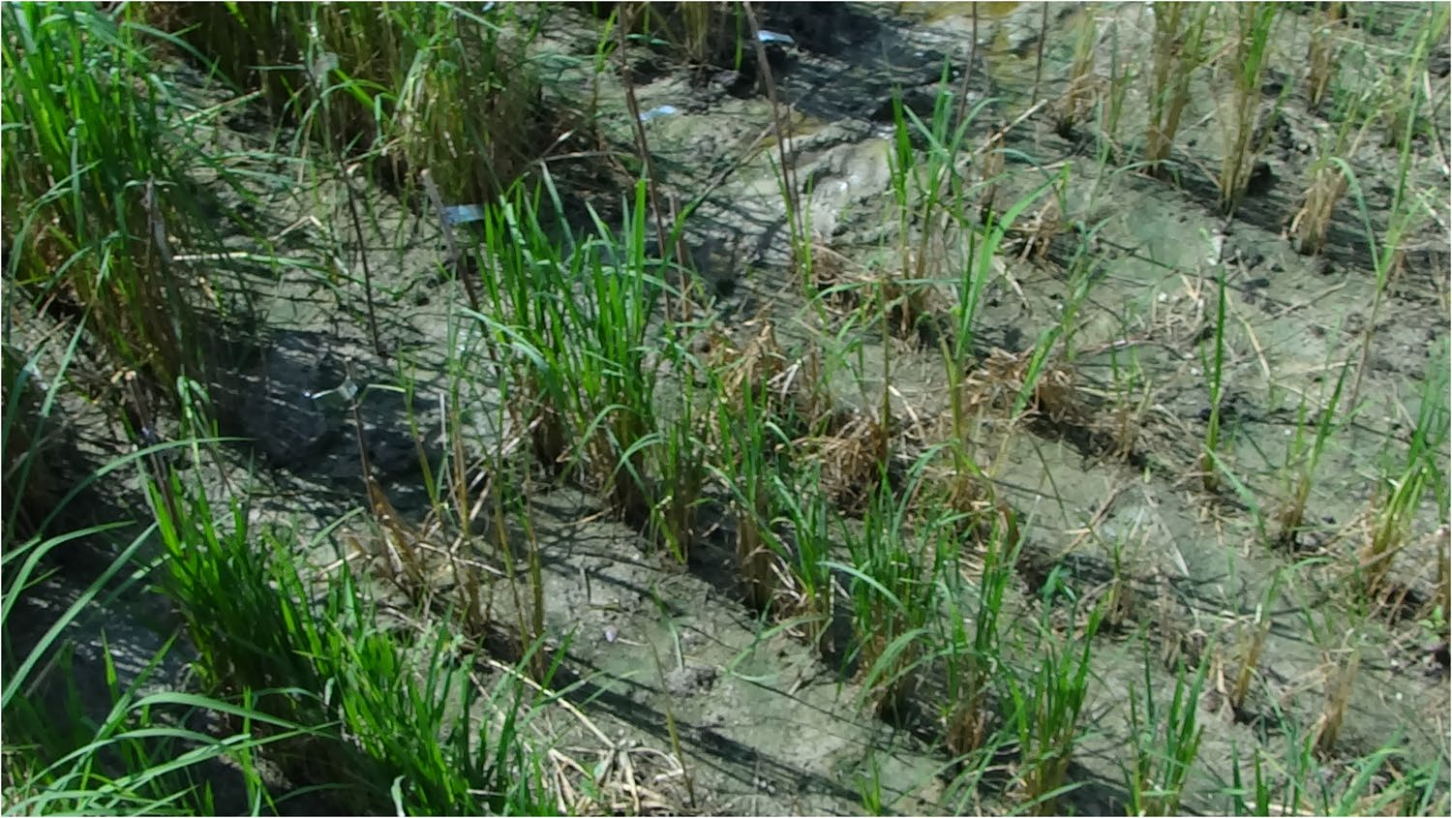
Regeneration of rice cultivars after two weeks of complete submergence in a concrete pond at NDUAT, Ayodhya in 2012. Differential response of the cultivars (planted in single rows) is clearly visible two weeks after de-submergence.

Phenotyping for vegetative stage submergence tolerance was done at four different locations in 2012, including IRRI Manila, NDUAT Ayodhya, BHU Varanasi and RPCAU Samastipur. At NDUAT there were two trials in 2012 one with canopy and another one with cut canopy of elongating plants, also the experiment was repeated here in 2013, thus making total six sets of phenotyping data (Fig. 7, Supplementary Tables S10–S14). Trial conditions for the six experiments varied with respect to the structure of submergence pond, quality of water, age of plants submerged and duration of submergence and recovery, all of which affected the results. Full replications were possible only in IRRI 2012 because of plastic tray based mini plots and NDUAT 2013 due to natural field submergence in a large area. The remaining four experiments followed augmented design with only the check varieties repeated in each block in the submergence ponds. Accordingly, the replication means or adjusted means of survival were used for the analysis of cultivar performance. The stress was most severe at BHU 2012 where average survival rate of 126 genotypes after two weeks of submergence in natural floodwater was the lowest at 11.6% with a range of 0–100%. The stress level was mildest at RPCAU resulting in the highest average survival rate of 84.6% with a range of 42 - 100% for 175 cultivars, likely due to the shortest duration of only 12 days submergence. The stress here was too low to allow proper discrimination between tolerant and sensitive cultivars hence it was not used for further analysis. Stress level in the experiment at IRRI 2012 was optimum providing the best discrimination among 165 cultivars with mean survival rate of 65.9% in a range of 9.8–100%. In NDUAT 2012, average survival rate was 69.4% with a rage of 0–100% for the experiment with normal leaf canopy, which was more than twice the average of 31.3% survival where leaf canopies of elongating cultivars were cut to prevent air breathing. In NDUAT 2013, the stress level was high due to natural flooding with muddy water for two weeks.

**Figure 8:**
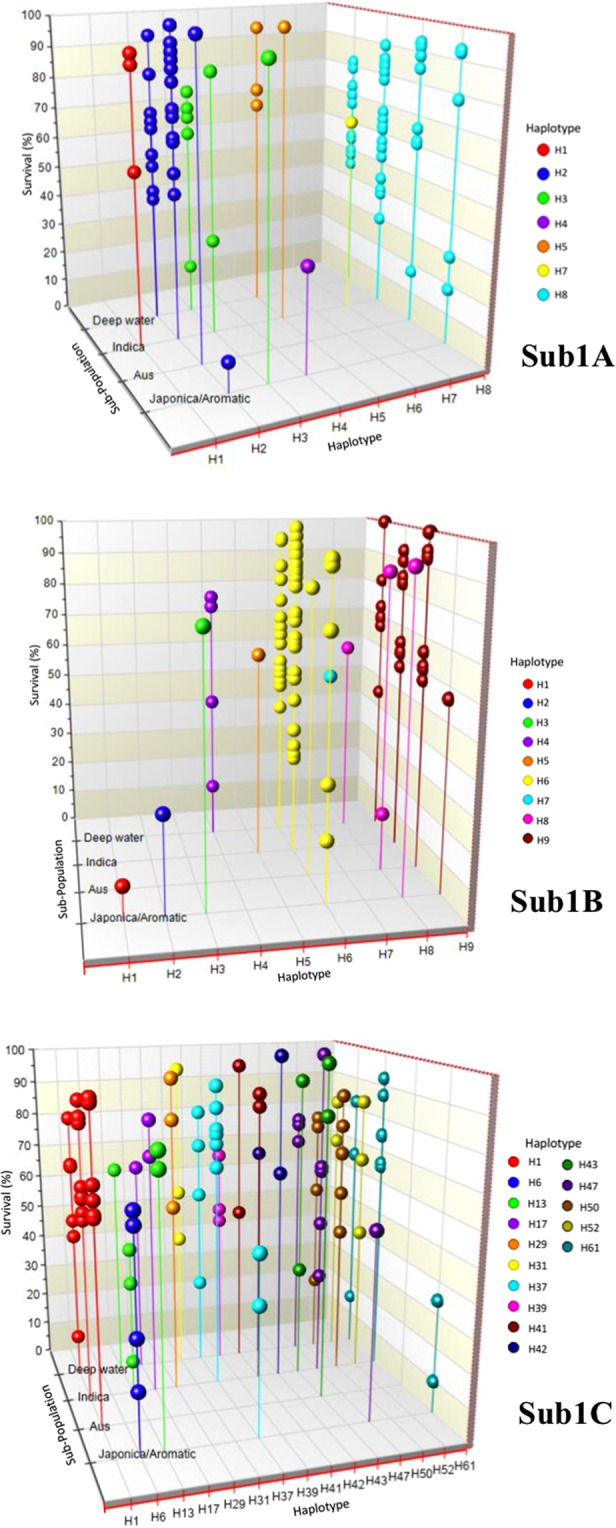
3-D graphs showing relationship between Structure sub-population, SNP haplotype and percent survival of rice cultivars based on submergence tolerance data from IRRI in 2012. Reference alleles of *Sub1A*, *Sub1B* and *Sub1C* genes from tolerant variety FR13A are represented by haplotypes H8, H9 and H1, respectively.

However, despite the variation in trial conditions tolerant checks FR13A, FR43B, Sambha-Sub1 and Swarna-Sub1 showed high rate of survival and susceptible check IR42 showed low rate of survival across the five trials. The stress level at RPCAU with 81 cultivars showing more than 90% survival was non-discriminatory (Supplementary Table S15). Tolerant check FR13A showed 92.1–100% survival and sensitive check IR42 showed 0–35% survival in the five trials. Association between phenotypic performance of the rice cultivars and SNP genotypes was evaluated separately for each site and year because of large variation in the results. Large variation in phenotypic performance was observed for different locations and seasons. Significant association was detected using TASSEL software only with the SNPs in *Sub1C* gene and submergence tolerance data of BHU 2012 and NDUAT 2013 (Supplementary Fig. S4). Failure to validation of the known association of SNPs in the *Sub1A* gene with submergence tolerance in the TASSEL analysis, prompted us to make 3-D plots for graphical visualization of three-way relationship among population structure (Aus, Indica, Deep-water and Japonica-Aromatic cultivar groups), SNP haplotypes and percent survival of the cultivars under submergence stress based on survival data of IRRI 2012 (Fig. 8, Supplementary Table S16). The 3-D plots clearly show that submergence tolerant genotypes were present in all the four sub-populations, but there were proportionately larger number of tolerant genotypes in the Aus group, represented by FR13A. Among the various haplotypes of the *Sub1A*, *Sub1B* and *Sub1C* genes, there was no clear association between haplotype and the level of tolerance. There were exceptions to the known association of tolerant *Sub1A-1* allele (haplotype H8) of FR13A, where nine cultivars with haplotype H8 showed survival rates below 50% (Fig. 8, Sub1A, Supplementary Table S16). Conversely, there were 15 cultivars without haplotype H8 but with more than 80% survival, e.g. six out eight cultivars with haplotype H3 and all four cultivars with haplotype H5 showed above 60% survival. However, both haplotype H3 and H5 have the same protein haplotype as the tolerant haplotype H8. Two cultivars with haplotype H1 and nine cultivars with haplotype H2 (both representing the sensitive allele *Sub1A-2*) showed more than 70% survival. Since *Sub1A-1* (Haplotype H8) is the established tolerant allele, we analyzed all the genotypes using functional allele-specific PCR marker AEX1^25^ to reconfirm the presence of *Sub1A1* allele (Supplementary Table S17). It is expected that all the cultivars with SNP haplotypes H1 and H2 or null allele for the *Sub1A* gene should not give any amplification, whereas haplotypes H3-H8 should produce a PCR amplicon of 231 bp. As expected none of the 31 cultivars with haplotypes H1 and H2 gave AEX product. However, nine out of 83 cultivars without *Sub1A* gene amplification showed AEX amplification, the discrepancy is most likely due to failure of PCR amplification of the *Sub1A* gene in these due to primer mismatches. Forty-one of the 65 cultivars possessing *Sub1A* haplotypes H3-H8, also showed the expected AEX amplification, 24 did not amplify which again could be because of primer mismatches due to mutational changes in the flanking sequences.

For *Sub1B* gene, H9 was the reference haplotype of cultivar FR13A, which together with all other SNP haplotypes coded for protein haplotype 1, except for H4 that coded for protein haplotype 2. Five of the nine haplotypes are represented by one genotype each hence not suitable for association analysis. Of the remaining four, H4 coding for an altered protein and genotype frequency of four showed a range of survival from 15.8% to 89.2%. Haplotype H6 with the highest genotype frequency of 61 also showed a wide range of survival response from 18.4% to 95%, haplotype H8 with genotype frequency of four also showed survival range of 17.5% to 92.5%. Interestingly, all but one of the 37 genotypes possessing reference haplotype H9 showed survival of more than 50%. A clear difference was visible between the survival patterns of cultivars possessing two most frequent *Sub1B* haplotypes H6 and H9 with the reference haplotype H9 showing much higher survival frequency (Fig. 8, Sub1B).

For *Sub1C* gene, the 3D plot was drawn for 15 SNP haplotypes with genotype frequencies of three and above (Fig. 8, Sub1C). The reference haplotype H1 representing tolerant cultivar FR13A was present in the highest number of 22 cultivars, 20 of which showed survival above 50%, 17 genotypes above 60% and 9 genotypes above 80%. Thus, H1 was clearly associated with submergence tolerance with very few exceptions. Another common haplotype H37 was present in 14 cultivars but showed variable level of survival from 26–92.1% with seven of them showing survival above 75%. Haplotype H50 was present in 11 cultivars but only two of these showed survival higher than 75%. Another frequent haplotype H61 was present in 13 cultivars only three of which showed survival above 75%. Among minor alleles, haplotypes H41, H43 and H52 with genotype frequencies of 4, 4 and 5, respectively have more than three-fourth of the genotypes with survival above 80%, whereas most of the cultivars with haplotypes H6, H13, H39 and H47 showed poor survival.

### Novel non-*Sub1A-1* sources of submergence tolerance

A comparison of the best performing entries across five trials at three different locations at IRRI, NDUAT and BHU identified cultivars showing consistently high level of submergence tolerance across locations and trial conditions (Table 6, Supplementary Table S15). The best four cultivars, showing consistent high level of submergence tolerance in each of the five trials, were Madal, Mahadikwee, S-138 and Singhara. Another group of 10 cultivars showing high level of tolerance in four out of five trials included, ARC 614, Dholmon 64-3, Goda Heenati, Heen Sulai, Houra Kani, Kariyawa, Kaluwee, Naldak, Nirboi, and Tetep. Further 18 cultivars, namely Ausboro, Birpala, CR1009, IR 64, Jaldungi, Kajarahawa, Karkati 87, Karnal Local, Kasalath, Menyar Hunei, Naaluumoli Karuppan, Paiaim, Pakri Buna, Panidubi, Pankee, Pataim, Rajbhog and Sangkhla showed high level of submergence tolerance in three out of the five trials. These 32 cultivars can be considered as having consistently high level of submergence tolerance. A look at the *Sub1* gene haplotypes of these cultivars revealed that five of these cultivars have the *Sub1A*, *Sub1B* and *Sub1C* haplotype combination H8, H9, H1 that is identical to the tolerant cultivar FR13A (Table 6). Another nine cultivars even though having different haplotypes for the *Sub1B* and *Sub1C* genes, possessed either identical (H8) or synonymous haplotypes (H3-H7) for the *Sub1A* gene (Table 6), and therefore possess the same *Sub1A-1* dependent submergence tolerance. The remaining 18 cultivars were either null or possessed the sensitive allele *Sub1A-2* (haplotypes H1 and H2) of the *Sub1A* gene (Table 6). Hence, these must possess *Sub1A-1* independent mechanism of submergence tolerance. In fact 10 of the 14 cultivars showing consistent submergence tolerance in four to five trials appear to possess non-*Sub1A1* type submergence tolerance (Table 6).

**Table 6:** Submergence tolerant rice cultivars showing consistent performance in five different trials across three locations, along with population Structure (**1**. Deep-water, **2**. Indica, **3**. Aus, **4**. Aromatic-Japonica), AEX marker amplification and *Sub1* SNP haplotypes. Bold and normal fonts indicate *Sub1A-1* dependent and *Sub1A-1* independent tolerance, respectively.

Sr. no.	Cultivar	Struct-ure group	No. of trials with tolerance	AEX ampli-fication	SNP haplotype		
					*Sub1A*	*Sub1B*	*Sub1C*
1.	Madal	2	5	−	−	H6	H29
**2**.	**Maha Dikwee**	**2**	**5**	−	**H5**	**H6**	**H2**
3.	S-138	2	5	−	H2	H6	H61
4.	Singhara	2	5	−	H1	H6	H43
5.	ARC 614	1	4	−	H2	H6	H31
6.	Dholmon 43-3	1	4	−	H2	H6	H61
7.	Goda Heenati	2	4	−	−	H6	−
**8**.	**Heen Sulai**	**2**	**4**	−	**H8**	**H9**	**H1**
9.	Houra Kani	1	4	−	−	H6	H45
10.	Kariyawa	2	4	−	H1	H6	H29
11.	Kaluwee	2	4	−	H2	H6	H60
**12**.	**Naldak**	**2**	**4**	−	**H8**	**H9**	**H1**
13.	Nirboi	1	4	−	−	H6	H61
**14**.	**Tetep**	**2**	**4**	−	**H3**	**H6**	**H41**
**15**.	**Ausboro**	**3**	**3**	**+**	**H8**	**H9**	**H1**
**16**.	**Birpala**	**1**	**3**	−	**H3**	**H6**	**H26**
17.	CR 1009	2	3	−	H2	H6	H17
18.	IR 64	2	3	−	H2	H6	H29
19.	Jaldungi	1	3	−	H2	H6	H45
20.	Kajrahawa	3	3	−	H2	H9	H43
**21**.	**Karkati 87**	**2**	**3**	−	**H8**	**H6**	**H52**
**22**.	**Karnal Local**	**4**	**3**	**+**	**H8**	**H8**	**H11**
**23**.	**Kasalath**	**3**	**3**	**+**	**H8**	**H9**	**H1**
**24**.	**Menyar Hunei**	**1**	**3**	−	**H8**	**H6**	**H15**
25.	Naalumoli Karuppam	2	3	−	−	−	H3
26.	Paiaim	2	3	−−	−	−	H37
**27**.	**Pakri Buna**	**1**	**3**	−	**H8**	**H8**	**H24**
**28**.	**Panidubi**	**1**	**3**	**+**	**H8**	**H9**	**H56**
**29**.	**Pankei**	**1**	**3**	**+**	**H8**	**H9**	**H1**
**30**.	**Pataim**	**1**	**3**	−	**H5**	**H6**	**H41**
31.	Rajbhog	2	3	−	−	H6	H42
32.	Sang Khla	1	3	−	−	−	H37

## Discussion

Utilization of the *Sub1* QTL in development and commercialization of submergence tolerant versions of high-yielding rice cultivars is a remarkable success story of genomics-assisted breeding for accelerated transfer of climate resilience from traditional varieties into green revolution varieties^25,48,49^. However, there are different kinds of flooding stresses affecting establishment and yield of rice including submergence during germination, water stagnation for extended period and deep-water flooding that need attention^16^. Also, there are *Sub1*-independent mechanisms of complete submergence tolerance that need to be explored and harnessed. There is limited number of studies on allelic sequence variation in the three *Sub1* genes. This includes sequence survey of the three *Sub1* genes in 17 Indica and four Japonica cultivars^19^, 14 accessions of cultivated and three accessions of wild rice *O. rufipogon*^20^, marker survey of *Sub1A* and *Sub1C* alleles in 109 rice genotypes and sequencing of *Sub1* gene orthologs in two accessions of *O. eichingeri* and *O. rhizomatis*^21^, marker survey of *Sub1A* and *Sub1C* alleles in 76 rice cultivars^50^, and CAPS marker survey of *Sub1A* and *Sub1C* genes in 160 rice varieties^22^. Whole genome sequence data is available for 3000 rice varieties from 3 K RGP project^45^ and 66 rice genomes including 14 wild rice accessions^23^, but these have not been analysed for sequence variation in the *Sub1* genes. Thus, present study on the analysis of sequence variation in *Sub1A*, *Sub1B* and *Sub1C* genes of 179 diverse rice cultivars grown in the flood prone areas and its association with submergence tolerance provides novel information.

Genetic diversity and population structure of uncharacterized flood tolerant rice germplasm is an essential pre-requisite for association studies for identification of new QTLs and genes. Germplasm characterization using high-throughput genotyping methods and development of breeding strategy based on this has already started in rice^51,52^. Structure analysis of 179 such cultivars in this study classified them into two major sub-populations, representing (i) Japonica and Aromatic cultivar groups, and (ii) Indica and Aus cultivar groups. Four cultivars in sub-population 1 and 14 cultivars in sub-population 2 showed inferred ancestry of less than 80% of the group they belonged, indicating admixture types. It was interesting that Structure analysis at K value of four separated all the 14 admixture types from Indica sub-population and three out of four admixture types from Aromatic-Japonica sub-population to make a separate group of 17 Aus cultivars each with a high Fst value of greater than 0.80. This indicates that Aus cultivars share common ancestry with both Indica and Japonica groups and may provide a link between the two. Aus cultivars are important source of genes for tolerance to abiotic stress including flood and drought. They have short duration, adaptation to multiple stresses and are grown all over Indian sub-continent and adjoining regions of South-East Asia. Aromatic cultivars are known to be closer to Japonica group and Aus cultivars are closer to Indica group^51,53,54^. Isozyme-based diversity analysis by Glasszmann^54^, classified rice into six cultivar groups, with only one Japonica group (combining tropical and temperate Japonica) but two additional groups of *Rayada* and *Ashina*, representing deep water and floating rice of Bangladesh and India, respectively. Studies with large number of genomic SSR/SNP markers have reported five groups of rice cultivars, namely Indica, Tropical Japonica, Temperate Japonica, Aromatic and Aus^51,52^. Our results are in closer agreement with the classical work of Glaszmann^54^ employing 15 isozyme marker loci, likely because we used 36 SNP markers from conserved single-copy genes distributed on the 12 rice chromosomes.

Analysis of sequence polymorphism in the functional genes for agronomic traits is necessary for identification of superior alleles in the germplasm. Map-based cloning of *Sub1A* gene and its functional validation by genetic transformation has proven its role in submergence tolerance of rice^19^. But after the original work, which also included sequencing of *Sub1A*, *Sub1B* and *Sub1C* genes from 21 rice cultivars, no further studies have been undertaken on allelic sequence variation in the *Sub1* genes, although marker based allele surveys have been done for *Sub1A* and *Sub1C* genes in larger sets of cultivars^21,22,50^. Here, we generated high-quality sequence information by targeted re-sequencing of pooled PCR products from 96 cultivars for *Sub1A*, 110 cultivars for *Sub1B* and 174 cultivars for *Sbu1C* gene, resulting in comprehensive allelic sequence information. We also analysed the publicly available whole genome sequence data on 66 rice genomes for this purpose. These together identified 9 SNPs in *Sub1A*, 37 SNPs in *Sub1B* and 56 SNPs plus four InDels in *Sub1C* gene. Of these, one, four and 10 SNPs were unique to the 179 cultivars set, whereas two, 29 and 22 SNPs were unique to the 66 genomes set, respectively. Most of the unique SNPs in the 66 genomes set were due to 14 wild rice accessions (Supplementary Table S5), suggesting that our res-sequencing work has provided a comprehensive coverage of the *Sub1* gene sequence variation in rice cultivars.

Based on the seven SNPs each in the *Sub1A* and *Sub1B* genes and 38 SNPs/InDels in the *Sub1C* gene in 179 cultivars, eight, nine and 67 SNP haplotypes were identified, respectively. Many of these haplotypes were rare represented by only one or two cultivars. The number of SNPs in the exonic regions was limited to 2 in *Sub1A*, 1 in *Sub1B* but it was very high 29 in *Sub1C* gene. The high level of variation in *Sub1C* gene is indicative of its ancient origin resulting in accumulation of more mutations over time as compared to *Sub1A* and *Sub1B*. This was also supported by our BLASTX search results showing that *Sub1A* and *Sub1B* gene homologs were limited to genus *Oryza* only, while *Sub1C* homologs were present in other distant genera including *Zostera*, *Mucuna*, *Torenya* and *Nicotiana*, indicating their ancient origin. The ML based phylogenetic tree of the three *Sub1* genes from 13 rice cultivars, rooted with a *Sub1C* homolog from *S. bicolor*, also indicated the evolution of *Sub1B* and *Sub1A* genes by duplication and divergence of the ancestral *Sub1C* gene at the most 12.40 Mya and 17.0 Mya, respectively (Fig. 5). Fukao *et al*.^20^ has also suggested evolution of *Sub1A* gene by duplication and divergence from *Sub1B* which in turn has evolved from duplication of *Sub1C*. Major haplotypes of the three genes were present in both Indica and Japonica sub-populations suggesting no substantial barrier for gene flow across the two groups. The reported absence of *Sub1A* gene in Japonica cultivars has been because of limited number of Japonica varieties analysed in the earlier allele survey studies^19^.

No consistent association was found between SNPs in the *Sub1* genes and percent survival after submergence of 179 cultivars by TASSEL analysis. Only significant association was obtained with SNPs in the *Sub1C* gene and submergence tolerance data from BHU 2012 and NDUAT 2013, where stress intensity was the highest with average survival rates of 11.6% and 31.3%, respectively (Supplementary Fig. S4). Whether or not this association is real need further validation using precise phenotyping repeated in different locations/seasons. Surprisingly, the known association of the *Sub1A-1* allele with submergence tolerance was not validated in the present study due to presence of several exceptions in the present cultivar set. This was also reflected in a direct visualization of data in 3D plots (Fig. 8), where nine cultivars with the tolerant *Sub1A-1* allele (haplotype H8) showed poor survival after submergence. On the other hand several cultivars with the sensitive *Sub1A-2* allele (haplotype H2) showed high level of tolerance across locations (Fig. 8, Table 6). There are at least six published reports of *Sub1A-1-*independent mechanisms of submergence tolerance in rice^5,9,14,21,50,55^. Supporting our TASSEL results of significant associations with *Sub1C* SNPs at two locations, the 3D plot also showed only one exception to the association between submergence tolerance and tolerant Sub1C allele (haplotype H1). However, these exceptions need further analysis in bi-parental segregating populations. Importance of *Sub1-*independent mechanism of submergence tolerance has been highlighted decades ago^56^ and major non-*Sub1* QTLs have been identified using Madabaru/IR72 and Chehrang-Sub1/IR10F365 populations, suggesting *Sub1*-independent mechanism of vegetative stage submergence tolerance^14,21^.

To conclude, we identified several new alleles of the three *Sub1* genes by targeted re-sequencing of 179 rice cultivars. Further, we identified 32 cultivars showing consistently high level of submergence tolerance in five trials at three different locations. Fourteen of these cultivars possessed the known *Sub1A-1* dependent submergence tolerance, but 18 cultivars have *Sub1A-1* independent submergence tolerance as they are either null for the *Sub1A* or possess submergence sensitive allele *Sub1A-2* (haplotypes H1 and H2), although cultivar Goda Heenati is shown in other studies to possess the tolerant allele^19^. There is a need to identify the submergence tolerance QTLs and underlying genes in these cultivars using bi-parental mapping populations for effective utilization in rice breeding programme. We also report for the first time presence of *Sub1A* gene in Japonica and Aromatic groups of rice cultivars including Kola Joha and Karnal Local, which will be useful in breeding flood-tolerant aromatic rice cultivars.

## Supplementary information

Supplementary Figures and Tables.

Supplementary Dataset 1.

Supplementary Dataset 2.
