# Supplementary material for: Allelic sequence variation in the Sub1A, Sub1B and Sub1C genes among diverse rice cultivars and its association with submergence tolerance
Source: Sci Rep. 2020 May 25;10:8621. doi: 10.1038/s41598-020-65588-8 (PMC7248102; doi:10.1038/s41598-020-65588-8)
Supplement: Supplementary file 1 — Supplementary Figures and Tables. [file 41598_2020_65588_MOESM1_ESM.docx]

**Supplementary Information: 7 Figures and 17 Tables**

**Allelic sequence variation in the *Sub1A, Sub1B* and *Sub1C* genes among diverse rice cultivars and its association with submergence tolerance**

Anuradha Singh^1,2,3^, Yashi Singh^1^, Ajay K Mahato^1^, Pawan K. Jayaswal^1^, Sangeeta Singh^1^, Renu Singh^1^, Neera Yadav^1^, A. K. Singh^4^, P. K. Singh^5^, Rakesh Singh^6^, Rajesh Kumar^7^, Endang M. Septiningsih^2,8^, H. S. Balyan^3^, Nagendra K. Singh^1^ and Vandna Rai^1*^

^1^ICAR-National Institute for Plant Biotechnology, Pusa Campus, New Delhi, India

^2^International Rice Research Institute, DAPO 7777, Metro Manila, Philippines

^3^Department of Genetics and Plant Breeding, Chaudhary Charan Singh University, Meerut, India

^4^Department of Crop Physiology, Narendra Deo University of Agriculture & Technology, Ayodhya, UP, India

^5^Department of Genetics and Plant Breeding, Banaras Hindu University, Varanasi, India

^6^ICAR-National Bureau of Plant Genetic Resources, Pus Campus, New Delhi, India

^7^Department of Genetics and Plant Breeding, Dr. Rajendra Prasad Central Agricultural University, Samastipur, Bihar, India

^8^Present address: Department of Soil and Crop Sciences, Texas A & M University, TX 77843, USA

*Corresponding author: vandnarai2006@gmail.com; phone number +91-01125841787

Anuradha Singh: singh18anuradha@gmail.com

Yashi Singh: yashisingh15@gmail.com

Ajay K. Mahato: ajaybioinfo@gmail.com

Pawan K. Jayaswal: er.pawanjayaswal@gmail.com

### Sangeeta Singh: sangeeta10mar@gmail.com

Renu Singh: renu.genetics@gmail.com

Neera Yadav: neerayadav10@gmail.com

A.K. Singh: assinghkumar3@gmail.com

P. K. Singh: pksbhu@gmail.com

Rakesh Singh: delhi.singh@gmail.com

Rajesh Kumar: dr_rajesh_1974@yahoo.co.in

Endang M. Septiningsih: eseptiningsih@tamu.edu

H. S. Balyan: hsbalyan@gmail.com

Nagendra K. Singh: nksingh4@gmail.com

**Figure S1**. Flow diagram showing strategy for the amplification and re-sequencing of the three *SUB1* genes using Ion Torrent PGM machine

**
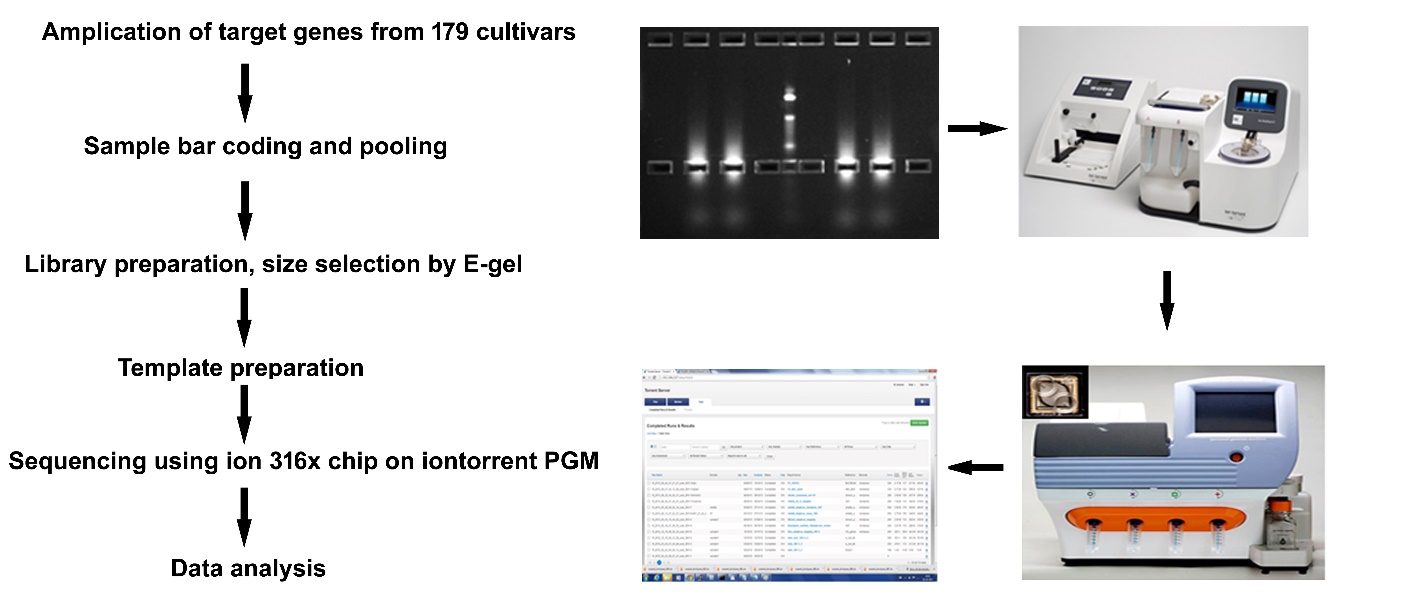
**


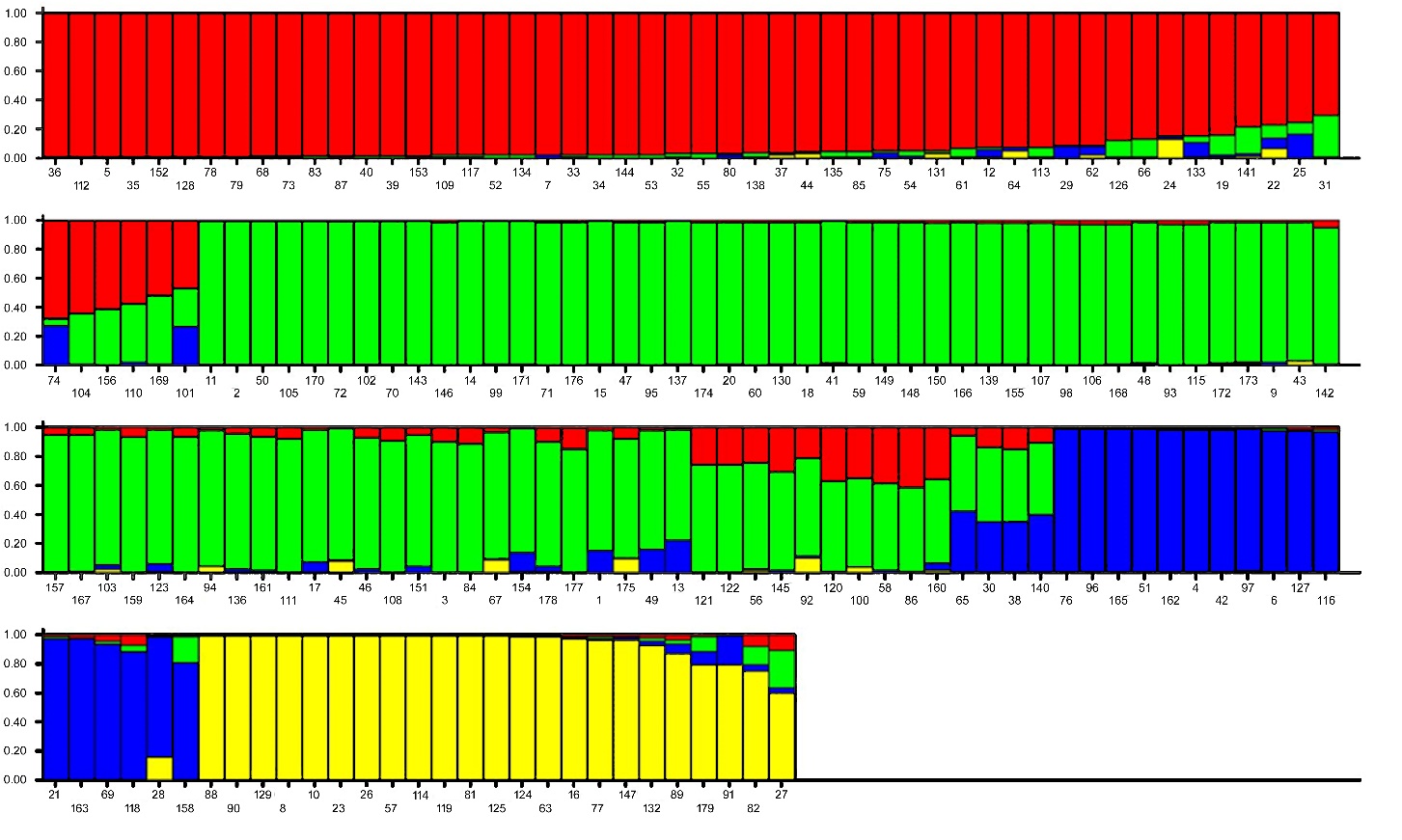

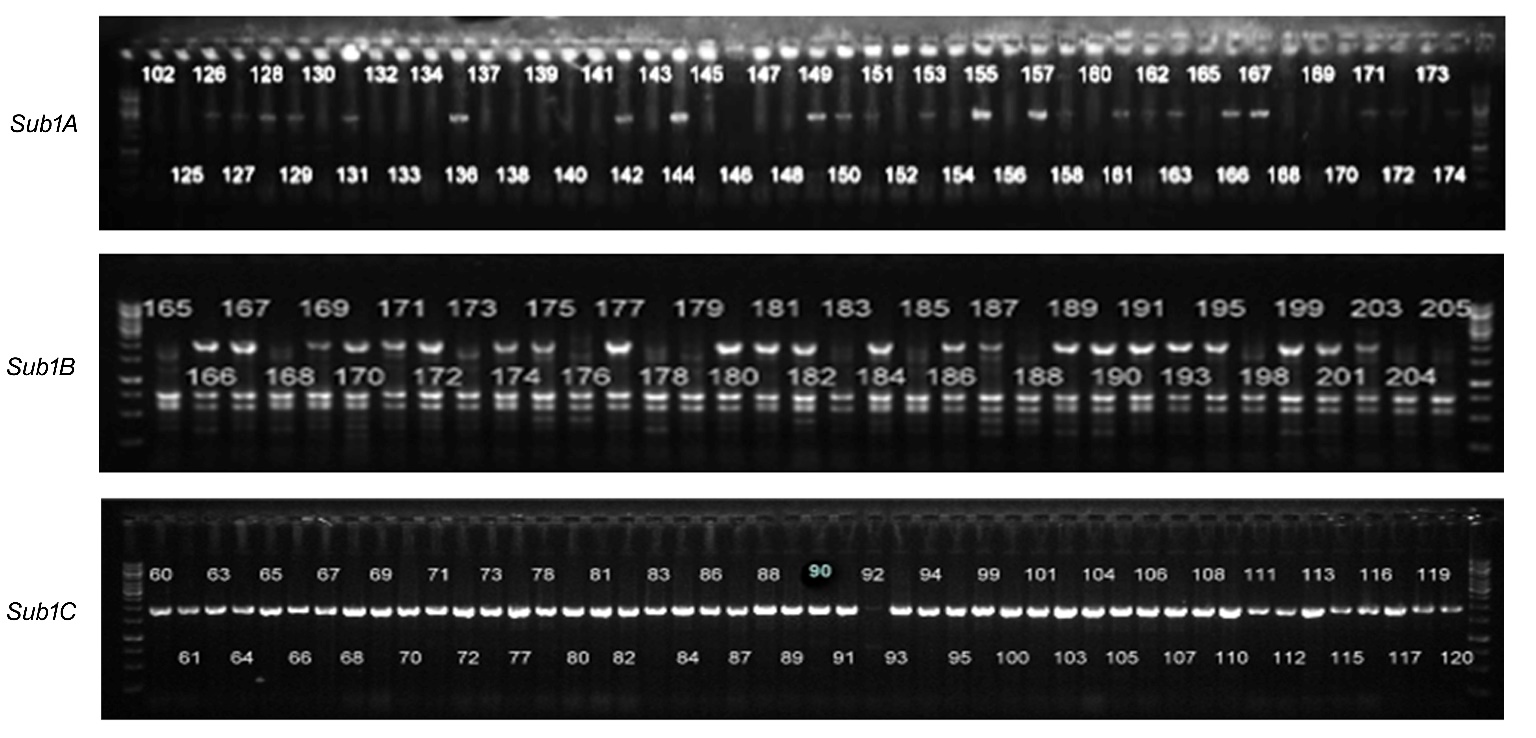


**Figure S2.** Population structure of 179 rice genotypes with *K*=4, showing 4 sub-populations corresponding to *japonica*-*aromatic* (yellow), *aus* (blue), *indica* (green) and *deep water* (red) rice. A number of genotypes, particular in the *indica* sub-group showing admixture of populations.

**Figure S3.** Agarose gel electrophoresis showing variable amplification of the three *Sub1* genes for use in re-sequencing by Ion Torrent PGM sequencing platform.


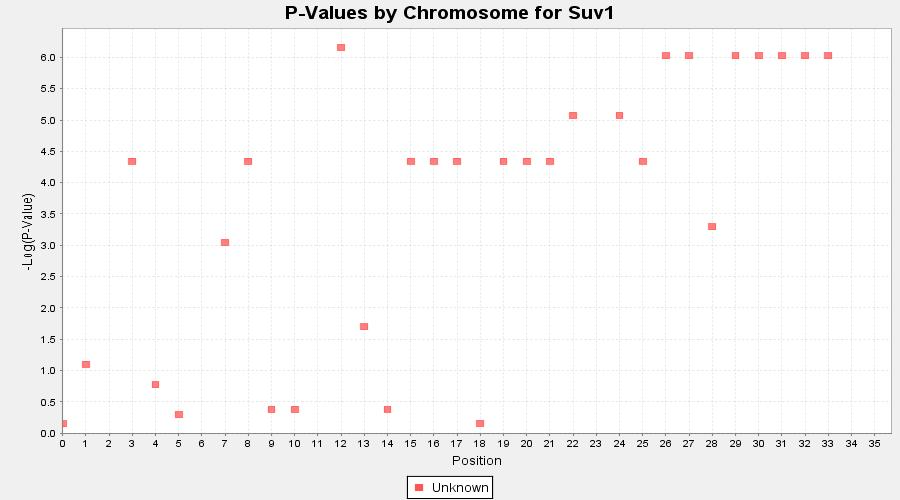

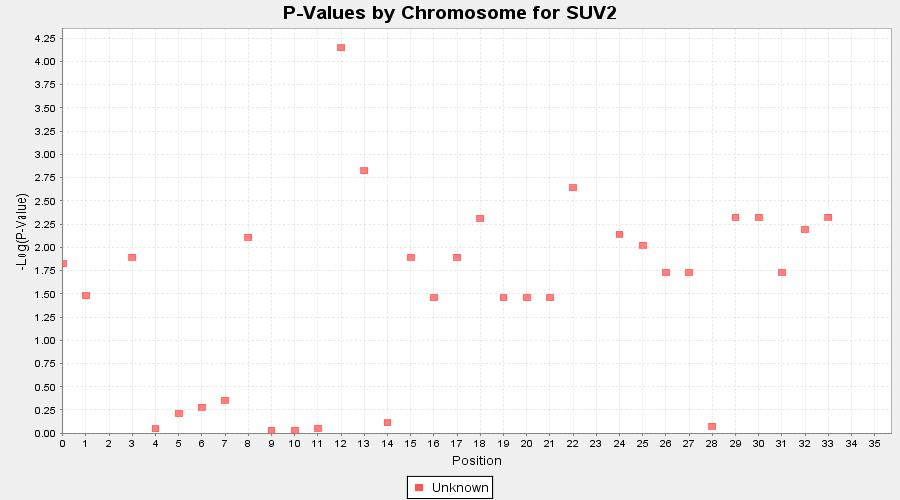


(a)

(b)

**Figure S4.** Significant associations between SNPs in *Sub1C* gene and percent survival after submergence detected using TASSEL The LOD scores of more than 4 suggesting the significant association: (a) BHU 2012, (b) NDUAT 2013


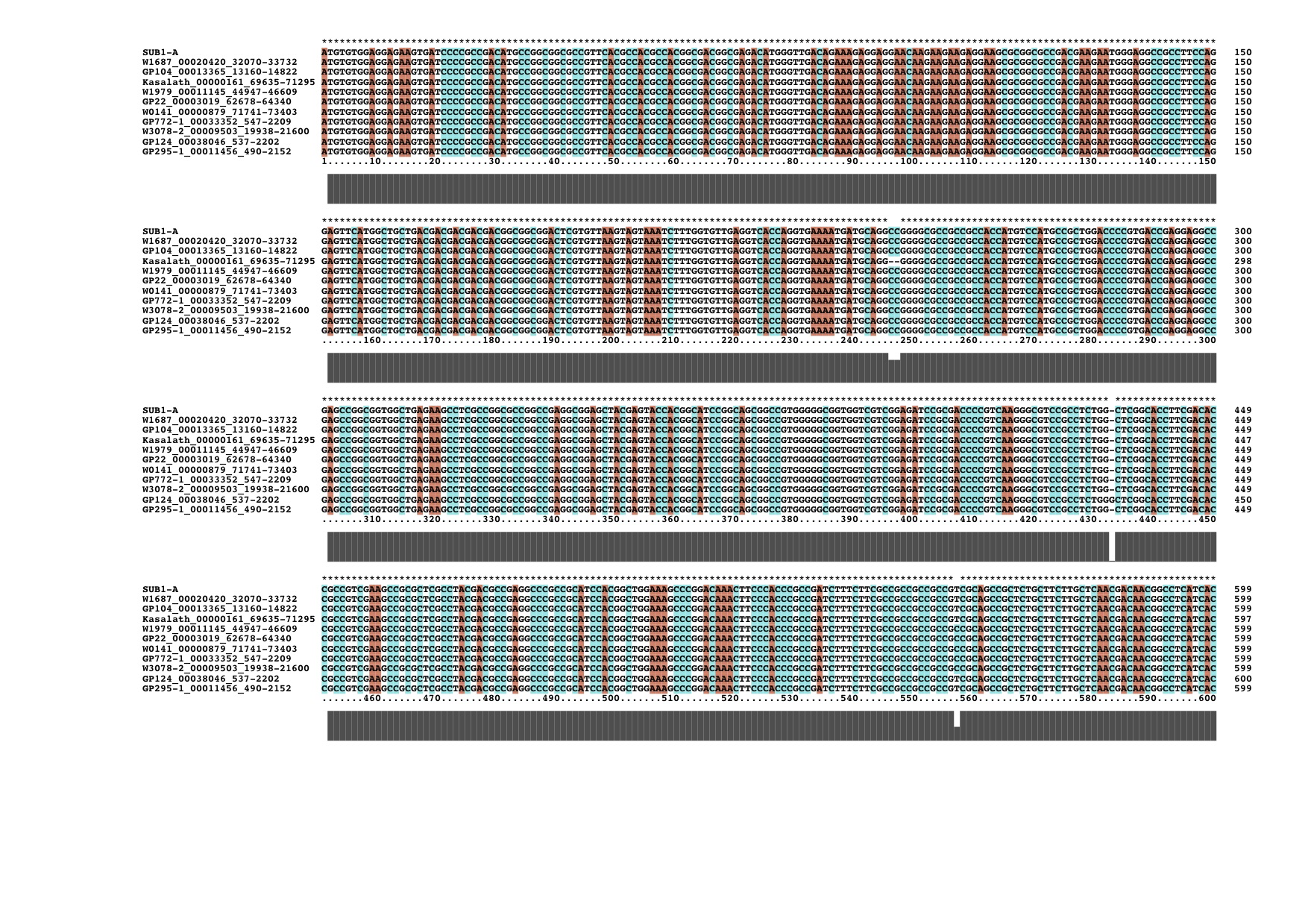


**Figures S5:** Multiple sequence alignment of the *Sub1A* gene in the flood tolerant rice variety FR13A with the sequences present in 66 whole genome sequences of cultivated and wild rice published by Zhao et al. (2018) and the Nipponbare reference genome of rice


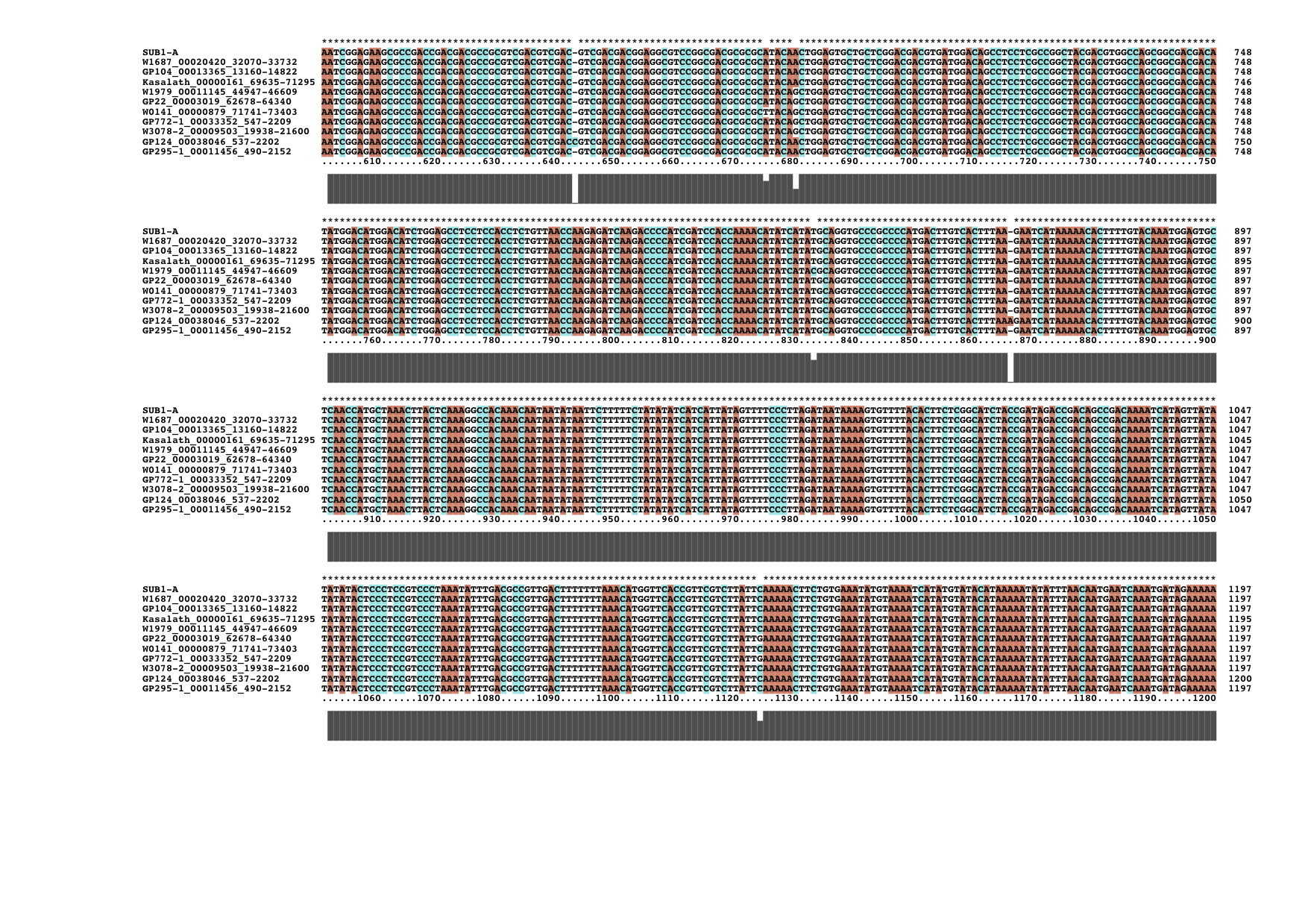


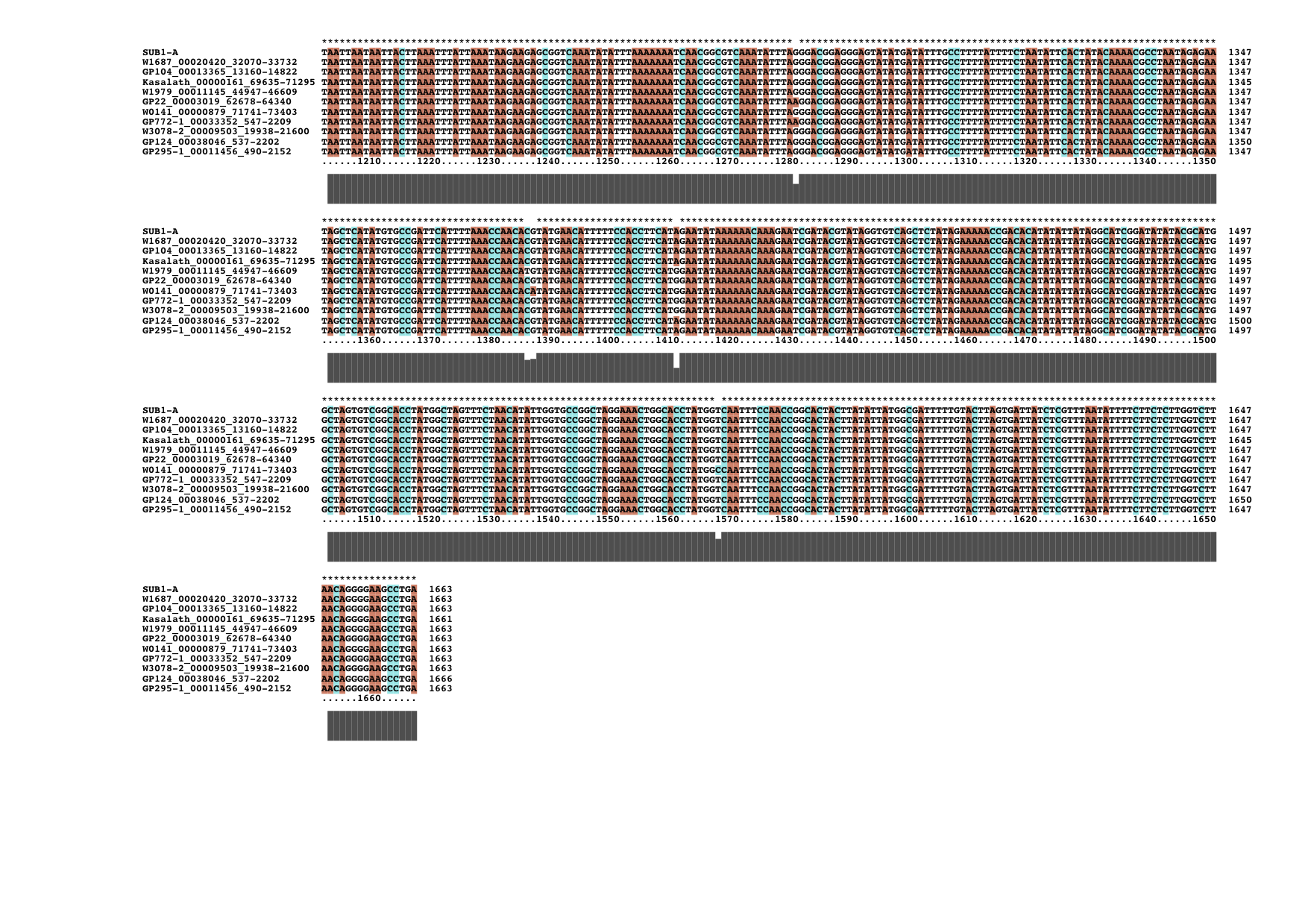


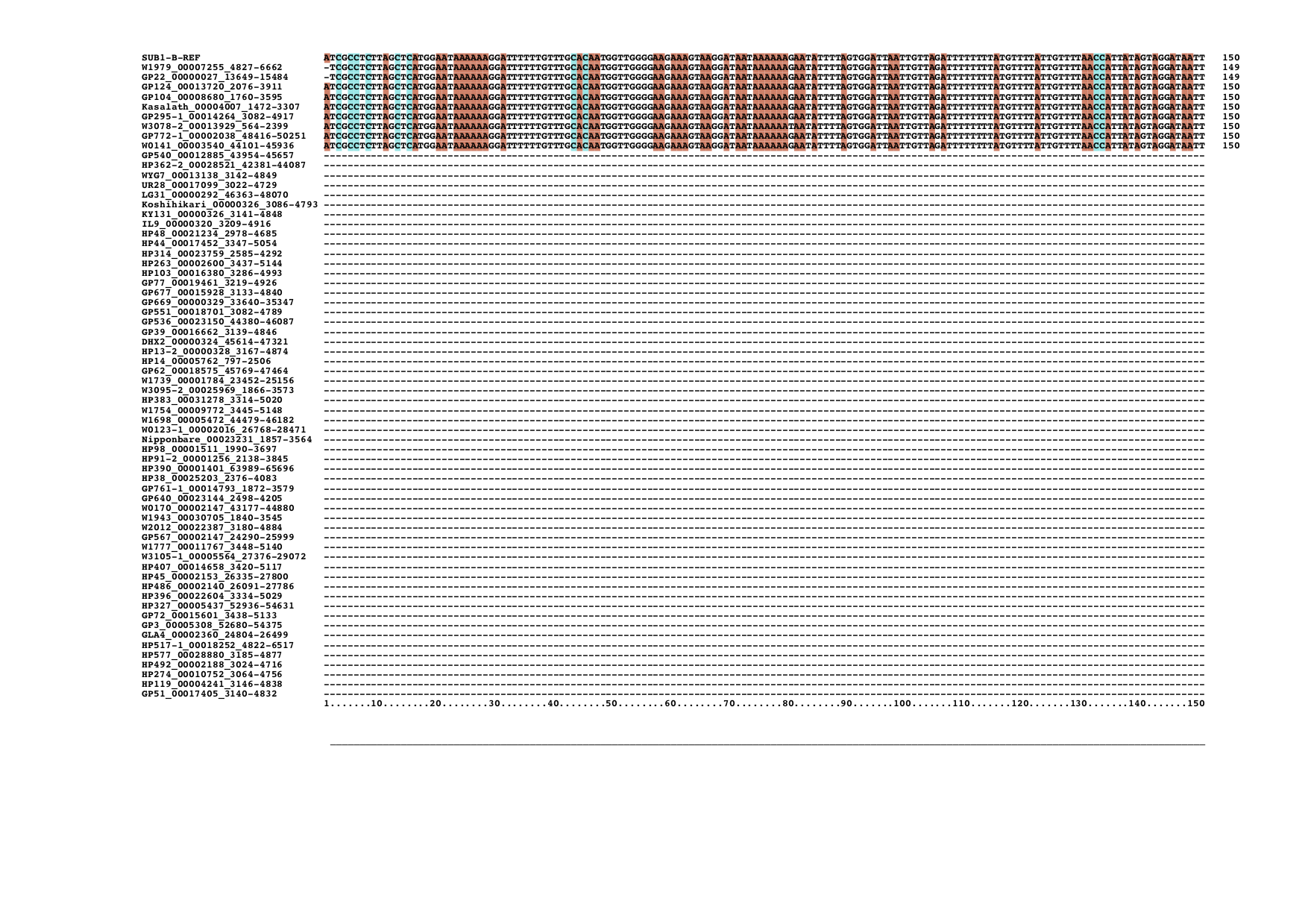


**Figures S6:** Multiple sequence alignment of the *Sub1B* gene in the flood tolerant rice variety FR13A with the sequences present in 66 whole genome sequences of cultivated and wild rice published by Zhao et al. (2018) and the Nipponbare reference genome of rice


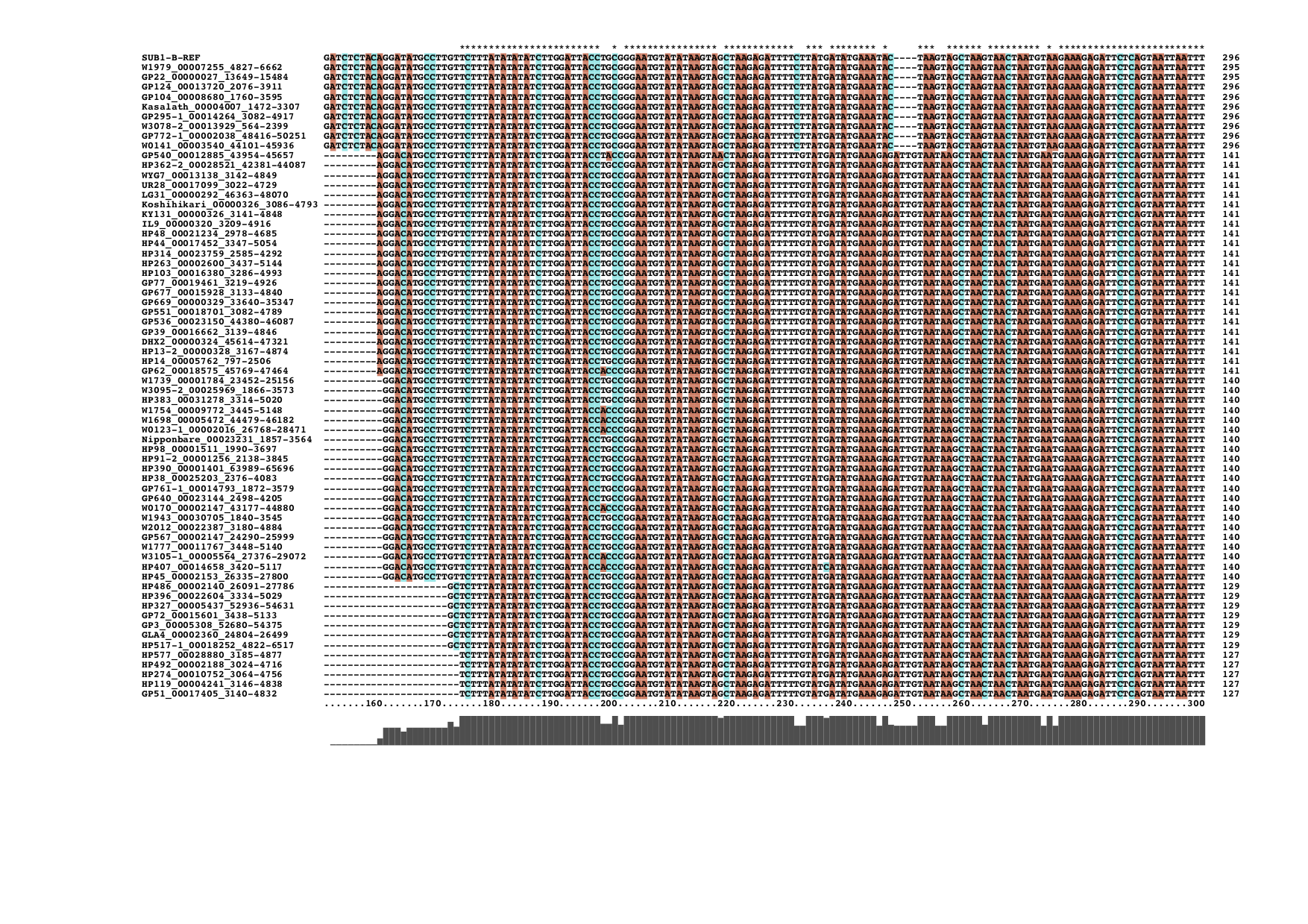


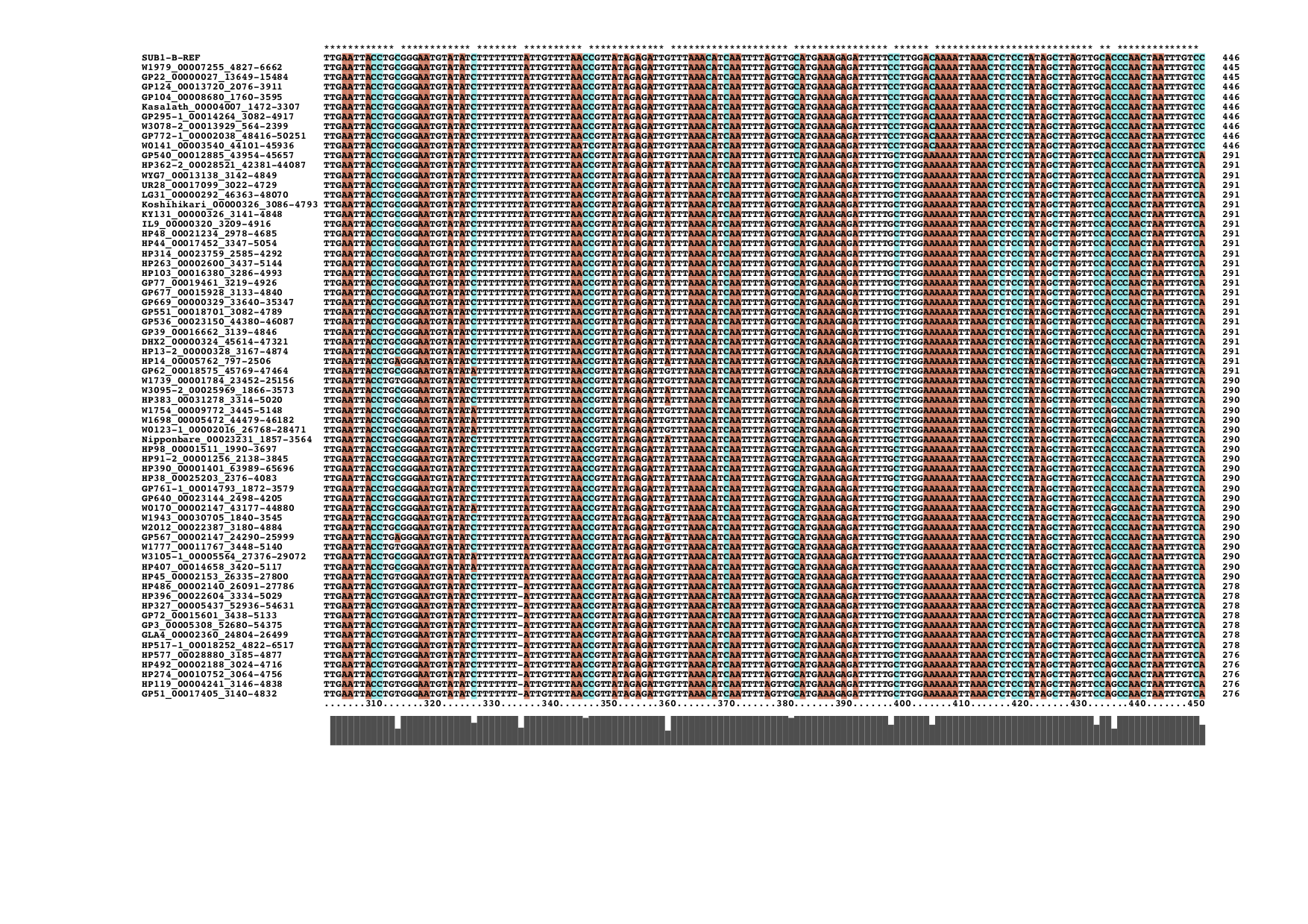


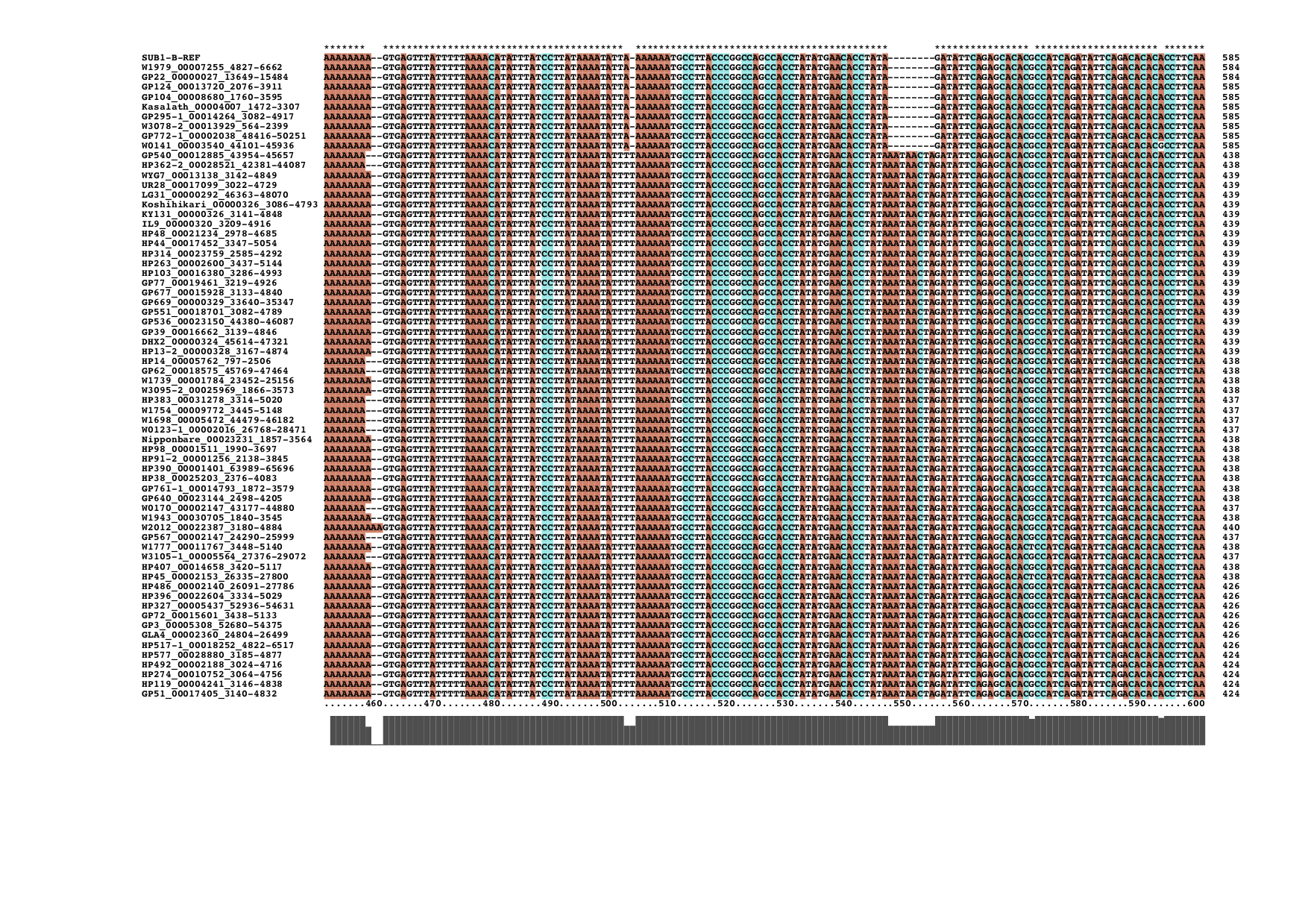


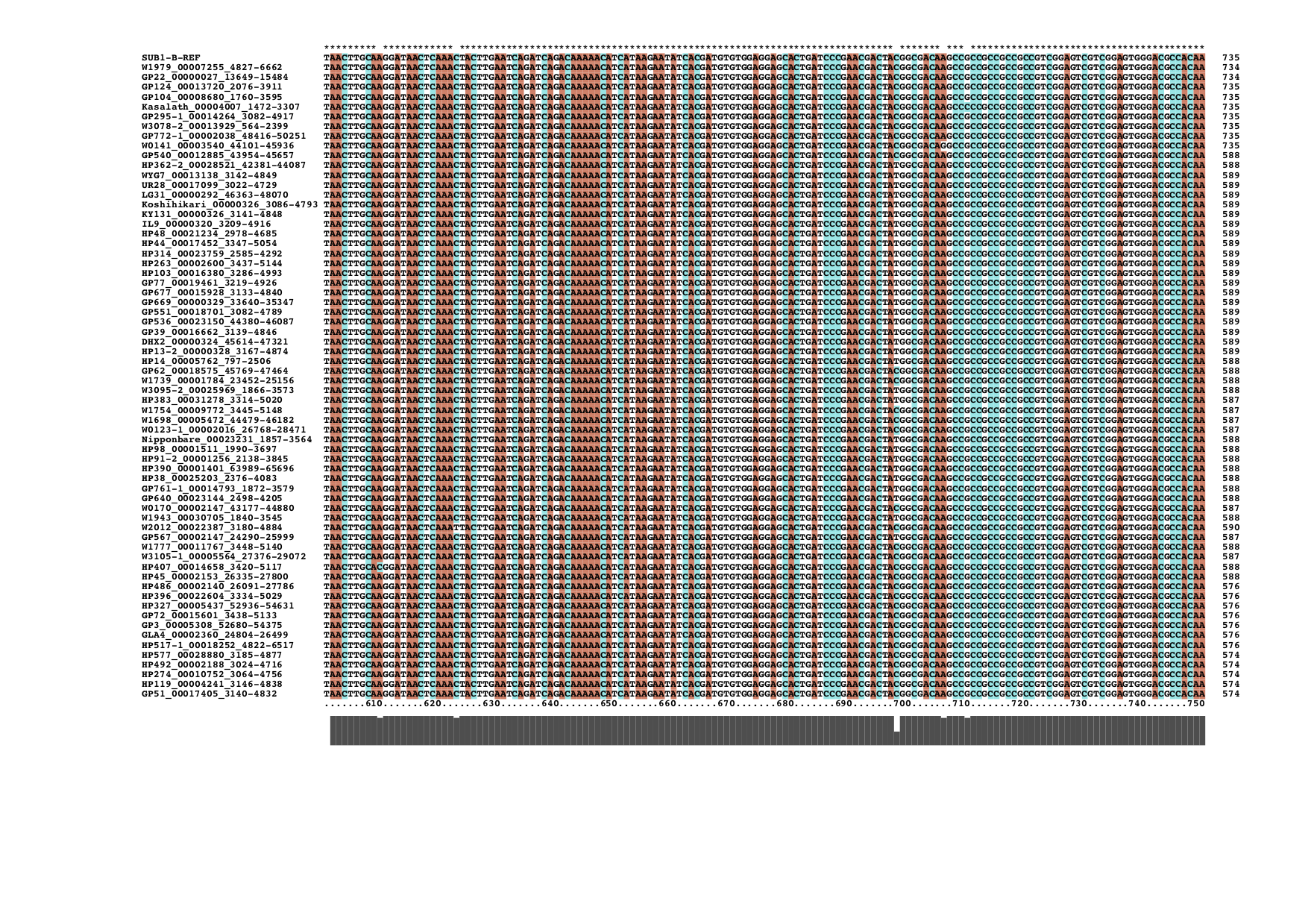

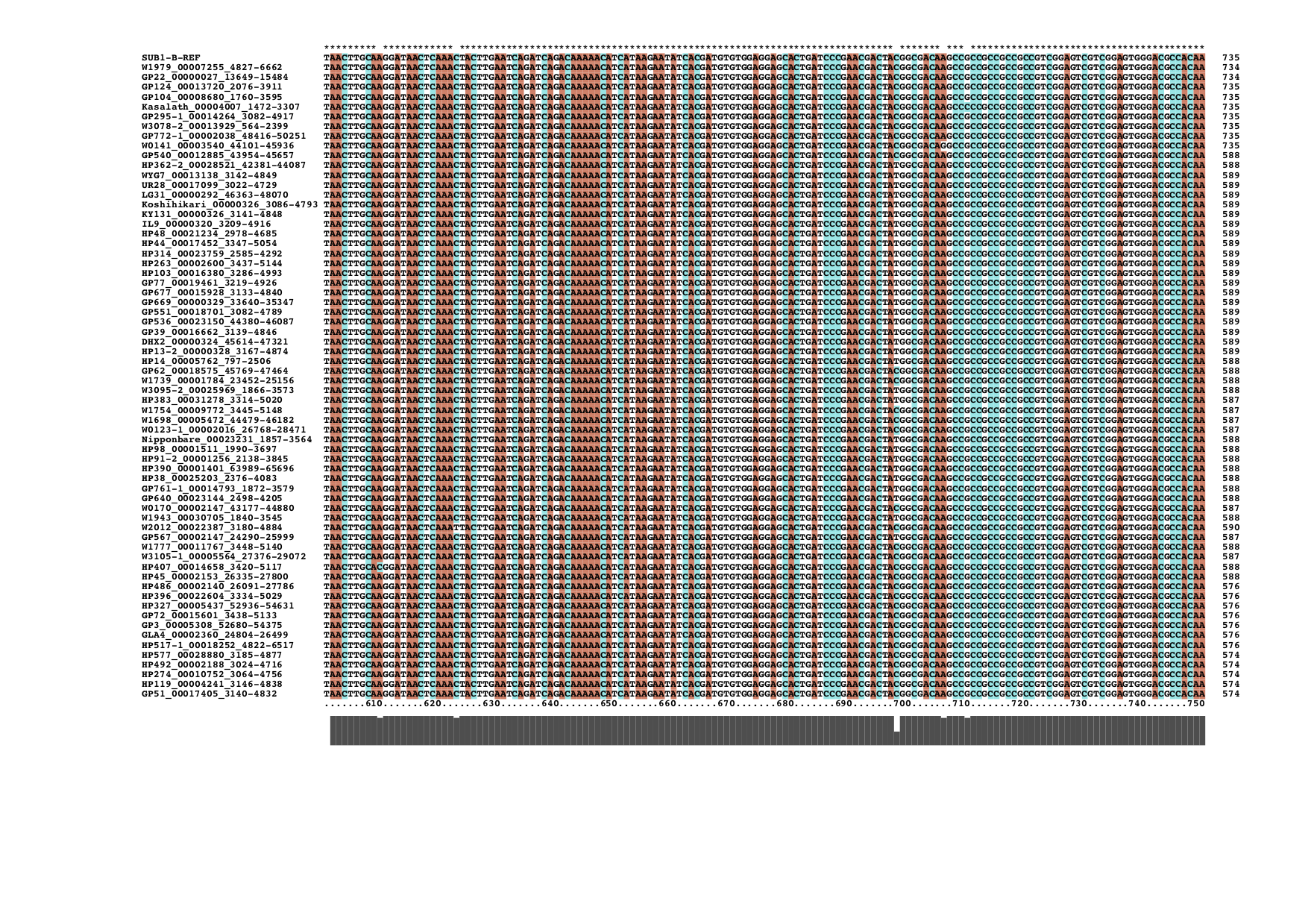


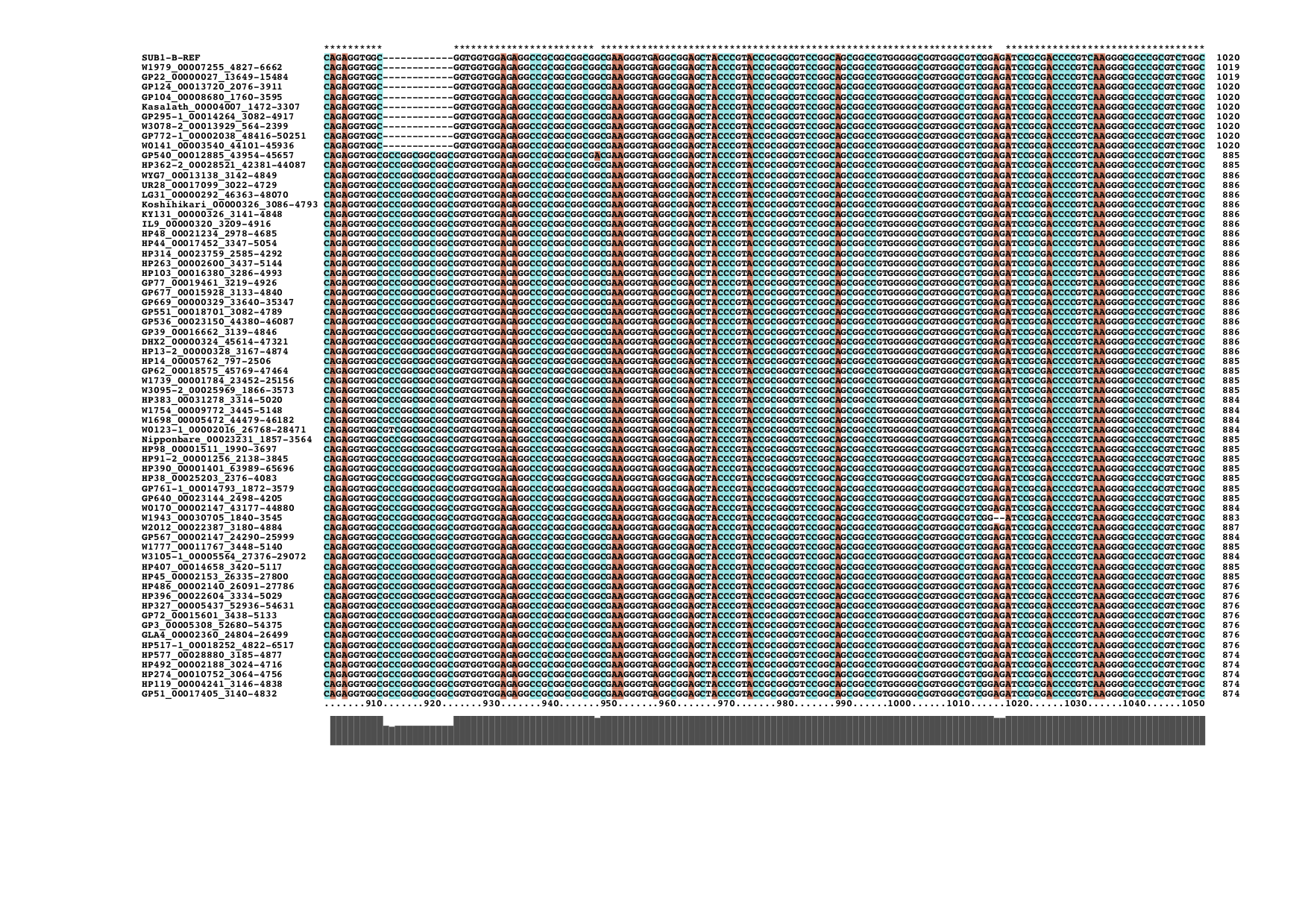


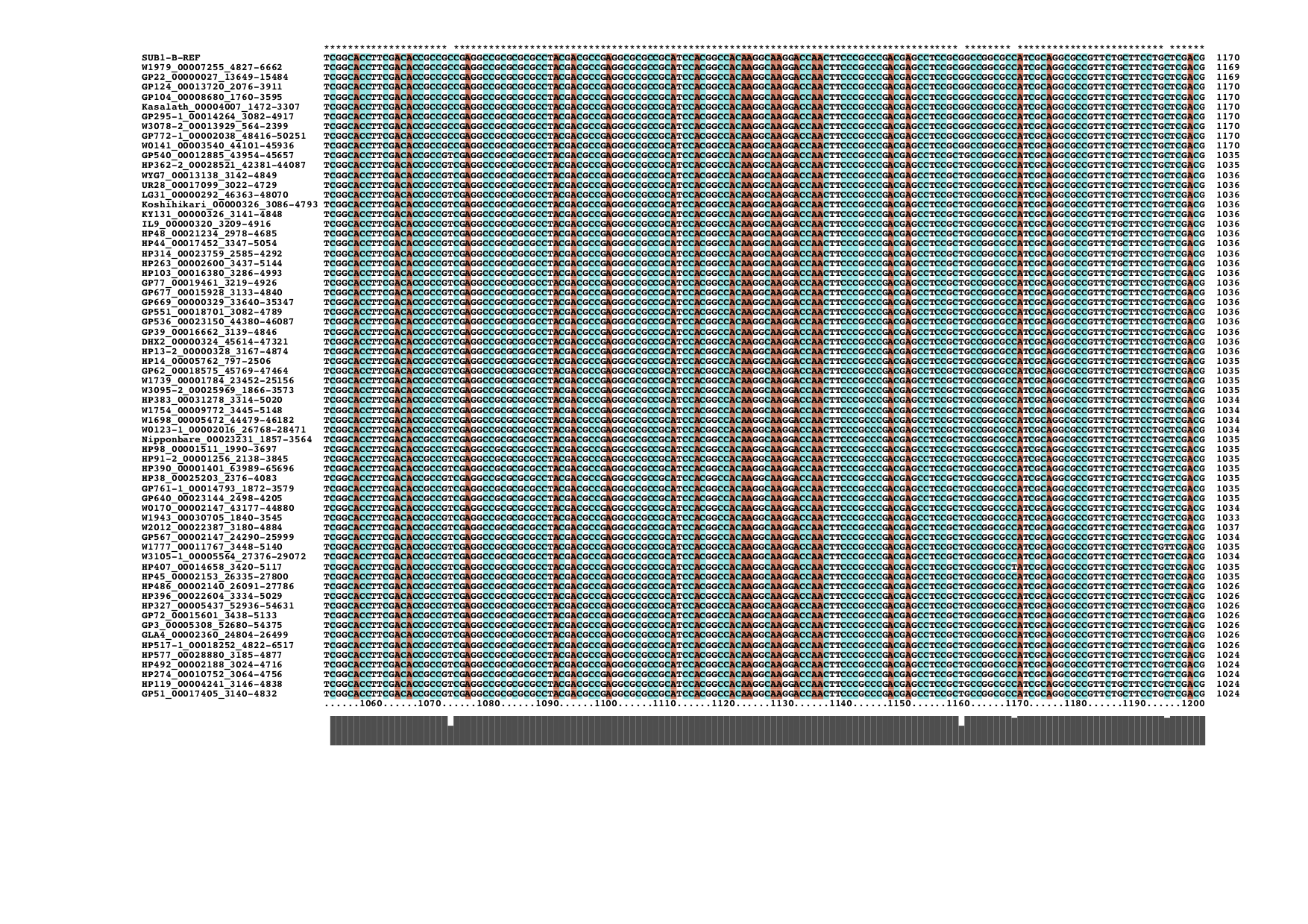

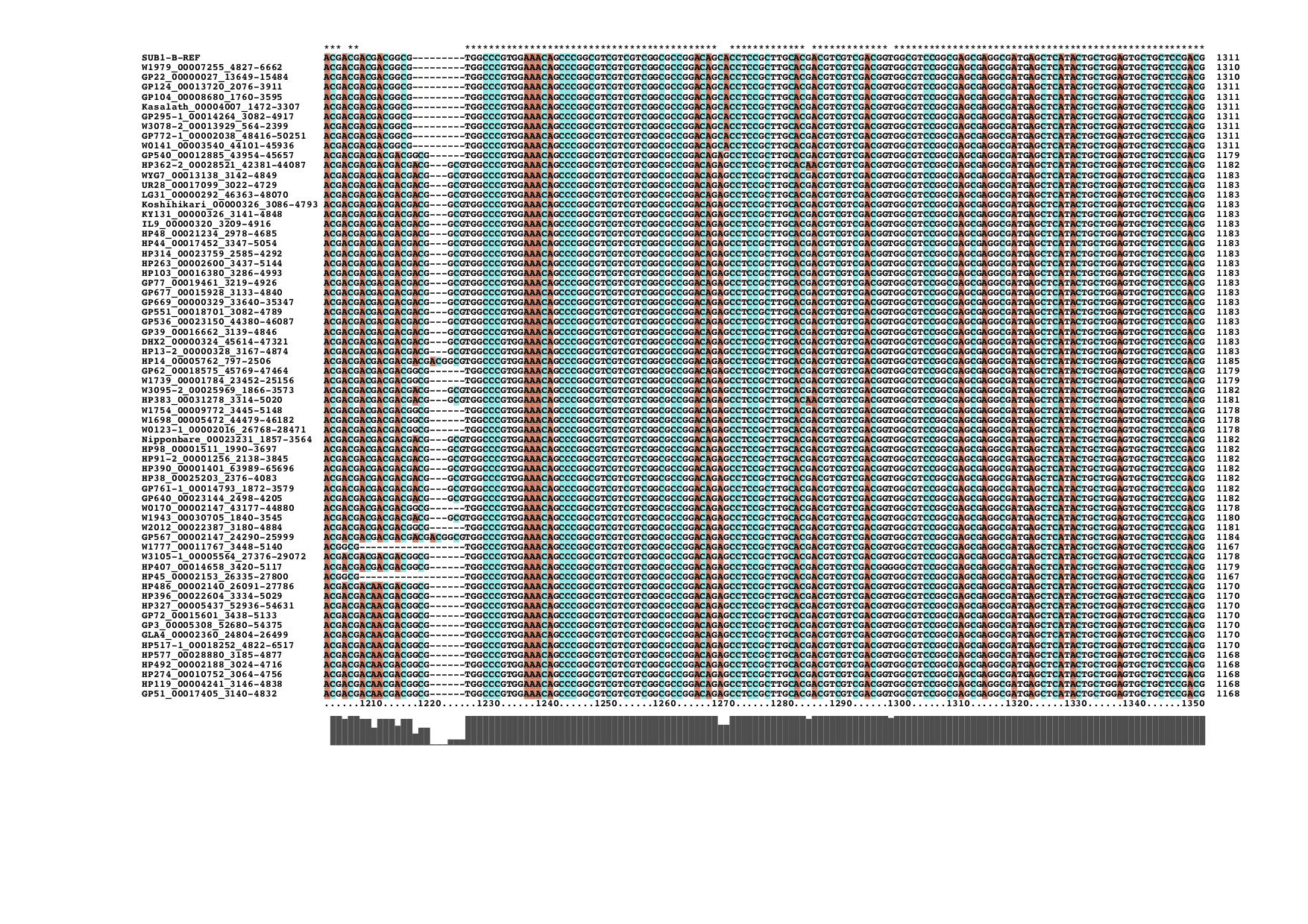


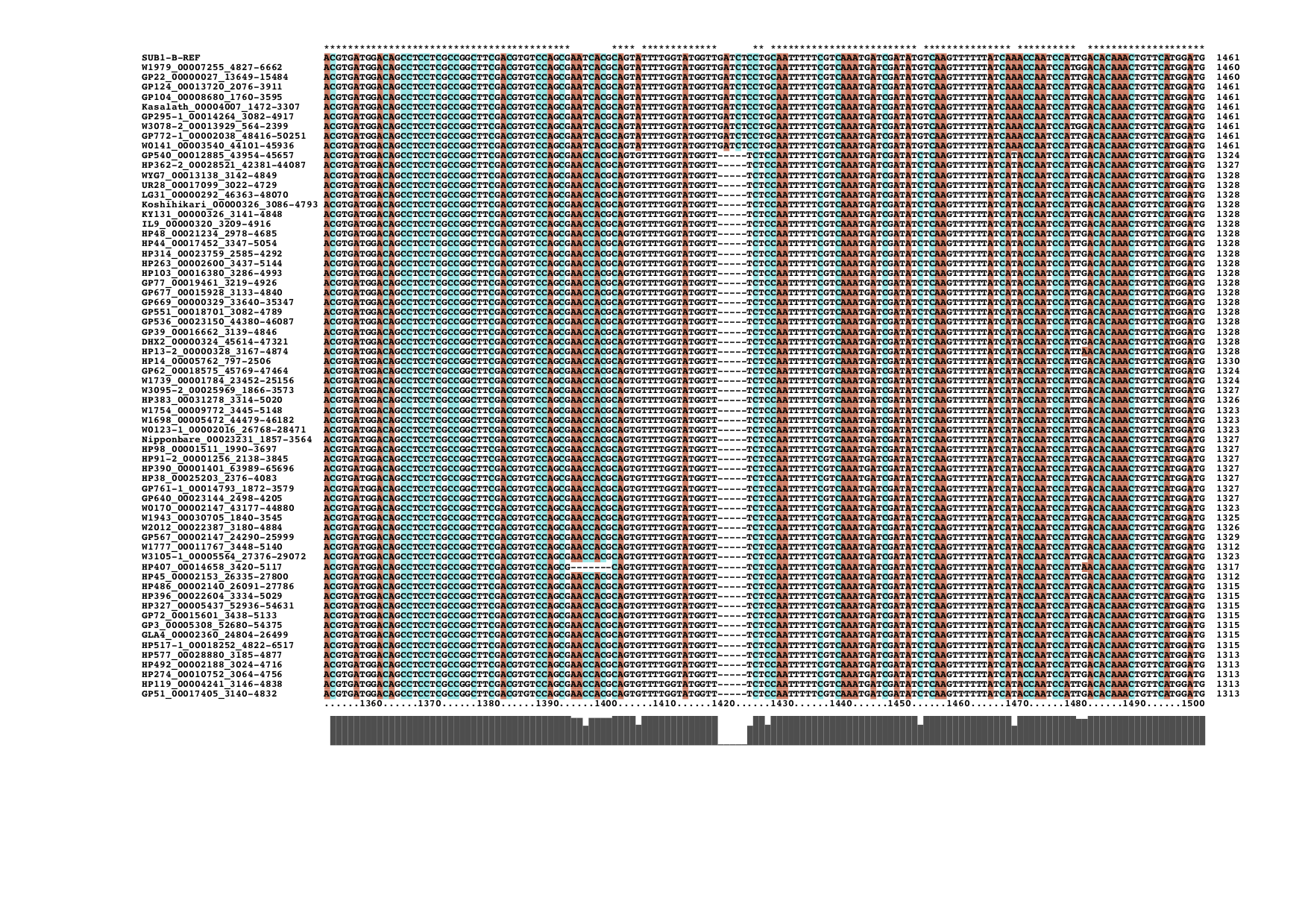


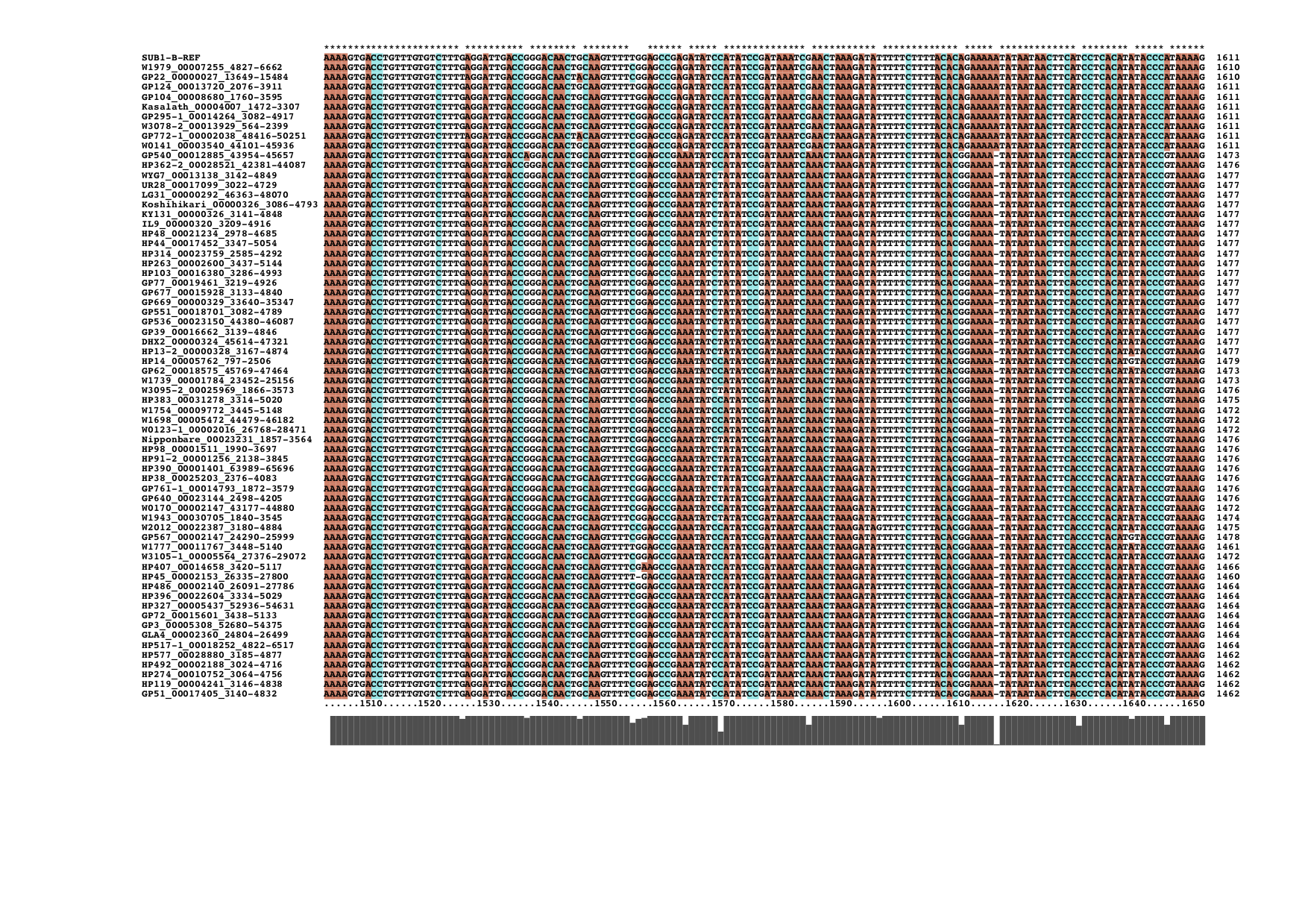


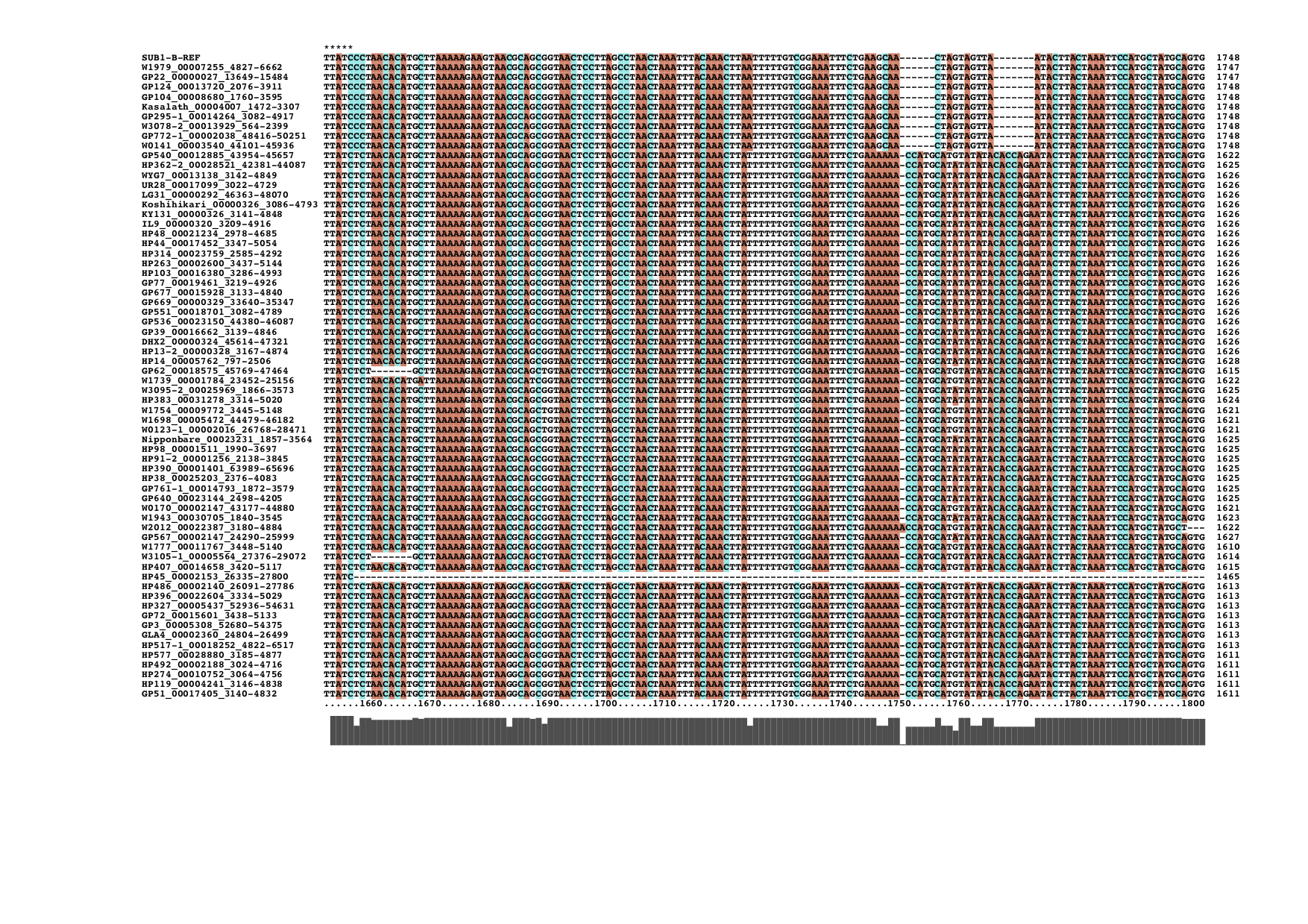


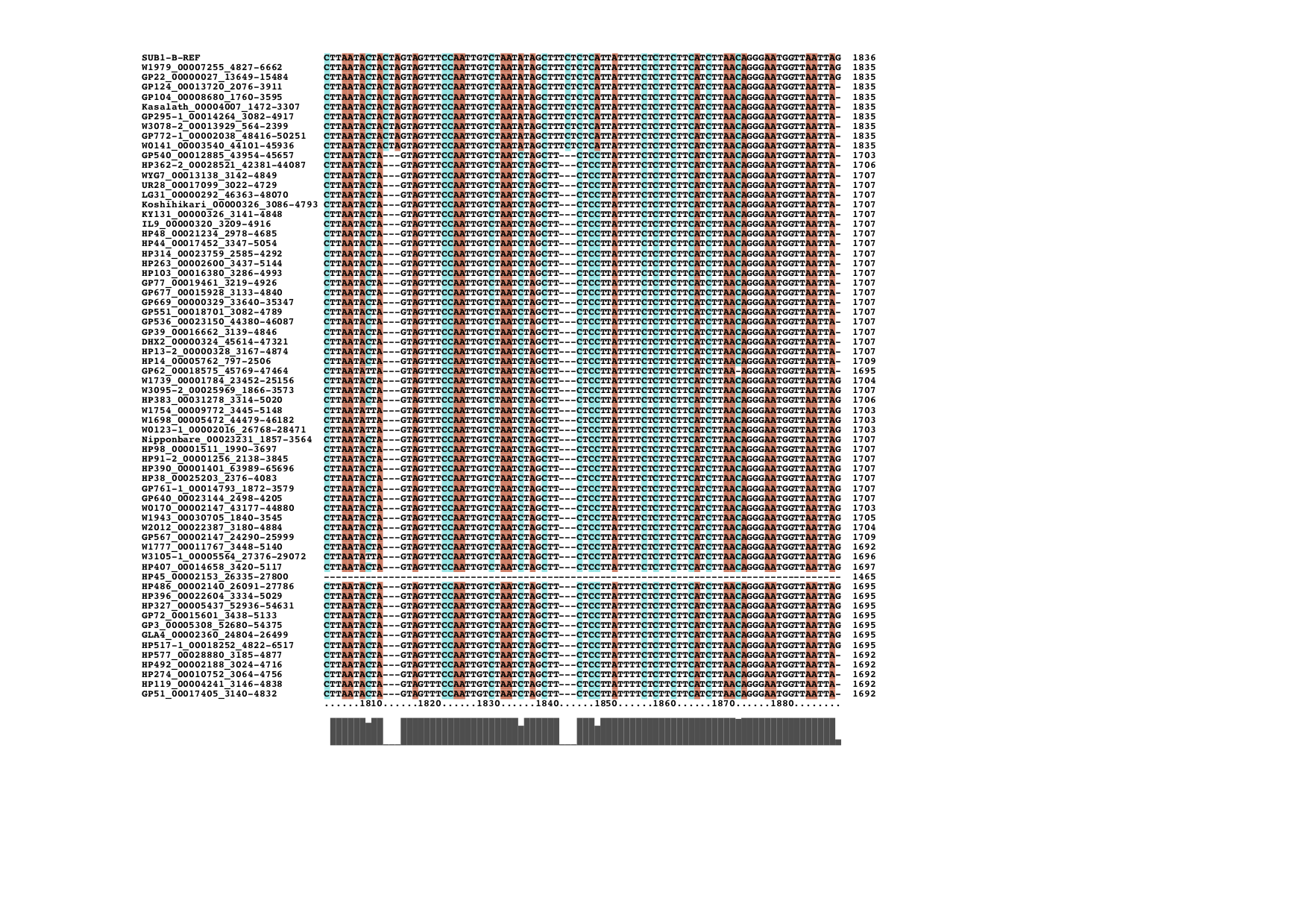


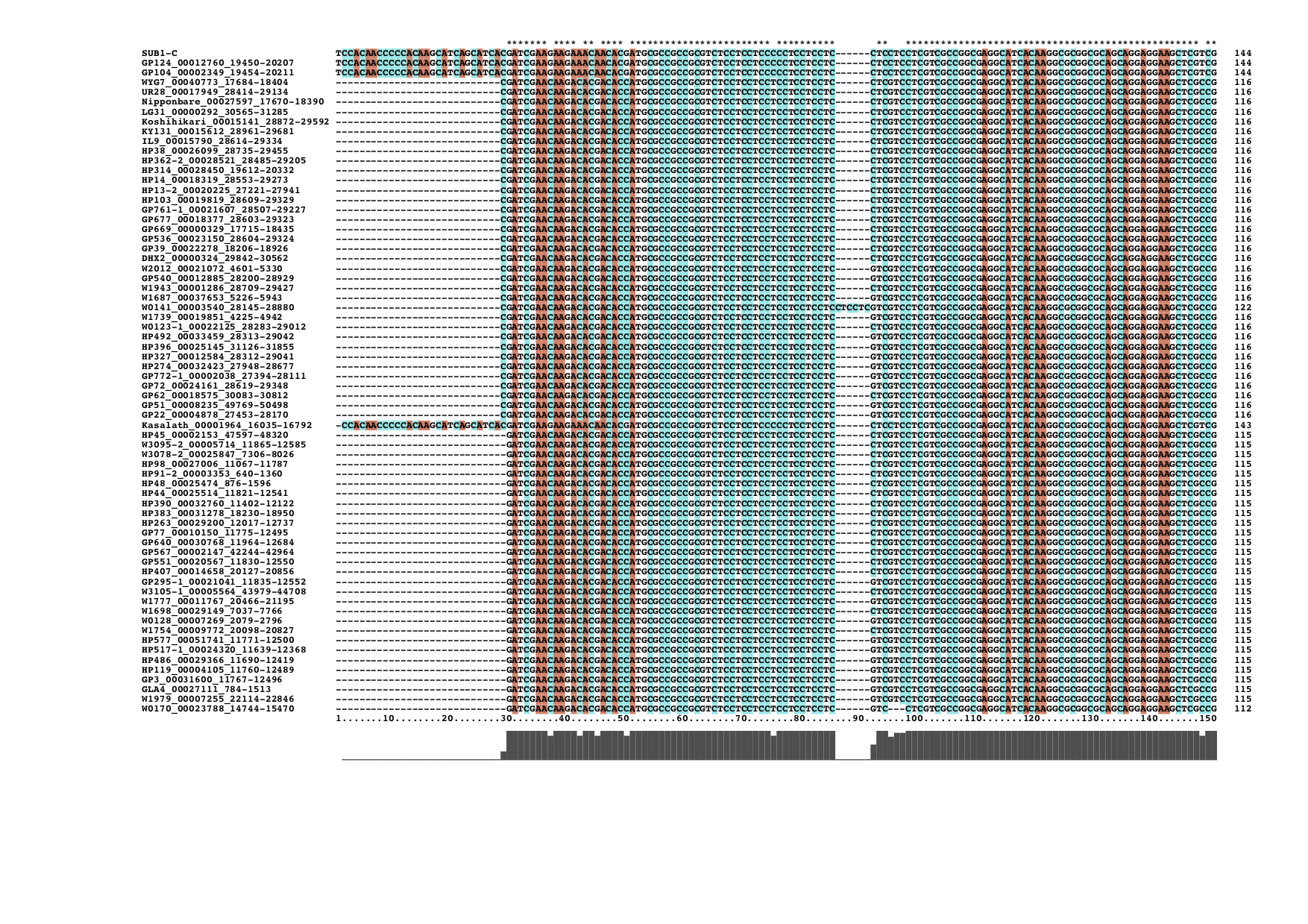


**Figures S7:** Multiple sequence alignment of the *Sub1C* gene in the flood tolerant rice variety FR13A with the sequences present in 66 whole genome sequences of cultivated and wild rice published by Zhao et al. (2018) and the Nipponbare reference genome of rice


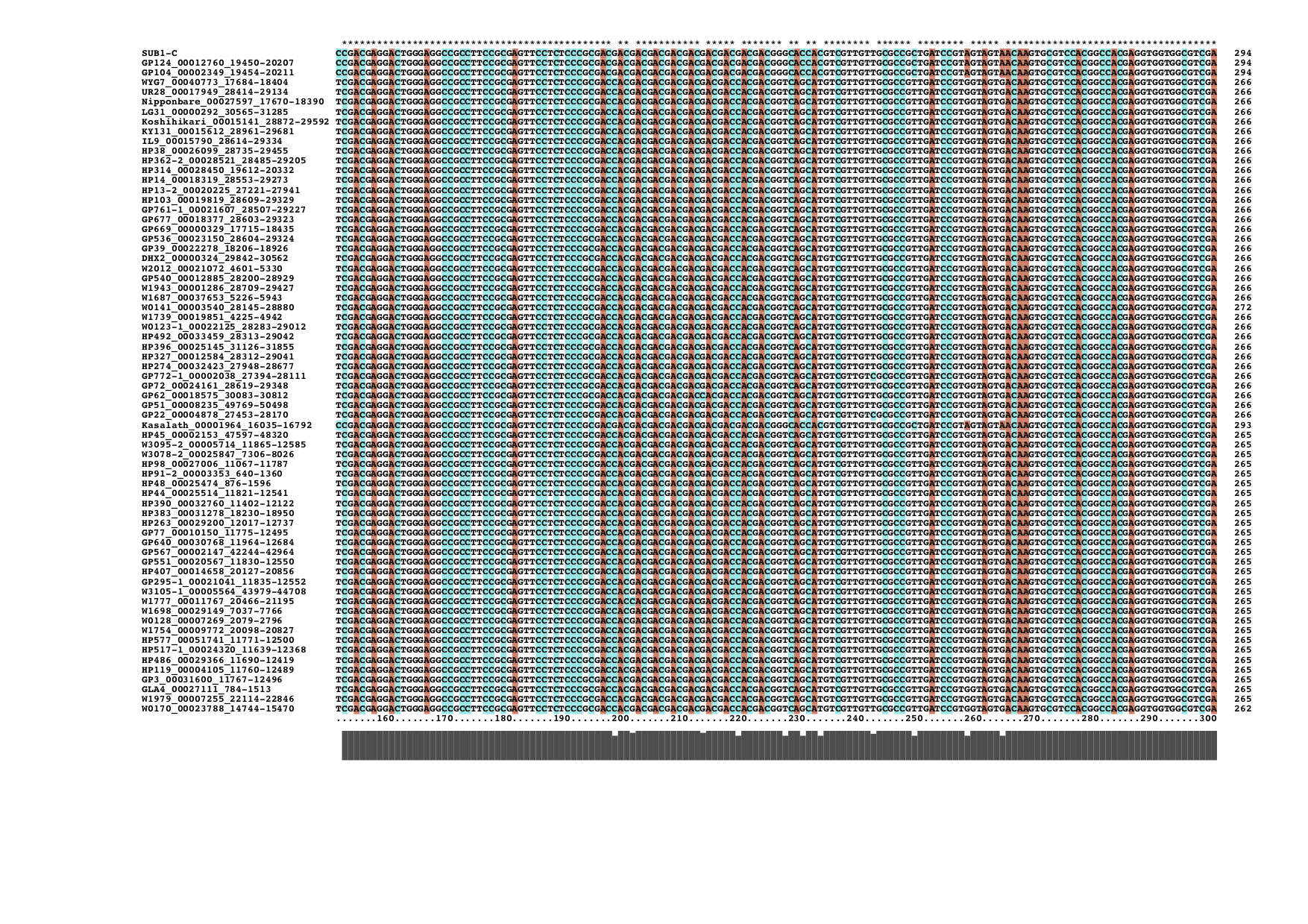


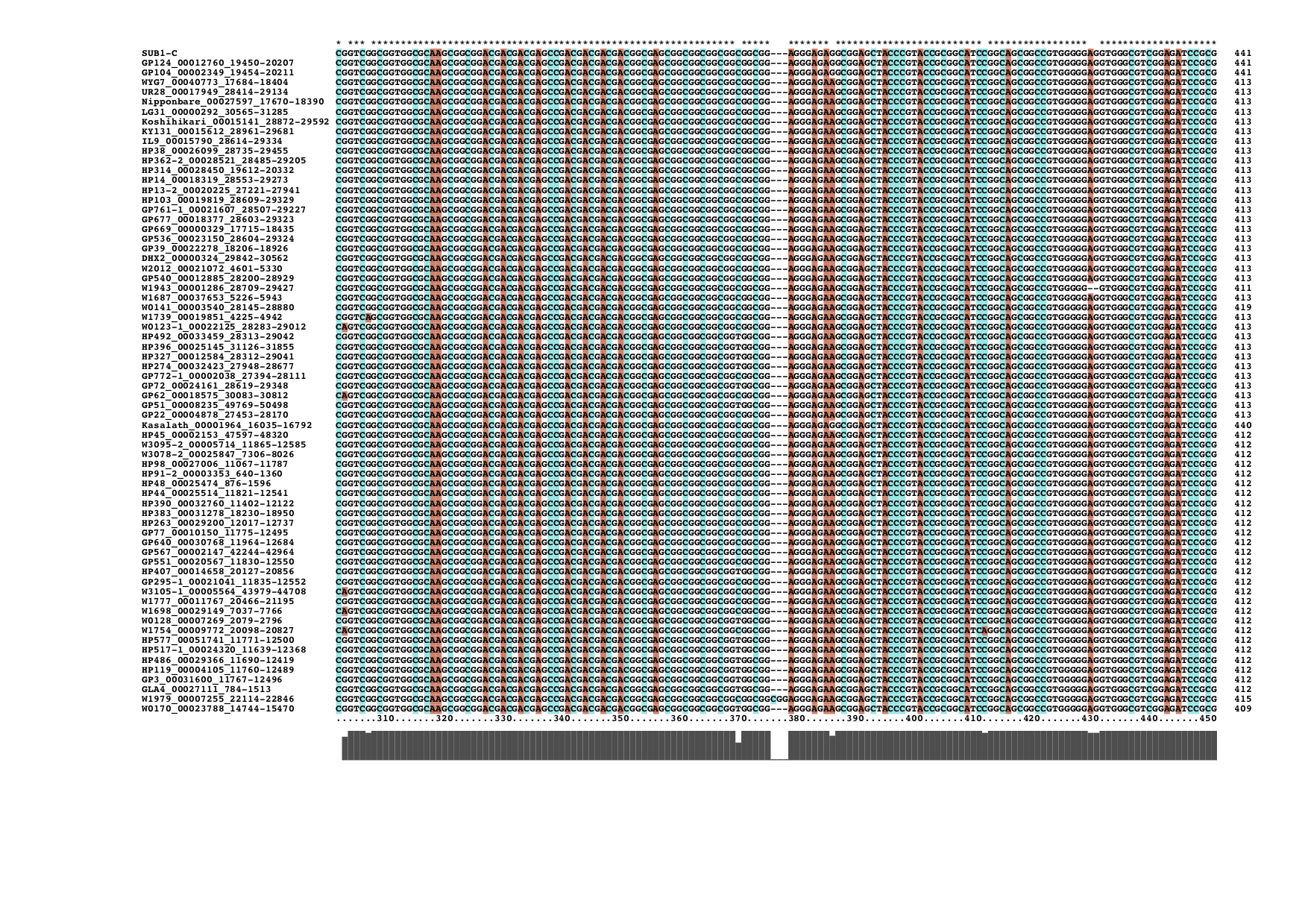


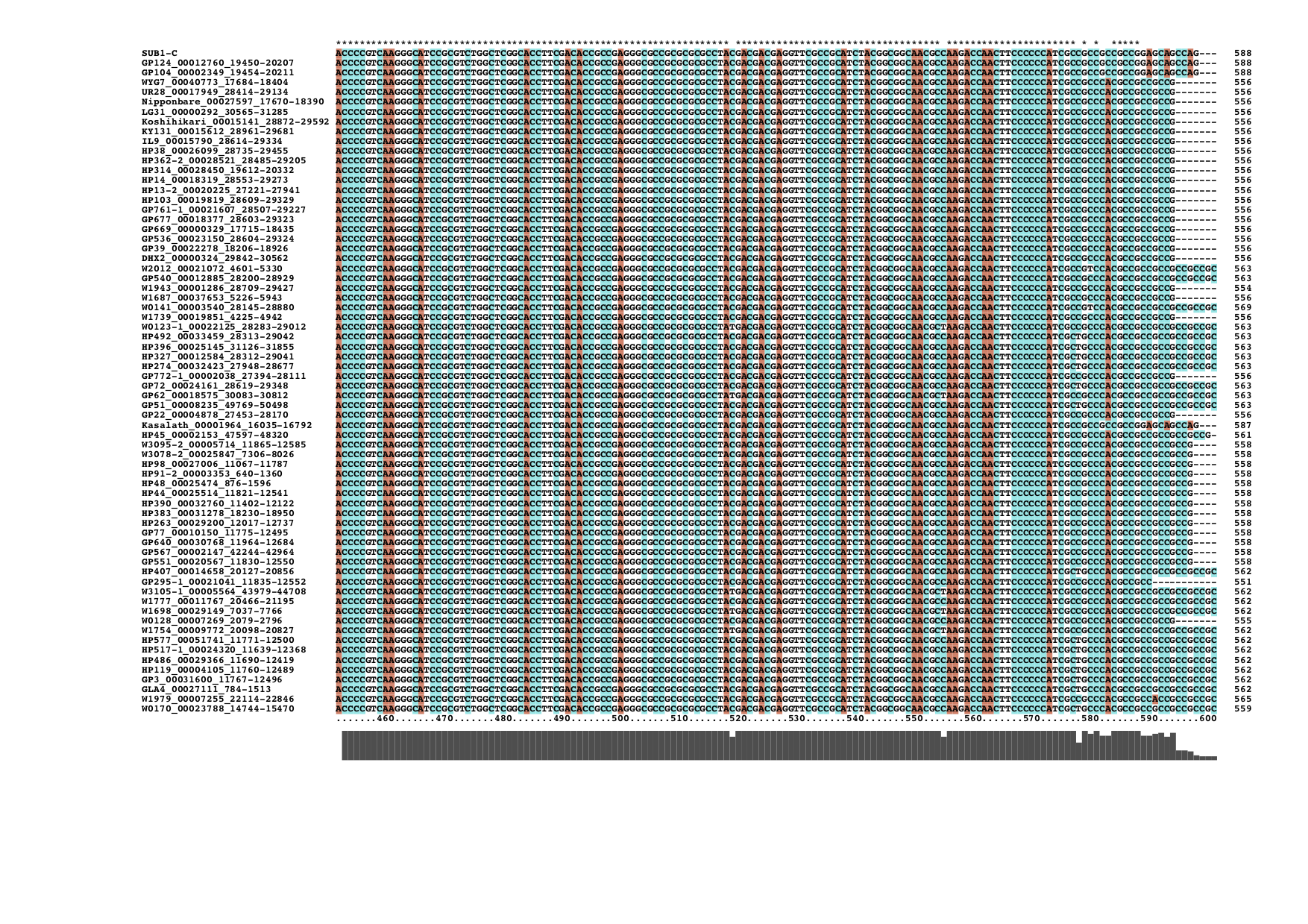


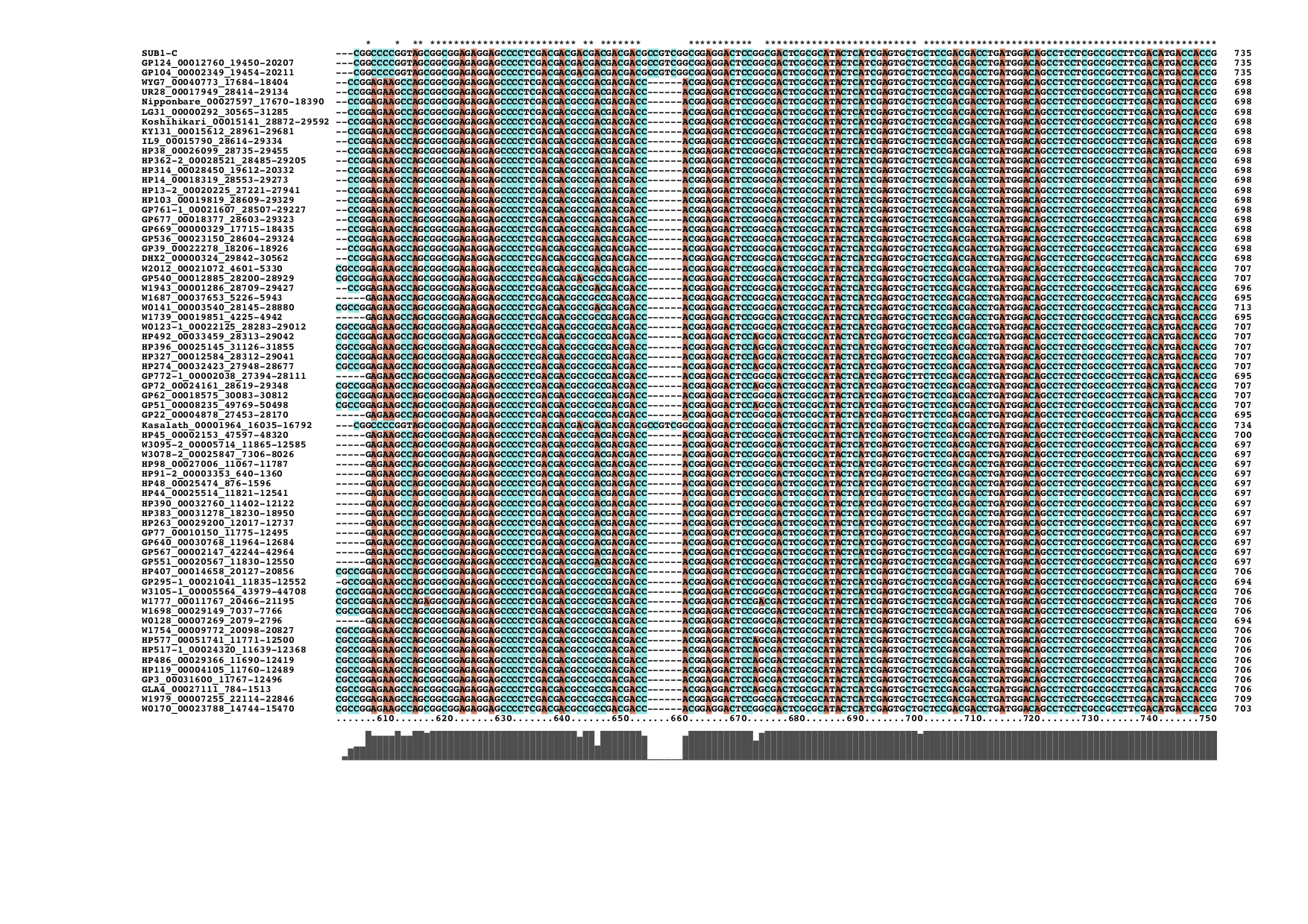


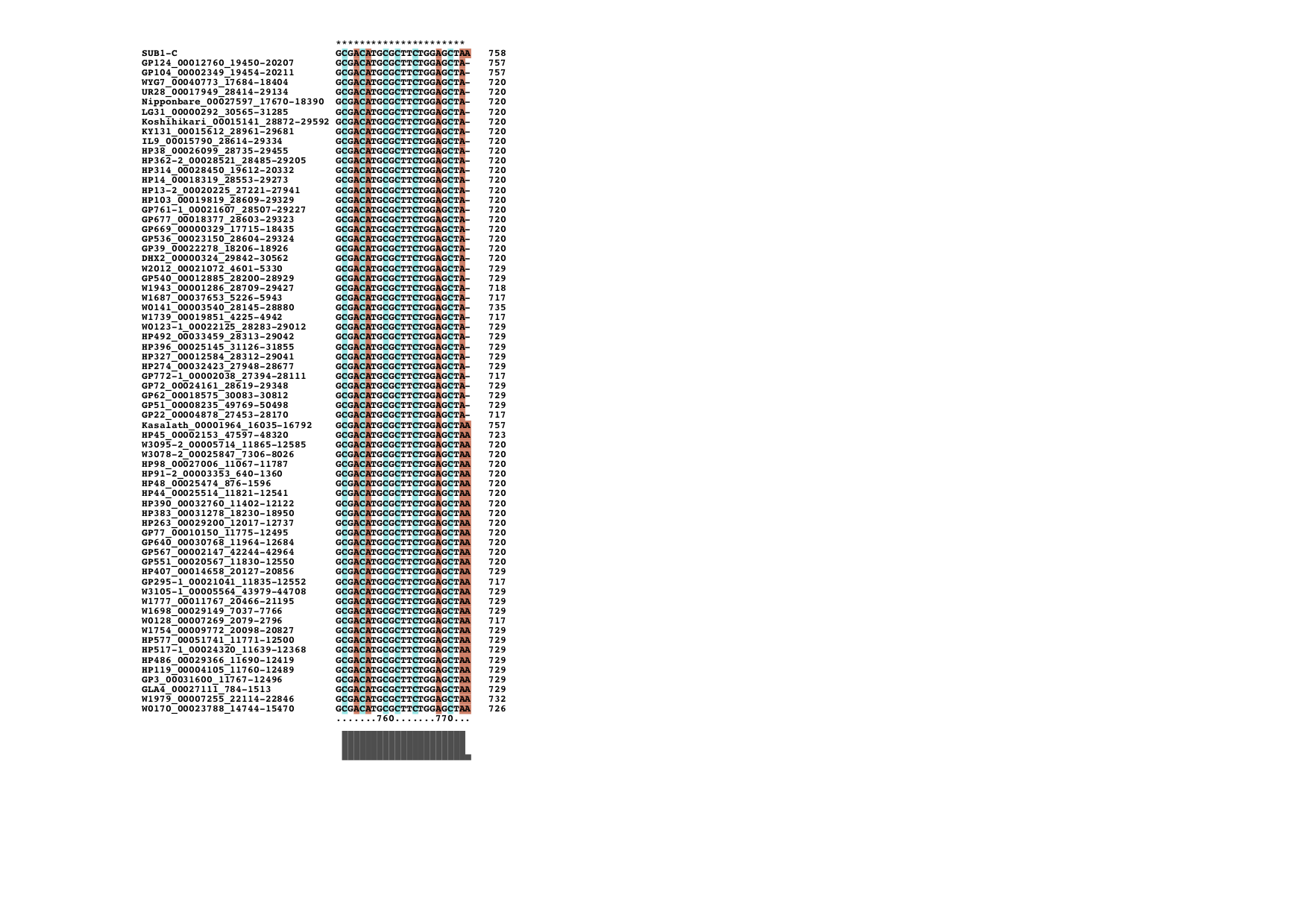


**Table S1.**  Details of 179 varieties and landraces of rice used for allele mining of the *Sub-1A, Sub1B and Sub1C* genes for submergence tolerance by targeted re-sequencing

| **Sr. no.** | **Variety** | **Source** | **Accession no.** | **Origin** | **Biotype** |
| --- | --- | --- | --- | --- | --- |
| 1 | TSAO WAN CHING | IRRI | IRGC 1034 | China | Landrace |
| 2 | AI CHIH AI KWOH 2 | IRRI | IRGC 1272 | China | Landrace |
| 3 | HSUNG TIENG | IRRI | IRGC 1279 | China | NA |
| 4 | DULAR | IRRI | IRGC 3688 | India | Landrace |
| 5 | FR 43B | IRRI | IRGC 6143 | India | Landrace |
| 6 | FR 13A | IRRI | IRGC 6144 | India | Landrace |
| 7 | DHOLAMON 64-3 | IRRI | IRGC 6596 | Bangladesh | Landrace |
| 8 | MAINTIMOLOTSY | IRRI | IRGC 11010 | Madagascar | NA |
| 9 | KURUWEE | IRRI | IRGC 11990 | Sri Lanka | Landrace |
| 10 | MOROBEREKAN | IRRI | IRGC 12048 | Guinea | Landrace |
| 11 | IARI 5829 | IRRI | IRGC 14423 | India | Cultivar |
| 12 | LEMO | IRRI | IRGC 14668 | Indonesia | NA |
| 13 | MADHUKAR | IRRI | IRGC 14781 | India | Cultivar |
| 14 | THUNMAR HAMARA | IRRI | IRGC 15226 | Sri Lanka | NA |
| 15 | KARKATI 87 | IRRI | IRGC 6618 | Bangladesh | NA |
| 16 | JC1 | IRRI | IRGC 9091 | India | NA |
| 17 | POKKALI | IRRI | IRGC 15238 | Sri Lanka | Landrace |
| 18 | KOTTAMALI | IRRI | IRGC 15435 | Sri Lanka | NA |
| 19 | VANAN VELLAI | IRRI | IRGC 15464 | Sri Lanka | NA |
| 20 | NAALUMOLI KARUPPAN | IRRI | IRGC 15478 | Sri Lanka | NA |
| 21 | ARC 12172 | IRRI | IRGC 21960 | India | Landrace |
| 22 | ARC 614 | IRRI | IRGC 24242 | India | Landrace |
| 23 | LABELLE | IRRI | IRGC 24274 | USA | NA |
| 24 | RENIK BATU | IRRI | IRGC 24800 | Indonesia | NA |
| 25 | BIRPALA | IRRI | IRGC 26316 | Bangladesh | NA |
| 26 | BINULAWAN | IRRI | IRGC 26872 | Philippines | NA |
| 27 | JALMAGNA | IRRI | IRGC 26960 | India | NA |
| 28 | AUS 287 | IRRI | IRGC 29075 | Bangladesh | NA |
| 29 | MENYARHUNEI | IRRI | IRGC 30557 | Liberia | NA |
| 30 | PAIAIM | IRRI | IRGC 30578 | Sierra Leone | NA |
| 31 | PALENEMA | IRRI | IRGC 30582 | Sierra Leone | NA |
| 32 | PALIANDEH | IRRI | IRGC 30583 | Sierra Leone | NA |
| 33 | PANNEL | IRRI | IRGC 30588 | Sierra Leone | NA |
| 34 | PANSANKO | IRRI | IRGC 30590 | Sierra Leone | NA |
| 35 | PATAIM | IRRI | IRGC 30591 | Sierra Leone | NA |
| 36 | PAYEMA | IRRI | IRGC 30592 | Sierra Leone | NA |
| 37 | PECALO | IRRI | IRGC 30595 | Sierra Leone | NA |
| 38 | TAGBA | IRRI | IRGC 30625 | Sierra Leone | NA |
| 39 | TATAN | IRRI | IRGC 30627 | Sierra Leone | NA |
| 40 | TIEBIMAH | IRRI | IRGC 30629 | Liberia | NA |
| 41 | GODA HEENATI | IRRI | IRGC 31393 | Sri Lanka | Landrace |
| 42 | KALONCHI | IRRI | IRGC 31836 | Bangladesh | NA |
| 43 | TETEP | IRRI | IRGC 32576 | Vietnam | NA |
| 44 | YEIKOYON | IRRI | IRGC 34470 | Liberia | NA |
| 45 | HEEN SULAI | IRRI | IRGC 36251 | Sri Lanka | NA |
| 46 | JAYMIS | IRRI | IRGC 36259 | Sri Lanka | NA |
| 47 | KALUWEE | IRRI | IRGC 36283 | Sri Lanka | NA |
| 48 | MADAL | IRRI | IRGC 36312 | Sri Lanka | NA |
| 49 | MAHA DIKWEE | IRRI | IRGC 36313 | Sri Lanka | NA |
| 50 | USA BATAPOLA AL | IRRI | IRGC 36397 | Sri Lanka | NA |
| 51 | AUSBORO | IRRI | IRGC 36983 | Bangladesh | NA |
| 52 | JALDUNGI | IRRI | IRGC 37115 | Bangladesh | NA |
| 53 | NIRBOI | IRRI | IRGC 37245 | Bangladesh | NA |
| 54 | PAKRIBUNA | IRRI | IRGC 37259 | Bangladesh | NA |
| 55 | VADAI | IRRI | IRGC 37341 | Bangladesh | NA |
| 56 | GOTA | IRRI | IRGC 37437 | Bangladesh | NA |
| 57 | MARS | IRRI | IRGC 38541 | USA | NA |
| 58 | CR 1009 | IRRI | IRGC 39253 | India | Cultivar |
| 59 | CHAKIA 59 | IRRI | IRGC 40107 | India | NA |
| 60 | FRG1 | IRRI | IRGC 40110 | India | NA |
| 61 | BUNTOK | IRRI | IRGC 43360 | Indonesia | NA |
| 62 | KRETEK SIRENDAH MERAH | IRRI | IRGC 43453 | Indonesia | NA |
| 63 | AZUCENA | IRRI | IRGC 47124 | Philippines | NA |
| 64 | HOURA KANI | IRRI | IRGC 49321 | Bangladesh | NA |
| 65 | NALDAK | IRRI | IRGC 49340 | Bangladesh | NA |
| 66 | BR11 | IRRI | IRGC 53458 | Bangladesh | NA |
| 67 | ZOBGUI | IRRI | IRGC 57675 | Guinea | NA |
| 68 | VAN | IRRI | IRGC 57846 | Guinea | NA |
| 69 | KHAIYAN | IRRI | IRGC 58808 | Bangladesh | Landrace |
| 70 | SITA MATA | IRRI | IRGC 59239 | Nepal | NA |
| 71 | LIU-TIAO-NU | IRRI | IRGC 60183 | China | NA |
| 72 | MA-ZHAN (RED) | IRRI | IRGC 60184 | China | Landrace |
| 73 | SOSSOKA | IRRI | IRGC 61362 | Guinea | NA |
| 74 | MOUSSAYA | IRRI | IRGC 61374 | Guinea | NA |
| 75 | KAOLACK | IRRI | IRGC 61381 | Guinea | NA |
| 76 | NCS 348 | IRRI | IRGC 62256 | India | NA |
| 77 | KAMNAM | IRRI | IRGC 64902 | Bhutan | NA |
| 78 | KARABONKA | IRRI | IRGC 66376 | Guinea | NA |
| 79 | EMPING ARA | IRRI | IRGC 66545 | Indonesia | NA |
| 80 | ROM RUNTIK | IRRI | IRGC 66627 | Indonesia | Landrace |
| 81 | LEMONT | IRRI | IRGC 66756 | USA | Landrace |
| 82 | LATSIKA | IRRI | IRGC 69367 | Madagascar | NA |
| 83 | SANG KHLA | IRRI | IRGC 73597 | Thailand | NA |
| 84 | SANTI BAHN | IRRI | IRGC 73598 | Thailand | NA |
| 85 | TAH TONE | IRRI | IRGC 73618 | Thailand | NA |
| 86 | WAHNG | IRRI | IRGC 73625 | Thailand | NA |
| 87 | SIPULUT PANDAN | IRRI | IRGC 73840 | Indonesia | NA |
| 88 | AKITAKOMACHI | IRRI | IRGC 76312 | Japan | NA |
| 89 | KATY | IRRI | IRGC 77140 | USA | NA |
| 90 | M202 | IRRI | IRGC 77142 | USA | Cultivar |
| 91 | KARNAL LOCAL | IRRI | IRGC 78484 | India | Cultivar |
| 92 | KHAO HLAN ON | IRRI | IRGC 88335 | Myanmar | Landrace |
| 93 | KHAO MINE LAR | IRRI | IRGC 88336 | Myanmar | Landrace |
| 94 | IR 64 | IRRI | IRGC 116793 | Philippines | Cultivar |
| 95 | SWARNA | IRRI | IRGC 117278 | India | Cultivar |
| 96 | KHARSU 80A | IRRI | IRGC 28017 | Pakistan | Landrace |
| 97 | KASALATH | IRRI | IRTP 23786 | India | Landrace |
| 98 | IR 72 | IRRI | IRTP 14747 | Philippines | Cultivar |
| 99 | IR 42 | IRRI | IRTP 1180 | Philippines | Cultivar |
| 100 | IR-73707-45-3-23 | IRRI | IRTP 22709 | Philippines | Cultivar |
| 101 | BARA SALI | NBPGR | IC 466475 | India | Landrace |
| 102 | IR 36 | NBPGR | NA | Philippines | Cultivar |
| 103 | IC 370811 | NBPGR | IC 370811 | India | Landrace |
| 104 | CHANGMAKA | NBPGR | IC 466868 | India | Landrace |
| 105 | CHOW HA LA | NBPGR | IC 46388 | India | Landrace |
| 106 | CHANGMAN | NBPGR | IC 464211 | India | Landrace |
| 107 | BABARAKSHA KARO | NBPGR | IC 258839 | India | Landrace |
| 108 | JOHAINEANG (WET ) | NBPGR | IC 465024 | India | Landrace |
| 109 | BARACHIRAMARA | NBPGR | IC 467236 | India | Landrace |
| 110 | ASAMIRUPA | NBPGR | IC 256743 | India | Landrace |
| 111 | CHUKKANNA KUMBALOAN | NBPGR | IC 449878 | India | Landrace |
| 112 | CHULTA BALUNGA (WILD) | NBPGR | IC 449938 | India | Landrace |
| 113 | CHANGPHAI | NBPGR | IC 467119 | India | Landrace |
| 114 | CHASING | NBPGR | IC 465039 | India | Landrace |
| 115 | CHING CHANG | NBPGR | IC 467191 | India | Landrace |
| 116 | MEGHI | NBPGR | IC 466795 | India | Landrace |
| 117 | ASHRA LAKI | NBPGR | IC 467190 | India | Landrace |
| 118 | BAGA IKRA | NBPGR | IC 464987 | India | Landrace |
| 119 | JOKHRU | NBPGR | IC 465058 | India | Landrace |
| 120 | CHOWMAI | NBPGR | IC 466626 | India | Landrace |
| 121 | CHANGNGAT | NBPGR | IC 464275 | India | Landrace |
| 122 | PANIKAKUA | NBPGR | IC 460019 | India | Landrace |
| 123 | CHUMANSAL-39 | NBPGR | IC 450313 | India | Landrace |
| 124 | JAHABHOG | NBPGR | IC 466483 | India | Landrace |
| 125 | BAD KODI | NBPGR | IC 464217 | India | Landrace |
| 126 | CHANG MUI | NBPGR | IC 466695 | India | Landrace |
| 127 | DUB GELONG | NBPGR | IC 463907 | India | Landrace |
| 128 | PANIDUBI | NBPGR | IC 256674 | India | Landrace |
| 129 | MEGHARAJ | NBPGR | IC 463682 | India | Landrace |
| 130 | ASAMIA | NBPGR | IC 256711 | India | Landrace |
| 131 | DHUSARI AHU | NBPGR | IC 466886 | India | Landrace |
| 132 | BAGA GOHA | NBPGR | IC 466800 | India | Landrace |
| 133 | BARA RANGA | NBPGR | IC 466776 | India | Landrace |
| 134 | KHAWJII | NBPGR | IC 466690 | India | Landrace |
| 135 | JAGANATHA BALLAVA | NBPGR | IC 283036 | India | Landrace |
| 136 | JALKANTHI | NBPGR | IC 256848 | India | Landrace |
| 137 | CHUDI | CRRI | IC 426114 | India | Landrace |
| 138 | MANOHAR SALI | CRRI | IC 463188 | India | Cultivar |
| 139 | BABAI LACHHA | CRRI | IC 380275 | India | Landrace |
| 140 | LUNISHREE | CRRI | IC 443777 | India | Cultivar |
| 141 | MANDIRA | CRRI | IC 443941 | India | Cultivar |
| 142 | ASANA | CRRI | IC 277229 | India | Landrace |
| 143 | PANI DHAN 2 | CRRI | IC 454295 | India | Landrace |
| 144 | TULASI | CRRI | Tulasi | India | Cultivar |
| 145 | SAVITRI | CRRI | IC 75525 | India | Cultivar |
| 146 | SWARNA-SUB1 | IARI | Swarna-Sub1 | India | Cultivar |
| 147 | BAD DHANA | CRRI | IC 380615 | India | Landrace |
| 148 | ASHU BHAJNA | CRRI | IC 380272 | India | Landrace |
| 149 | BAD GEDE | CRRI | IC 380288 | India | Landrace |
| 150 | PADMA | CRRI | IC 512927 | India | Landrace |
| 151 | PANIKEKEUA | CRRI | IC 380486 | India | Landrace |
| 152 | PANKEI | CRRI | IC 380260 | India | Landrace |
| 153 | BAD JHARLI | CRRI | IC 380289 | India | Landrace |
| 154 | S-183 | CRRI | CRRI-S-183 | India | Landrace |
| 155 | SAMBHA-SUB1 | NDUAT | Sambha-Sub1 | India | Cultivar |
| 156 | JALNIDHI | NDUAT | Jalnidhi | India | Cultivar |
| 157 | GOTHAWA | NRCPB | NKSLR-1 | India | Landrace |
| 158 | KAJARAHAWA | NRCPB | NKSLR-2 | India | Landrace |
| 159 | SATHWA | NRCPB | NKSLR-3 | India | Landrace |
| 160 | RAJBHOG | NRCPB | NKSLR-4 | India | Landrace |
| 161 | SINGHARA | NRCPB | NKSLR-5 | India | Landrace |
| 162 | TUNDAHIYA | NRCPB | NKSLR-6 | India | Landrace |
| 163 | SURAHA | NRCPB | NKSLR-8 | India | Landrace |
| 164 | KARIYAWA | NRCPB | NKSLR-10 | India | Landrace |
| 165 | DIHAWAN | NRCPB | NKSLR-11 | India | Landrace |
| 166 | MOTI | NRCPB | NKSLR-13 | India | Landrace |
| 167 | SUGAPANKH | NRCPB | NKSLR-14 | India | Landrace |
| 168 | JADAHAN | NRCPB | NKSLR-15 | India | Landrace |
| 169 | DUDHA LADU | NRCPB | NKSLR-17 | India | Landrace |
| 170 | S-150 | CRRI | CRRI-S-150 | India | Landrace |
| 171 | S-172 | CRRI | CRRI-S-172 | India | Landrace |
| 172 | S-148 | CRRI | CRRI-S-148 | India | Landrace |
| 173 | S-177 | CRRI | CRRI-S-177 | India | Landrace |
| 174 | S-138 | CRRI | CRRI-S-138 | India | Landrace |
| 175 | S-141 | CRRI | CRRI-S-141 | India | Landrace |
| 176 | S-155 | CRRI | CRRI-S-155 | India | Landrace |
| 177 | MEGHA BADALA | NBPGR | IC 2556616 | India | Landrace |
| 178 | KALA BUNDE | NBPGR | IC 420062 | India | Landrace |
| 179 | KOLA JOHA | NBPGR | IC 558250 | India | Landrace |

**Table S2.** Inferred sub-population Structures of 179 genotypes under study based on genotyping using 36 genome-wide unlinked SNPs. Serial numbers correspond to population structure bar plot in Fig. 1A. Green and red shadings are for Indica and Japonica-Aromatic cultivars, respectively. Lighter color shades are for 18 cultivars having Fst values of <0.800.

| **S. no.** | **VARIETY** | **Fst1** | **Fst2** | **Population** | **S. no.** | **VARIETY** | **Fst1** | **Fst2** | **Population** |
| --- | --- | --- | --- | --- | --- | --- | --- | --- | --- |
| 1 | TSAO WAN CHIN | 0.038 | 0.962 | 2 | 41 | GODAHEENATI | 0.022 | 0.978 | 2 |
| 2 | AICHIHAIKWO | 0.002 | 0.998 | 2 | 42 | KALONCHI | 0.386 | 0.614 | 2 |
| 3 | HSUNGTIENG | 0.002 | 0.998 | 2 | 43 | TETEP | 0.054 | 0.946 | 2 |
| 4 | DULAR | 0.323 | 0.677 | 2 | 44 | YEIKOYON | 0.057 | 0.943 | 2 |
| 5 | FR43B | 0.005 | 0.995 | 2 | 45 | HEENSULAI | 0.122 | 0.878 | 2 |
| 6 | FR13A | 0.477 | 0.523 | 2 | 46 | JAYMIS | 0.008 | 0.992 | 2 |
| 7 | DHOLAMON643 | 0.011 | 0.989 | 2 | 47 | KALUWEE | 0.006 | 0.994 | 2 |
| 8 | MAINTIMOLOT | 0.997 | 0.003 | 1 | 48 | MADAL | 0.009 | 0.991 | 2 |
| 9 | KURUWEE | 0.015 | 0.985 | 2 | 49 | MAHADIKWEE | 0.009 | 0.991 | 2 |
| 10 | MOROBEREKAN | 0.997 | 0.003 | 1 | 50 | USABATAPOLA | 0.004 | 0.996 | 2 |
| 11 | IARI5829 | 0.005 | 0.995 | 2 | 51 | AUSBORO | 0.546 | 0.454 | 1 |
| 12 | LEMO | 0.027 | 0.973 | 2 | 52 | JALDUNGI | 0.005 | 0.995 | 2 |
| 13 | MADHUKAR | 0.012 | 0.988 | 2 | 53 | NIRBOI | 0.006 | 0.994 | 2 |
| 14 | THUNMARHAMA | 0.009 | 0.991 | 2 | 54 | PAKRIBUNA | 0.013 | 0.987 | 2 |
| 15 | KARKATI87 | 0.005 | 0.995 | 2 | 55 | VADAI | 0.004 | 0.996 | 2 |
| 16 | JC 1 | 0.989 | 0.011 | 1 | 56 | GOTA | 0.025 | 0.975 | 2 |
| 17 | POKKALI | 0.006 | 0.994 | 2 | 57 | MARS | 0.997 | 0.003 | 1 |
| 18 | KOTTAMALI | 0.003 | 0.997 | 2 | 58 | CR1009 | 0.004 | 0.996 | 2 |
| 19 | VANANVELLAI | 0.024 | 0.976 | 2 | 59 | CHAKIA59 | 0.006 | 0.994 | 2 |
| 20 | NAALUMOLIKA | 0.011 | 0.989 | 2 | 60 | FRG1 | 0.003 | 0.997 | 2 |
| 21 | ARC 12172 | 0.385 | 0.615 | 2 | 61 | BUNTOK | 0.006 | 0.994 | 2 |
| 22 | ARC614 | 0.029 | 0.971 | 2 | 62 | KRETEKSIREN | 0.044 | 0.956 | 2 |
| 23 | LABELLE | 0.997 | 0.003 | 1 | 63 | AZUCENA | 0.996 | 0.004 | 1 |
| 24 | RENIKBATU | 0.154 | 0.846 | 2 | 64 | HOURAKANI | 0.091 | 0.909 | 2 |
| 25 | BIRPALA | 0.008 | 0.992 | 2 | 65 | NALDAK | 0.041 | 0.959 | 2 |
| 26 | BINULAWAN | 0.997 | 0.003 | 1 | 66 | BR11 | 0.005 | 0.995 | 2 |
| 27 | JALMAGNA | 0.663 | 0.337 | 1 | 67 | ZOBGUI | 0.101 | 0.899 | 2 |
| 28 | AUS 287 | 0.661 | 0.339 | 1 | 68 | VAN | 0.007 | 0.993 | 2 |
| 29 | MENYARHUNEI | 0.025 | 0.975 | 2 | 69 | KHAIYAN | 0.291 | 0.709 | 2 |
| 30 | PAIAIM | 0.023 | 0.977 | 2 | 70 | SITAMATA | 0.005 | 0.995 | 2 |
| 31 | PALENEMA | 0.005 | 0.995 | 2 | 71 | LIU-TIAO-NU | 0.004 | 0.996 | 2 |
| 32 | PALIANDEH | 0.008 | 0.992 | 2 | 72 | MAZHAN(RED) | 0.005 | 0.995 | 2 |
| 33 | PANNEL | 0.006 | 0.994 | 2 | 73 | SOSSOKA | 0.007 | 0.993 | 2 |
| 34 | PANSANKO | 0.003 | 0.997 | 2 | 74 | MOUSSAYA | 0.020 | 0.980 | 2 |
| 35 | PATAIM | 0.003 | 0.997 | 2 | 75 | KAOLACK | 0.023 | 0.977 | 2 |
| 36 | PAYEMA | 0.004 | 0.996 | 2 | 76 | NCS 348 | 0.407 | 0.593 | 2 |
| 37 | PECALO | 0.05 | 0.95 | 2 | 77 | KAMNAM | 0.970 | 0.030 | 1 |
| 38 | TAGBA | 0.024 | 0.976 | 2 | 78 | KARABONKA | 0.003 | 0.997 | 2 |
| 39 | TATAN | 0.003 | 0.997 | 2 | 79 | EMPINGARA | 0.041 | 0.959 | 2 |
| 40 | TIEBIMAH | 0.003 | 0.997 | 2 | 80 | ROMRUNTIK | 0.011 | 0.989 | 2 |
| 81 | LEMONT | 0.997 | 0.003 | 1 | 124 | JAHABHOG | 0.996 | 0.004 | 1 |
| 82 | LATSIKA | 0.854 | 0.146 | 1 | 125 | BADKODI | 0.997 | 0.003 | 1 |
| 83 | SANGKHLA | 0.007 | 0.993 | 2 | 126 | CHANGMUI | 0.007 | 0.993 | 2 |
| 84 | SANTIBAHN | 0.002 | 0.998 | 2 | 127 | DUBGELONG | 0.264 | 0.736 | 2 |
| 85 | TAHTONE | 0.009 | 0.991 | 2 | 128 | PANIDUBI | 0.005 | 0.995 | 2 |
| 86 | WAHNG | 0.007 | 0.993 | 2 | 129 | MEGHRAJ | 0.998 | 0.002 | 1 |
| 87 | SIPULUTPAND | 0.015 | 0.985 | 2 | 130 | ASAMIA | 0.008 | 0.992 | 2 |
| 88 | AKITAKOMACH | 0.998 | 0.002 | 1 | 131 | DHUSARIAHU | 0.045 | 0.955 | 2 |
| 89 | KATY | 0.972 | 0.028 | 1 | 132 | BAGAGOHA | 0.990 | 0.01 | 1 |
| 90 | M202 | 0.997 | 0.003 | 1 | 133 | BARARANGA | 0.019 | 0.981 | 2 |
| 91 | KARNAL LOCAL | 0.993 | 0.007 | 1 | 134 | KHAWJII | 0.002 | 0.998 | 2 |
| 92 | KHAOHLANON | 0.019 | 0.981 | 2 | 135 | JAGANATHABA | 0.004 | 0.996 | 2 |
| 93 | KHAOMINELAR | 0.003 | 0.997 | 2 | 136 | JALKANTHI | 0.003 | 0.997 | 2 |
| 94 | IR64 | 0.072 | 0.928 | 2 | 137 | CHUDI | 0.008 | 0.992 | 2 |
| 95 | SWARNA | 0.004 | 0.996 | 2 | 138 | MANOHARSALI | 0.007 | 0.993 | 2 |
| 96 | KHARSU 80A | 0.445 | 0.555 | 2 | 139 | BABAILACHHA | 0.003 | 0.997 | 2 |
| 97 | KASALATH | 0.538 | 0.462 | 1 | 140 | LUNISHREE | 0.019 | 0.981 | 2 |
| 98 | IR72 | 0.003 | 0.997 | 2 | 141 | MANDIRA | 0.008 | 0.992 | 2 |
| 99 | IR42 | 0.013 | 0.987 | 2 | 142 | ASANA | 0.004 | 0.996 | 2 |
| 100 | IR737074532 | 0.031 | 0.969 | 2 | 143 | PANIDHAN2 | 0.006 | 0.994 | 2 |
| 101 | BARASALI | 0.017 | 0.983 | 2 | 144 | TULASI | 0.004 | 0.996 | 2 |
| 102 | IR36 | 0.004 | 0.996 | 2 | 145 | SAVITRI | 0.004 | 0.996 | 2 |
| 103 | IC370811 | 0.049 | 0.951 | 2 | 146 | SWARNASUB1 | 0.004 | 0.996 | 2 |
| 104 | CHANGMAKA | 0.003 | 0.997 | 2 | 147 | BADDHANA | 0.993 | 0.007 | 1 |
| 105 | CHOWHALA | 0.003 | 0.997 | 2 | 148 | ASHUBHAJNA | 0.004 | 0.996 | 2 |
| 106 | CHANGMAN | 0.003 | 0.997 | 2 | 149 | BADGEDE | 0.003 | 0.997 | 2 |
| 107 | BABARAKSHAK | 0.004 | 0.996 | 2 | 150 | PADMA | 0.003 | 0.997 | 2 |
| 108 | JOHAINEANG | 0.004 | 0.996 | 2 | 151 | PANIKEKEUA | 0.006 | 0.994 | 2 |
| 109 | BARACHIRAMA | 0.008 | 0.992 | 2 | 152 | PANKEI | 0.010 | 0.990 | 2 |
| 110 | ASAMIRUPA | 0.005 | 0.995 | 2 | 153 | BADJHARLI | 0.006 | 0.994 | 2 |
| 111 | CHUKKANNA KUB | 0.002 | 0.998 | 2 | 154 | S183 | 0.009 | 0.991 | 2 |
| 112 | CHULTA BALUNG | 0.003 | 0.997 | 2 | 155 | SAMBHA-SUB1 | 0.006 | 0.994 | 2 |
| 113 | CHANGPHAI | 0.003 | 0.997 | 2 | 156 | JALNIDHI | 0.003 | 0.997 | 2 |
| 114 | CHASING | 0.997 | 0.003 | 1 | 157 | GOTHAWA | 0.006 | 0.994 | 2 |
| 115 | CHINGCHANG | 0.003 | 0.997 | 2 | 158 | KAJARAHAWA | 0.265 | 0.735 | 2 |
| 116 | MEGHI | 0.218 | 0.782 | 2 | 159 | SATHWA | 0.002 | 0.998 | 2 |
| 117 | ASHRALAKI | 0.008 | 0.992 | 2 | 160 | RAJBHOG | 0.010 | 0.990 | 2 |
| 118 | BAGAIKRA | 0.243 | 0.757 | 2 | 161 | SINGHARA | 0.009 | 0.991 | 2 |
| 119 | JOKHRU | 0.997 | 0.003 | 1 | 162 | TUNDAHIYA | 0.280 | 0.720 | 2 |
| 120 | CHOWMAI | 0.003 | 0.997 | 2 | 163 | SURAHA | 0.288 | 0.712 | 2 |
| 121 | CHANGNGAT | 0.003 | 0.997 | 2 | 164 | KARIYAWA | 0.004 | 0.996 | 2 |
| 122 | PANIKAKUA | 0.003 | 0.997 | 2 | 165 | DIHAWAN | 0.392 | 0.608 | 2 |
| 123 | CHUMANSAL39 | 0.01 | 0.990 | 2 | 166 | MOTI | 0.006 | 0.994 | 2 |
| 167 | SUGAPANKH | 0.007 | 0.993 | 2 | 174 | S138 | 0.004 | 0.996 | 2 |
| 168 | JADAHAN | 0.003 | 0.997 | 2 | 175 | S141 | 0.070 | 0.930 | 2 |
| 169 | DUDHALADU | 0.005 | 0.995 | 2 | 176 | S155 | 0.004 | 0.996 | 2 |
| 170 | S150 | 0.003 | 0.997 | 2 | 177 | MEGHABADALA | 0.002 | 0.998 | 2 |
| 171 | S172 | 0.005 | 0.995 | 2 | 178 | KALABUNDE | 0.007 | 0.993 | 2 |
| 172 | S148 | 0.033 | 0.967 | 2 | 179 | KOLAJOHA | 0.924 | 0.076 | 1 |
| 173 | S177 | 0.039 | 0.961 | 2 |  |  |  |  |  |

**Sub-populations:** 1 = Indica, 2= Japonica

**Table S3.** Population structure of 179 diverse rice genotypes at *K* = 4, resulting in four sub-populations of 1. Deep-water, 2. Indica, 3. Aus, 4. Japonica-Aromatic cultivar groups

| **Sr. no.** | **Variety** | **Fst 1** | **Fst 2** | **Fst 3** | **Fst 4** | **Sub-population** |
| --- | --- | --- | --- | --- | --- | --- |
| 5 | FR 43B | 0.989 | 0.006 | 0.002 | 0.002 | 1 |
| 7 | DHOLAMON 643 | 0.973 | 0.004 | 0.016 | 0.007 | 1 |
| 12 | LEMO | 0.928 | 0.010 | 0.050 | 0.012 | 1 |
| 19 | VANANVELLAI | 0.838 | 0.135 | 0.015 | 0.012 | 1 |
| 22 | ARC 614 | 0.767 | 0.097 | 0.067 | 0.069 | 1 |
| 24 | RENIKBATU | 0.848 | 0.006 | 0.012 | 0.133 | 1 |
| 25 | BIRPALA | 0.751 | 0.079 | 0.169 | 0.002 | 1 |
| 29 | MENYARHUNEI | 0.910 | 0.007 | 0.078 | 0.005 | 1 |
| 31 | PALENEMA | 0.701 | 0.287 | 0.005 | 0.006 | 1 |
| 32 | PALIANDEH | 0.969 | 0.021 | 0.008 | 0.003 | 1 |
| 33 | PANNEL | 0.973 | 0.019 | 0.006 | 0.002 | 1 |
| 34 | PANSANKO | 0.973 | 0.022 | 0.003 | 0.002 | 1 |
| 35 | PATAIM | 0.989 | 0.006 | 0.003 | 0.001 | 1 |
| 36 | PAYEMA | 0.990 | 0.006 | 0.002 | 0.002 | 1 |
| 37 | PECALO | 0.958 | 0.007 | 0.007 | 0.028 | 1 |
| 39 | TATAN | 0.981 | 0.015 | 0.003 | 0.001 | 1 |
| 40 | TIEBIMAH | 0.983 | 0.013 | 0.003 | 0.001 | 1 |
| 44 | YEIKOYON | 0.957 | 0.006 | 0.004 | 0.033 | 1 |
| 52 | JALDUNGI | 0.977 | 0.017 | 0.004 | 0.002 | 1 |
| 53 | NIRBOI | 0.972 | 0.021 | 0.004 | 0.002 | 1 |
| 54 | PAKRIBUNA | 0.946 | 0.039 | 0.010 | 0.005 | 1 |
| 55 | VADAI | 0.967 | 0.028 | 0.003 | 0.002 | 1 |
| 61 | BUNTOK | 0.929 | 0.060 | 0.007 | 0.004 | 1 |
| 62 | KRETEKSIREN | 0.908 | 0.008 | 0.057 | 0.028 | 1 |
| 64 | HOURAKANI | 0.922 | 0.006 | 0.017 | 0.056 | 1 |
| 66 | BR 11 | 0.868 | 0.119 | 0.009 | 0.004 | 1 |
| 68 | VAN | 0.985 | 0.004 | 0.008 | 0.003 | 1 |
| 73 | SOSSOKA | 0.985 | 0.006 | 0.007 | 0.003 | 1 |
| 74 | MOUSSAYA | 0.673 | 0.052 | 0.270 | 0.005 | 1 |
| 75 | KAOLACK | 0.948 | 0.010 | 0.036 | 0.007 | 1 |
| 78 | KARABONKA | 0.987 | 0.009 | 0.003 | 0.001 | 1 |
| 79 | EMPINGARA | 0.986 | 0.004 | 0.003 | 0.007 | 1 |
| 80 | ROMRUNTIK | 0.967 | 0.010 | 0.018 | 0.005 | 1 |
| 83 | SANGKHLA | 0.985 | 0.008 | 0.003 | 0.004 | 1 |
| 85 | TAHTONE | 0.952 | 0.036 | 0.005 | 0.006 | 1 |
| 87 | SIPULUTPAND | 0.984 | 0.005 | 0.006 | 0.005 | 1 |
| 101 | BARASALI | 0.468 | 0.265 | 0.261 | 0.006 | 1 |
| 104 | CHANGMAKA | 0.641 | 0.349 | 0.007 | 0.002 | 1 |
| 109 | BARACHIRAMA | 0.978 | 0.011 | 0.007 | 0.004 | 1 |
| 110 | ASAMIRUPA | 0.575 | 0.397 | 0.024 | 0.003 | 1 |
| 112 | CHULTABALUN | 0.990 | 0.005 | 0.003 | 0.002 | 1 |
| 113 | CHANGPHAI | 0.922 | 0.069 | 0.007 | 0.002 | 1 |
| 117 | ASHRALAKI | 0.978 | 0.012 | 0.006 | 0.004 | 1 |
| 126 | CHANGMUI | 0.872 | 0.120 | 0.004 | 0.004 | 1 |
| 128 | PANIDUBI | 0.988 | 0.005 | 0.005 | 0.002 | 1 |
| 131 | DHUSARIAHU | 0.946 | 0.017 | 0.008 | 0.030 | 1 |
| 133 | BARARANGA | 0.848 | 0.038 | 0.104 | 0.011 | 1 |
| 134 | KHAWJII | 0.974 | 0.022 | 0.003 | 0.001 | 1 |
| 135 | JAGANATHABA | 0.953 | 0.034 | 0.011 | 0.002 | 1 |
| 138 | MANOHAR SALI | 0.962 | 0.029 | 0.003 | 0.005 | 1 |
| 141 | MANDIRA | 0.785 | 0.184 | 0.013 | 0.018 | 1 |
| 144 | TULASI | 0.973 | 0.020 | 0.004 | 0.002 | 1 |
| 152 | PANKEI | 0.989 | 0.005 | 0.003 | 0.003 | 1 |
| 153 | BADJHARLI | 0.979 | 0.011 | 0.007 | 0.003 | 1 |
| 156 | JALNIDHI | 0.611 | 0.385 | 0.002 | 0.002 | 1 |
| 169 | DUDHALADU | 0.519 | 0.471 | 0.008 | 0.003 | 1 |
| 1 | TSAO WAN CHIN | 0.017 | 0.833 | 0.148 | 0.002 | 2 |
| 2 | AICHIHAIKWO | 0.006 | 0.990 | 0.003 | 0.001 | 2 |
| 3 | HSUNG TIENG | 0.095 | 0.900 | 0.003 | 0.001 | 2 |
| 9 | KURUWEE | 0.011 | 0.964 | 0.019 | 0.006 | 2 |
| 11 | IARI5829 | 0.003 | 0.992 | 0.003 | 0.002 | 2 |
| 13 | MADHUKAR | 0.011 | 0.766 | 0.218 | 0.005 | 2 |
| 14 | THUNMARHAMA | 0.007 | 0.986 | 0.003 | 0.004 | 2 |
| 15 | KARKAT I87 | 0.006 | 0.984 | 0.007 | 0.002 | 2 |
| 17 | POKKALI | 0.009 | 0.913 | 0.071 | 0.007 | 2 |
| 18 | KOTTAMALI | 0.013 | 0.981 | 0.004 | 0.002 | 2 |
| 20 | NAALUMOLIKA | 0.009 | 0.982 | 0.004 | 0.005 | 2 |
| 30 | PAIAIM | 0.133 | 0.511 | 0.350 | 0.007 | 2 |
| 38 | TAGBA | 0.148 | 0.497 | 0.349 | 0.006 | 2 |
| 41 | GODAHEENATI | 0.004 | 0.980 | 0.002 | 0.013 | 2 |
| 43 | TETEP | 0.010 | 0.955 | 0.004 | 0.032 | 2 |
| 45 | HEENSULAI | 0.006 | 0.904 | 0.006 | 0.083 | 2 |
| 46 | JAYMIS | 0.068 | 0.904 | 0.023 | 0.004 | 2 |
| 47 | KALUWEE | 0.008 | 0.983 | 0.005 | 0.003 | 2 |
| 48 | MADAL | 0.012 | 0.967 | 0.017 | 0.004 | 2 |
| 49 | MAHADIKWEE | 0.018 | 0.818 | 0.163 | 0.002 | 2 |
| 50 | USABATAPOLA | 0.007 | 0.989 | 0.003 | 0.002 | 2 |
| 56 | GOTA | 0.237 | 0.741 | 0.005 | 0.017 | 2 |
| 58 | CR 1009 | 0.380 | 0.600 | 0.019 | 0.002 | 2 |
| 59 | CHAKIA 59 | 0.012 | 0.979 | 0.005 | 0.003 | 2 |
| 60 | FRG1 | 0.012 | 0.982 | 0.005 | 0.001 | 2 |
| 65 | NALDAK | 0.056 | 0.518 | 0.419 | 0.007 | 2 |
| 67 | ZOBGUI | 0.029 | 0.875 | 0.008 | 0.087 | 2 |
| 70 | SITAMATA | 0.007 | 0.987 | 0.003 | 0.003 | 2 |
| 71 | LIU-TIAO-NU | 0.008 | 0.985 | 0.005 | 0.002 | 2 |
| 72 | MA-ZHAN (RED) | 0.005 | 0.988 | 0.004 | 0.002 | 2 |
| 84 | SANTIBAHN | 0.109 | 0.885 | 0.004 | 0.001 | 2 |
| 86 | WAHNG | 0.414 | 0.572 | 0.005 | 0.010 | 2 |
| 92 | KHAOHLANON | 0.213 | 0.669 | 0.016 | 0.101 | 2 |
| 93 | KHAOMINELAR | 0.027 | 0.967 | 0.004 | 0.002 | 2 |
| 94 | IR 64 | 0.018 | 0.932 | 0.005 | 0.045 | 2 |
| 95 | SWARNA | 0.011 | 0.983 | 0.003 | 0.003 | 2 |
| 98 | IR 72 | 0.022 | 0.971 | 0.005 | 0.002 | 2 |
| 99 | IR 42 | 0.006 | 0.986 | 0.002 | 0.006 | 2 |
| 100 | IR 737074532 | 0.348 | 0.613 | 0.003 | 0.036 | 2 |
| 102 | IR 36 | 0.007 | 0.988 | 0.004 | 0.002 | 2 |
| 103 | IC 370811 | 0.010 | 0.938 | 0.026 | 0.026 | 2 |
| 105 | CHOWHALA | 0.007 | 0.989 | 0.002 | 0.002 | 2 |
| 106 | CHANGMAN | 0.027 | 0.968 | 0.003 | 0.002 | 2 |
| 107 | BABARAKSHAK | 0.021 | 0.972 | 0.006 | 0.002 | 2 |
| 108 | JOHAINEANG | 0.090 | 0.904 | 0.003 | 0.003 | 2 |
| 111 | CHUKKANNAKU | 0.074 | 0.920 | 0.005 | 0.001 | 2 |
| 115 | CHINGCHANG | 0.026 | 0.967 | 0.006 | 0.002 | 2 |
| 120 | CHOWMAI | 0.365 | 0.624 | 0.007 | 0.003 | 2 |
| 121 | CHANGNGAT | 0.251 | 0.743 | 0.004 | 0.002 | 2 |
| 122 | PANIKAKUA | 0.252 | 0.743 | 0.004 | 0.002 | 2 |
| 123 | CHUMANSAL 39 | 0.008 | 0.934 | 0.048 | 0.010 | 2 |
| 130 | ASAMIA | 0.008 | 0.982 | 0.006 | 0.004 | 2 |
| 136 | JALKANTHI | 0.040 | 0.932 | 0.027 | 0.002 | 2 |
| 137 | CHUDI | 0.007 | 0.983 | 0.006 | 0.004 | 2 |
| 139 | BABAILACHHA | 0.015 | 0.974 | 0.009 | 0.002 | 2 |
| 140 | LUNISHREE | 0.107 | 0.488 | 0.400 | 0.005 | 2 |
| 142 | ASANA | 0.045 | 0.946 | 0.007 | 0.002 | 2 |
| 143 | PANIDHAN 2 | 0.007 | 0.987 | 0.003 | 0.003 | 2 |
| 145 | SAVITRI | 0.302 | 0.678 | 0.018 | 0.002 | 2 |
| 146 | SWARNASUB1 | 0.008 | 0.987 | 0.003 | 0.002 | 2 |
| 148 | ASHUBHAJNA | 0.010 | 0.978 | 0.010 | 0.002 | 2 |
| 149 | BADGEDE | 0.013 | 0.979 | 0.006 | 0.002 | 2 |
| 150 | PADMA | 0.015 | 0.977 | 0.006 | 0.002 | 2 |
| 151 | PANIKEKEUA | 0.049 | 0.903 | 0.045 | 0.003 | 2 |
| 154 | S 183 | 0.007 | 0.856 | 0.128 | 0.009 | 2 |
| 155 | SAMBHA-SUB1 | 0.015 | 0.974 | 0.008 | 0.004 | 2 |
| 157 | GOTHAWA | 0.050 | 0.943 | 0.003 | 0.005 | 2 |
| 159 | SATHWA | 0.058 | 0.936 | 0.005 | 0.001 | 2 |
| 160 | RAJBHOG | 0.357 | 0.572 | 0.052 | 0.018 | 2 |
| 161 | SINGHARA | 0.061 | 0.924 | 0.008 | 0.007 | 2 |
| 164 | KARIYAWA | 0.059 | 0.934 | 0.005 | 0.002 | 2 |
| 166 | MOTI | 0.014 | 0.975 | 0.008 | 0.003 | 2 |
| 167 | SUGAPANKH | 0.049 | 0.943 | 0.003 | 0.006 | 2 |
| 168 | JADAHAN | 0.024 | 0.968 | 0.006 | 0.002 | 2 |
| 170 | S 150 | 0.005 | 0.989 | 0.004 | 0.001 | 2 |
| 171 | S 172 | 0.006 | 0.986 | 0.006 | 0.002 | 2 |
| 172 | S 148 | 0.012 | 0.967 | 0.004 | 0.018 | 2 |
| 173 | S 177 | 0.009 | 0.965 | 0.003 | 0.023 | 2 |
| 174 | S 138 | 0.011 | 0.983 | 0.003 | 0.003 | 2 |
| 175 | S 141 | 0.073 | 0.821 | 0.012 | 0.094 | 2 |
| 176 | S 155 | 0.009 | 0.985 | 0.004 | 0.002 | 2 |
| 177 | MEGHA BADALA | 0.145 | 0.851 | 0.003 | 0.001 | 2 |
| 178 | KALA BUNDE | 0.097 | 0.856 | 0.037 | 0.010 | 2 |
| 4 | DULAR | 0.006 | 0.004 | 0.988 | 0.002 | 3 |
| 6 | FR 13A | 0.005 | 0.010 | 0.982 | 0.003 | 3 |
| 21 | ARC 12172 | 0.014 | 0.013 | 0.971 | 0.002 | 3 |
| 28 | AUS 287 | 0.011 | 0.003 | 0.827 | 0.160 | 3 |
| 42 | KALONCHI | 0.005 | 0.005 | 0.988 | 0.002 | 3 |
| 51 | AUSBORO | 0.002 | 0.002 | 0.992 | 0.003 | 3 |
| 69 | KHAIYAN | 0.040 | 0.024 | 0.934 | 0.002 | 3 |
| 76 | NCS 348 | 0.003 | 0.002 | 0.994 | 0.002 | 3 |
| 96 | KHARSU80A | 0.002 | 0.003 | 0.993 | 0.002 | 3 |
| 97 | KASALATH | 0.003 | 0.003 | 0.986 | 0.008 | 3 |
| 116 | MEGHI | 0.012 | 0.010 | 0.975 | 0.002 | 3 |
| 118 | BAGAIKRA | 0.069 | 0.039 | 0.889 | 0.003 | 3 |
| 127 | DUBGELONG | 0.011 | 0.009 | 0.978 | 0.002 | 3 |
| 158 | KAJARAHAWA | 0.013 | 0.178 | 0.804 | 0.006 | 3 |
| 162 | TUNDAHIYA | 0.007 | 0.003 | 0.989 | 0.002 | 3 |
| 163 | SURAHA | 0.017 | 0.005 | 0.971 | 0.006 | 3 |
| 165 | DIHAWAN | 0.002 | 0.003 | 0.993 | 0.002 | 3 |
| 8 | MAINTIMOLOT | 0.002 | 0.001 | 0.002 | 0.995 | 4 |
| 10 | MOROBEREKAN | 0.002 | 0.001 | 0.002 | 0.995 | 4 |
| 16 | JC 1 | 0.008 | 0.011 | 0.007 | 0.974 | 4 |
| 23 | LABELLE | 0.002 | 0.002 | 0.002 | 0.995 | 4 |
| 26 | BINULAWAN | 0.002 | 0.001 | 0.002 | 0.995 | 4 |
| 27 | JALMAGNA | 0.102 | 0.258 | 0.040 | 0.600 | 4 |
| 57 | MARS | 0.001 | 0.001 | 0.002 | 0.995 | 4 |
| 63 | AZUCENA | 0.004 | 0.003 | 0.005 | 0.987 | 4 |
| 77 | KAMNAM | 0.013 | 0.014 | 0.002 | 0.971 | 4 |
| 81 | LEMONT | 0.002 | 0.002 | 0.002 | 0.994 | 4 |
| 82 | LATSIKA | 0.072 | 0.134 | 0.044 | 0.750 | 4 |
| 88 | AKITAKOMACHI | 0.001 | 0.001 | 0.001 | 0.996 | 4 |
| 89 | KATY | 0.035 | 0.024 | 0.064 | 0.877 | 4 |
| 90 | M 202 | 0.001 | 0.001 | 0.002 | 0.996 | 4 |
| 91 | KARNAL LOCAL | 0.001 | 0.002 | 0.203 | 0.794 | 4 |
| 114 | CHASING | 0.002 | 0.001 | 0.002 | 0.995 | 4 |
| 119 | JOKHRU | 0.002 | 0.001 | 0.002 | 0.995 | 4 |
| 124 | JAHABHOG | 0.003 | 0.003 | 0.004 | 0.991 | 4 |
| 125 | BADKODI | 0.002 | 0.002 | 0.003 | 0.993 | 4 |
| 129 | MEGHRAJ | 0.001 | 0.001 | 0.002 | 0.996 | 4 |
| 132 | BAGAGOHA | 0.020 | 0.019 | 0.028 | 0.932 | 4 |
| 147 | BAD DHANA | 0.011 | 0.010 | 0.014 | 0.966 | 4 |
| 179 | KOLA JOHA | 0.011 | 0.101 | 0.089 | 0.799 | 4 |

**Sub-populations:** 1= Deep-water , 2= Indica, 3= Aus, 4= Japonica-Aromatica

**Table S4.**  PCR amplification results with the three *SUB1* genes. Color coding of cells indicates successful amplification of the desired size product.

| **Sr. no.** | **PCR well** | **Variety** | **Sequencing barcode** | **Amplification results** | | |
| --- | --- | --- | --- | --- | --- | --- |
|  |  |  |  | ***Sub1A*** | ***Sub1B*** | ***Sub1C*** |
|  | A1 | TSAO WAN CHING | 1 |  |  |  |
|  | A2 | AI CHIH AI KWOH | 2 |  |  |  |
|  | A3 | HSUNG TIENG | 3 |  |  |  |
|  | A4 | DULAR | 4 |  |  |  |
|  | A5 | FR 13 A | 5 |  |  |  |
|  | A6 | FR 13 B | 6 |  |  |  |
|  | A7 | DHOLAMON 64-3 | 7 |  |  |  |
|  | A8 | KARKATI 87 | 8 |  |  |  |
|  | A9 | JC1 | 9 |  |  |  |
|  | A10 | MAINTIMOLOTSY | 10 |  |  |  |
|  | A11 | KURUWEE | 11 |  |  |  |
|  | A12 | MOROBEREKAN | 12 |  |  |  |
|  | B1 | IARI 5825 | 13 |  |  |  |
|  | B2 | LEMO | 14 |  |  |  |
|  | B3 | MADHUKAR | 15 |  |  |  |
|  | B4 | THUNMAR HAMARA | 16 |  |  |  |
|  | B5 | POKKALI | 17 |  |  |  |
|  | B6 | KOTTAMALI | 18 |  |  |  |
|  | B7 | VANAN VELLAI | 19 |  |  |  |
|  | B8 | NAALUMOLON KARUPPAN | 20 |  |  |  |
|  | B10 | ARC 12172 | 21 |  |  |  |
|  | B11 | ARC 614 | 22 |  |  |  |
|  | B12 | LABELLE | 23 |  |  |  |
|  | C1 | RENIK BATU | 24 |  |  |  |
|  | C2 | BIRPALA | 25 |  |  |  |
|  | C3 | BINULAWAN | 26 |  |  |  |
|  | C5 | JALMAGNA | 27 |  |  |  |
|  | C6 | AUS 287 | 28 |  |  |  |
|  | C8 | MENYARHUNEI | 29 |  |  |  |
|  | C10 | PAIAM | 30 |  |  |  |
|  | C11 | PALENEMA | 31 |  |  |  |
|  | C12 | PALIANDEH | 32 |  |  |  |
|  | D1 | PANNEL | 33 |  |  |  |
|  | D2 | PANSANKO | 34 |  |  |  |
|  | D3 | PATAIM | 35 |  |  |  |
|  | D4 | PAYEMA | 36 |  |  |  |
|  | D5 | PECALO | 37 |  |  |  |
|  | D6 | TAGBA | 38 |  |  |  |
|  | D7 | TATAN | 39 |  |  |  |
|  | D8 | TIEBIMAH | 40 |  |  |  |
|  | D9 | GODA HEENATI | 41 |  |  |  |
|  | D10 | KALONCHI | 42 |  |  |  |
|  | D12 | TETEP | 43 |  |  |  |
|  | E1 | YEIKOYON | 44 |  |  |  |
|  | E2 | HEEN SULAI | 45 |  |  |  |
|  | E3 | JAYMIS | 46 |  |  |  |
|  | E4 | KALUWEE | 47 |  |  |  |
|  | E5 | MADAL | 48 |  |  |  |
|  | E6 | MAHA DIKWEE | 49 |  |  |  |
|  | E7 | USA BATAPOLA AL | 50 |  |  |  |
|  | E9 | AUSBORO | 52 |  |  |  |
|  | E10 | JALDUNGI | 53 |  |  |  |
|  | E11 | NIRBOI | 54 |  |  |  |
|  | E12 | PAKRIBUNA | 55 |  |  |  |
|  | F1 | VADAI | 56 |  |  |  |
|  | F2 | GOTA | 57 |  |  |  |
|  | F3 | MARS | 58 |  |  |  |
|  | F4 | CR 1009 | 59 |  |  |  |
|  | F5 | CHAKIA 59 | 60 |  |  |  |
|  | F6 | FRG 1 | 61 |  |  |  |
|  | F7 | BUNTOK | 62 |  |  |  |
|  | F8 | KRETEK SIRENDAH MERA | 63 |  |  |  |
|  | F10 | AZUCENA | 64 |  |  |  |
|  | F11 | HOURA KANI | 65 |  |  |  |
|  | F12 | NALDAK | 66 |  |  |  |
|  | G1 | BR11 | 67 |  |  |  |
|  | G2 | ZOBGUI | 68 |  |  |  |
|  | G3 | VAN | 69 |  |  |  |
|  | G4 | KHAIYAN | 70 |  |  |  |
|  | G5 | SITA MATA | 71 |  |  |  |
|  | G6 | LIU-TIAO-NU | 72 |  |  |  |
|  | G7 | MA ZHAN (RED) | 73 |  |  |  |
|  | G8 | SOSSOKA | 74 |  |  |  |
|  | G9 | MOUSSAYA | 75 |  |  |  |
|  | G10 | KAOLACK | 76 |  |  |  |
|  | G11 | NCS 348 | 77 |  |  |  |
|  | G12 | KAMNAM | 78 |  |  |  |
|  | H2 | KARABONKA | 79 |  |  |  |
|  | H3 | EMPING ARA | 80 |  |  |  |
|  | H4 | ROM RUNTIK | 81 |  |  |  |
|  | H5 | LEMONT | 82 |  |  |  |
|  | H6 | LATSIKA | 83 |  |  |  |
|  | H7 | SANG KHLA | 84 |  |  |  |
|  | H8 | SANTI BAHN | 85 |  |  |  |
|  | H9 | TAH TONE | 86 |  |  |  |
|  | H10 | WAHNG | 87 |  |  |  |
|  | H11 | SIPULUT PANDAN | 88 |  |  |  |
|  | A1 | AKITAKOMACHI | 1 |  |  |  |
|  | A2 | KATY | 2 |  |  |  |
|  | A3 | M 202 | 3 |  |  |  |
|  | A4 | KARNAL LOCAL | 4 |  |  |  |
|  | A5 | KHAO HLAN ON | 5 |  |  |  |
|  | A6 | KHAO MINE LAR | 6 |  |  |  |
|  | A7 | IR 64 | 7 |  |  |  |
|  | A8 | SWARNA | 8 |  |  |  |
|  | A9 | KHARSU 80A: Oryza spc | 9 |  |  |  |
|  | A10 | KASALATH | 10 |  |  |  |
|  | A11 | IR 72 | 11 |  |  |  |
|  | A12 | IR 42 | 12 |  |  |  |
|  | B1 | IR 73707-45-3-23 | 13 |  |  |  |
|  | B2 | BARA SALI | 14 |  |  |  |
|  | B3 | IR 36 | 15 |  |  |  |
|  | B4 | IC370811 | 16 |  |  |  |
|  | B5 | CHANGMAKA | 17 |  |  |  |
|  | B6 | CHOW HA LA | 18 |  |  |  |
|  | B8 | CHANGMAN | 20 |  |  |  |
|  | B9 | BABARAKSHA KARO | 21 |  |  |  |
|  | B10 | JOHAINEANG (WET) | 22 |  |  |  |
|  | B11 | BARACHIRAMARA | 23 |  |  |  |
|  | B12 | ASAMIRUPA | 24 |  |  |  |
|  | C1 | CHUKKANNA KUMBALOAN | 25 |  |  |  |
|  | C2 | CHULTA BALUNGA (WILD) | 26 |  |  |  |
|  | C3 | CHANGPHAI | 27 |  |  |  |
|  | C4 | CHASING | 28 |  |  |  |
|  | C5 | CHING CHANG | 29 |  |  |  |
|  | C6 | MEGHI | 30 |  |  |  |
|  | C7 | ASHRA LAKI | 31 |  |  |  |
|  | C8 | BAGA IKRA | 32 |  |  |  |
|  | C9 | JOKHRU | 33 |  |  |  |
|  | C10 | CHOWMAI | 34 |  |  |  |
|  | C11 | CHANGNGAT | 35 |  |  |  |
|  | C12 | PANIKAKUA | 36 |  |  |  |
|  | D1 | CHUMANSAL 39 | 37 |  |  |  |
|  | D2 | JAHABHOG | 38 |  |  |  |
|  | D3 | BAD KODI | 39 |  |  |  |
|  | D4 | CHANG MUI | 40 |  |  |  |
|  | D5 | DUB GELONG | 41 |  |  |  |
|  | D6 | PANIDUBI | 42 |  |  |  |
|  | D7 | MEGHARAJ | 43 |  |  |  |
|  | D8 | ASAMIA | 44 |  |  |  |
|  | D9 | DHUSARI AHU | 45 |  |  |  |
|  | D11 | BAGA GOHA | 46 |  |  |  |
|  | D12 | BARA RANGA | 47 |  |  |  |
|  | E1 | KHAWJII | 48 |  |  |  |
|  | E2 | JAGANATHA BALLAVA | 49 |  |  |  |
|  | E3 | JALKANTHI | 50 |  |  |  |
|  | E4 | CHUDI | 51 |  |  |  |
|  | E5 | MANOHAR SALI | 52 |  |  |  |
|  | E6 | BABAI LACHHA | 53 |  |  |  |
|  | E7 | LUNISHREE | 54 |  |  |  |
|  | E8 | MANDIRA | 55 |  |  |  |
|  | E9 | ASANA | 56 |  |  |  |
|  | E10 | PANI DHAN 2 | 57 |  |  |  |
|  | E11 | TULASI | 58 |  |  |  |
|  | E12 | SAVITRI | 59 |  |  |  |
|  | F1 | SWARNA SUB1 | 60 |  |  |  |
|  | F2 | BAD DHANA | 61 |  |  |  |
|  | F3 | ASHU BHAJNA | 62 |  |  |  |
|  | F4 | BAD GEDE | 63 |  |  |  |
|  | F5 | PADMA | 64 |  |  |  |
|  | F6 | PANIKEKEUA | 65 |  |  |  |
|  | F7 | PANKEI | 66 |  |  |  |
|  | F8 | BAD JHARLI | 67 |  |  |  |
|  | F9 | S 183 | 68 |  |  |  |
|  | F10 | SAMBHA SUB1 | 69 |  |  |  |
|  | F11 | JALNIDHI | 70 |  |  |  |
|  | F12 | GOTHAWA | 71 |  |  |  |
|  | G1 | KAJARAHAWA | 72 |  |  |  |
|  | G2 | SATHWA | 73 |  |  |  |
|  | G3 | RAJBHOG | 74 |  |  |  |
|  | G4 | SINGHARA | 75 |  |  |  |
|  | G5 | TUNDAHIYA | 76 |  |  |  |
|  | G6 | SURAHA | 77 |  |  |  |
|  | G7 | KARIYWA | 78 |  |  |  |
|  | G8 | DIHAWAN | 79 |  |  |  |
|  | G9 | MOTI | 80 |  |  |  |
|  | G10 | SUGAPANKH | 81 |  |  |  |
|  | G12 | JADHAN | 82 |  |  |  |
|  | H2 | DUDHA LADU | 83 |  |  |  |
|  | H3 | S 150 | 84 |  |  |  |
|  | H4 | S 172 | 85 |  |  |  |
|  | H5 | S 148 | 86 |  |  |  |
|  | H6 | S 177 | 87 |  |  |  |
|  | H7 | S 138 | 88 |  |  |  |
|  | H8 | KOLA JOHA | 89 |  |  |  |
|  | H9 | S 141 | 90 |  |  |  |
|  | H10 | S 155 | 91 |  |  |  |
|  | H11 | MEGHA BADALA | 92 |  |  |  |
|  | H12 | KALA BUNDE | 93 |  |  |  |
|  |  | **Total** |  | **96** | **110** | **174** |

**Table S5:** Comparison of SNP variation in the three *Sub1* genes identified by targeted re-sequencing of 179 cultivars in the present study with those identified by analysis of 66 whole genome sequences of cultivated and wild rice published by Zhao et al. (2018). The SNP positions are indicated with respect to ATG start codon. Colour shading indicates SNPs common to both sets of genotypes (green), unique to 179 cultivar set (blue) and unique to 66 genome set (red).

| **Sr. no.** | **SNP Position**  **(66 Genome)** | **SNP Position (179 cv.)** | **Reference**  **Base** | **Variant**  **Base** | **Common/ Unique**  **Genotype Set** |
| --- | --- | --- | --- | --- | --- |
| ***Sub1A* gene** | | | | | |
|  | 556 | 556 | T | C | Common |
|  | 678 | 678 | A | G | Common |
|  | - | 878 | C | G | 179 cv |
|  | 1121 | 1121 | G | C | Common |
|  | 1127 | 1127 | C | G | Common |
|  | 1277 | 1277 | G | A | Common |
|  | 1385-1386 | - | CG | TG/CA | 66 genome. W0141 |
|  | 1407 | 1407 | A | G | Common |
|  | 1567 | - | T | C | 66 genome W0141 |
| ***Sub1B* gene** | | | | | |
|  | - | -643 | C | T | 179 cv. |
|  | -449 | - | G | A | 66 genome, GP540, W1754 |
|  | -422 | - | T | G | 66 genome, HP362-2, WYG7 |
|  | -411 | - | GT | TA | 66 genome, HP362-2, WYG7 |
|  | -404 | - | G | C | 66 genome, HP362-2, WYG7 |
|  | -394 | - | T | A | 66 genome, HP362-2, WYG7 |
|  | -392 | - | A | T | 66 genome, HP362-2, WYG7 |
|  | -354 | - | C | A | 66 genome, GP567, HP14 |
|  | -341 | - | C | A | 66 genome, HP362-2, WYG7 |
|  | -322 | - | C | T | 66 genome, HP362-2, WYG7 |
|  | - | -282 | T | G | 179 cv. |
|  | -270 | - | C | A | 66 genome, HP362-2, WYG7 |
|  | -263 | - | C | A | 66 genome, HP362-2, WYG7 |
|  | -235 | - | G | C | 66 genome, HP362-2, WYG7 |
|  | -232 | - | C | G | 66 genome, HP362-2, WYG7 |
|  | -217 | - | C | A | 66 genome, HP362-2, WYG7 |
|  | -165 | - | A | T | 66 genome, HP362-2, WYG7 |
|  | -96 | - | G | T | 66 genome, W177 |
|  | -74 | - | A | G | 66 genome, W0141 |
|  | 31 | - | C | T | 66 genome, W1979, GP22 |
|  | 39 | - | A | G | 66 genome, W0141 |
|  | 43 | - | G | C | 66 genome, Kasalath |
|  | 115 | - | C | T | 66 genome, WYG7, UR28 |
|  | 171 | - | T | A | 66 genome, W1979, GP22 |
|  | 186 | - | A | G | 66 genome, W1979, GP22, |
|  | 232 | - | G | C | 66 genome, W1979, GP22 |
|  | - | 280 | G | A | 179 cv. |
|  | 405 | - | C | T | 66 genome, HP362-2, WYG7 |
|  | 492 | - | G | T | 66 genome, HP362-2, WYG7 |
|  | 728 | - | T | C | 66 genome, HP362-2, WYG7 |
|  | 737 | - | A | G | 66 genome, HP362-2, WYG7 |
|  | 759 | - | G | C | 66 genome, HP362-2, WYG7 |
|  | 801 | - | A | T | 66 genome, HP362-2, WYG7 |
|  | - | 771 | G | A | 179 cv |
|  | 942 | 835 | A | G/C | Common |
|  | 962 | 855 | T | C | Common |
|  | 977 | 864 | T | C | Common |
| ***Sub1C* gene** | | | | | |
|  | - | -38 | A | T | 179 cv. |
|  | - | -35 | - | AACA | 179 cv. |
|  | - | -21 | G | AACCCACCAATCGAATCG | 179 cv. |
|  | -14 | -14 | G | C | Common |
|  | -9 | -9 | A | C | Common |
|  | -6 | -6 | A | G | Common |
|  | -1 | -1 | G | C | Common |
|  | 25 | 25 | C | T | Common |
|  | 26 | - | G | A | 66 genome, GP124, WyG7 |
|  | 36 | 36 | C | G | Common |
|  | - | 37 | - | TCCTCCTCCTCCC | 179 cv. |
|  | 39 | 39 | C | G | Common |
|  | 45 | - | C | G | 66 genome, GP124, WyG7 |
|  | 92 | 92 | T | C | Common |
|  | 95 | 95 | C | T | Common |
|  | 98 | - | T | C | 66 genome, GP124, WyG7 |
|  | 142 | 142 | G | T | Common |
|  | 148 | - | C | G | 66 genome, GP124, WyG7 |
|  | 151 | - | C | G | 66 genome, GP124, WyG7 |
|  | 160 | 160 | G | G | Common |
|  | 163 | 163 | G | C | Common |
|  | 163 | 171 | G | T | Common |
|  | 169 | - | G | G | 66 genome, GP124, WyG7 |
|  | 171 | 174 | C | G | Common |
|  | 174 | 177 | C | T | Common |
|  | 177 | - | C | T | 66 genome, GP124, WyG7 |
|  | 177 | 186 | T | C | Common |
|  | 180 | - | G | C | 66 genome, GP124, WyG7 |
|  | 186 | 193 | C | T | Common |
|  | 192 | - | T | C | 66 genome, GP22, GP772 |
|  | 199 | - | C | T | 66 genome, GP124, WyG7 |
|  | 202 | 202 | A | G | Common |
|  | 208 | - | G | A | 66 genome, GP124, WyG7 |
|  | 208 | 208 | A | G | Common |
|  | 246 | 246 | G | A | Common |
|  | 252 | - | G | A | 66 genome, W1754, GP62, |
|  | 256 | - | G | A | 66 genome, W1739 |
|  | 313 | 313 | C | T | Common |
|  | 319 | - | G | A | 66 genome, HP396 |
|  | 326 | 326 | G | A | Common |
|  | 335 | - | A | G | 66 genome, GP124, WyG7 |
|  | 361 | - | G | A | 66 genome, W1754 |
|  | - | 409 | C | T | 179 cv. |
|  | 459 | 459 | C | T | Common |
|  | 467 | - | C | T | 66 genome, W0123, GP62 |
|  | - | 495 | C | T | 179 cv. |
|  | 504 | - | C | T | 66 genome, W0123 GP62 |
|  | 527 | - | G | A | 66 genome, HP577 |
|  | 566 | - | G | A | 66 genome, W177 |
|  | 577 | 577 | A | G | Common |
|  | 580 | 580 | A | G | Common |
|  | 583 | 583 | A | G | Common |
|  | - | 586 | A | G | 179 cv |
|  | 588 | 588 | G | C | Common |
|  | - | 589 | C | G | 179 cv |
|  | 592 | - | A | G | 66 genome, GP540 WyG7 |
|  | 598 | 598 | - | GGCGGCT | Common |
|  | - | 607 | G | A | 179 cv. |
|  | - | 635 | G | A | 179 cv. |
|  | 650 | - | G | A | 66 genome, GP772 |

Sub1A: Total 9, 6 common, 1 unique to 179 cv., 2 unique to 66 genome

Sub1B: Total 37, 3 common, 4 unique to 179 cv., 29 unique to 66 genome

Sub1C: Total 60, 28 common, 10 unique to 179 cv., 22 unique to 66 genome

**Supplementary Table S6**. List of Sub1C rice genes in other species in Non-redundant green plant database. Total 22 homologous genes of Sub1A, Sub1B and Sub1C were in the 12 different species other than *Oryza sativa*.

| **S. No.** | **Query Sequence** | **Matching gene in the NR Database** | **Species** | **Bit Score** |
| --- | --- | --- | --- | --- |
| 1 | Sub1A | CBX88537.1 hypothetical protein | *Oryza rufipogon* | 340 |
| 2 | Sub1A | CBX88538.1 hypothetical protein | *Oryza rufipogon* | 340 |
| 3 | Sub1A | CBX88539.1 hypothetical protein | *Oryza rufipogon* | 340 |
| 4 | Sub1A | XP_015696560.1 ethylene-responsive transcription factor RAP2-2-like | *Oryza brachyantha* | 124 |
| 5 | Sub1B | ACA42754.1 Sub1B, partial | *Oryza rufipogon* | 144 |
| 6 | Sub1C | ACA42756.1 Sub1C, partial | *Oryza rufipogon* | 182 |
| 7 | Sub1C | ADG58076.1 transcription factor | *Lycoris longituba* | 100 |
| 8 | Sub1C | AEG77299.1 hypothetical protein | *Oryza rhizomatis* | 206 |
| 9 | Sub1C | AGE92355.1 ethylene response factor 1 | *Ipomoea batatas* | 100 |
| 10 | Sub1C | CBX88542.1 hypothetical protein, partial | *Oryza eichingeri* | 194 |
| 11 | Sub1C | CBX88543.1 hypothetical protein, partial | *Oryza eichingeri* | 195 |
| 12 | Sub1C | CBX88544.1 hypothetical protein, partial | *Oryza eichingeri* | 188 |
| 13 | Sub1C | CBX88545.1 hypothetical protein, partial | *Oryza eichingeri* | 195 |
| 14 | Sub1C | CBX88546.1 hypothetical protein, partial | *Oryza eichingeri* | 194 |
| 15 | Sub1C | KMZ68834.1 Ethylene responsive transcription factor 2b | *Zostera marina* | 100 |
| 16 | Sub1C | RDX72597.1 GDSL esterase/lipase 5, partial | *Mucuna pruriens* | 104 |
| 18 | Sub1C | XP_010552190.1 PREDICTED: ethylene-responsive transcription factor RAP2-12-like isoform X1 | *Tarenaya hassleriana* | 101 |
| 17 | Sub1C | XP_009605490.1 PREDICTED: ethylene-responsive transcription factor RAP2-12-like | *Nicotiana tomentosiformis* | 100 |
| 19 | Sub1C | XP_010552191.1 PREDICTED: ethylene-responsive transcription factor RAP2-12-like isoform X2 | *Tarenaya hassleriana* | 100 |
| 20 | Sub1C | XP_016515225.1 PREDICTED: ethylene-responsive transcription factor RAP2-12-like | *Nicotiana tabacum* | 100 |
| 21 | Sub1C | XP_019194413.1 PREDICTED: ethylene-responsive transcription factor RAP2-12 | *Ipomoea nil* | 100 |
| 22 | Sub1C | XP_019198756.1 PREDICTED: ethylene-responsive transcription factor RAP2-12-like | *Ipomoea nil* | 100 |

**Supplementary Table 7.** Copy number of rice *Sub1C* gene homologs in other plant species with complete genome sequence information

| **S. No.** | **Clade/**  **Class** | **Organism Name** | **No. of genes** | **Chromosomal coordinates** |
| --- | --- | --- | --- | --- |
| 1 | Monocot | *Sorgham bicolor* | 1 | Chr 3: 15604620-15605901 |
| 2 |  | *Hordeum vulgare* | 1 | Chr 5H:562157978-562159066 |
| 3 |  | *Brachypodium distachyon* | 2 | Bd2:10208175-10217282 |
|  |  |  |  | Bd5:9052631-9054342 |
| 4 |  | *Zea mays* | 3 | Chr2:214344857-214345995 |
|  |  |  |  | Chr3:56326099-56327373 |
|  |  |  |  | Chr8:35566541-35567721 |
| 5 |  | *Triticum aestivum* | 2 | ta_iwgsc_5bl_v1_10923980:5890-7050 |
|  |  |  |  | ta_iwgsc_5dl_v1_4507574:896-1985 |
| 6 |  | *Musa acuminata* | 2 | chr8:34598151-34599017 |
|  |  |  |  | chrUn_random:75888827-75889554 |
| 7 |  | *Ananas comosus* | 1 | LG17:1034914-1036596 |
| 8 | Dicot | *Camellia sinensis* | 1 | scaffold00245:15032-16012 |
| 9 |  | *Glycine max* | 2 | Gm06:47477331-47478610 |
|  |  |  |  | Gm12:11723606-11724665 |
| 10 |  | *Arabidopsis thaliana* | 0 | - |
| 11 |  | *Vitis venifera* | 0 | - |
| 12 |  | *Medicago truncatula* | 0 | - |
| 13 |  | *Populus trichocarpa* | 0 | - |
| 14 |  | *Cajanus cajan* | 0 | - |
| 15 |  | *Solanum lycopersicum* | 0 | - |
| 16 |  | *Polianthes tuberosa* | 0 | - |
| 17 |  | *Medicago truncatula* | 0 | - |
| 18 | Tracheophyta | *Amborella trichopoda* | 0 | - |
| 19 | Bryopsida | *Physcomitrella patens* | 0 | - |
| 20 | Chlorophyceae | *Chlamydomonas reinhardtii* | 0 | - |

**Table S8. Details of 35 SNPs and 3 InDels identified by targeted re-sequencing of *Sub1C* gene and their haplotypes in 174 rice cultivars**

**(Provided as separate Excel File)**

**Table S9. Protein haplotypes of the *Sub1A, Sub1B* and *Sub1C* genes in 179 rice cultivars as predicted from their nucleotide sequence obtained by targeted re-sequencing**

**>Sub1A protein 1 for Haplotypes 3,4,5,6,7, 8 (Reference type)**MCGGEVIPADMPAAPFTPRHGDGETWVDRKRRNKKKRKRGADEEWEAAFQEFMAADDDDDGGGLVLSSKSLVLRSPGENDAGRGAAATMSMPLDPVTEEAEPAVAEKPRRRRPRRSYEYHGIRQRPWGRWSSEIRDPVKGVRLWLGTFDTAVEAALAYDAEARRIHGWKARTNFPPADLSSPPPPSQPLCFLLNDNGLITIGEAPTDDAASTSTSTTEASGDARIQLECCSDDVMDSLLAGYDVASGDDIWTWTSGASSTSVNQEIKTPSIHQNISYAGEA

>Sub1A protein 2 for Haplotypes 1 and 2
MCGGEVIPADMPAAPFTPRHGDGETWVDRKRRNKKKRKRGADEEWEAAFQEFMAADDDDDGGGLVLSSKSLVLRSPGENDAGRGAAATMSMPLDPVTEEAEPAVAEKPRRRRPRRSYEYHGIRQRPWGRWSSEIRDPVKGVRLWLGTFDTAVEAALAYDAEARRIHGWKARTNFPPADLSSPPPPPQPLCFLLNDNGLITIGEAPTDDAASTSTSTTEASGDARIQLECCSDDVMDSLLAGYDVASGDDIWTWTSGASSTSVNQEIKTPSIHQNISYAGEA

**>Sub1B protein 1 for Haplotype 1,2,3,5,6,7,8,9 (Reference type)**MCGGALIPNDYGDKPPPPPSESSEWDATTKMKKKKKRGGGGDDDWEAAFREFIAGDVDDDDDGVSMFPSGAGTMETTTEVAVVERPRRRRRVRRSYPYRGVRQRPWGRWASEIRDPVKGARVWLGTFDTAAEAARAYDAEARRIHGHKARTNFPPDEPPRPAPSQAPFCFLLDDDDDGVARGNSPASSSAPDSTSACTTSSTVASGERGDELILLECCSDDVMDSLLAGFDVSSESRSILGMVN

>Sub1B protein 2 for Haplotype 4
MCGGALIPNDYGDKPPPPPSESSEWDATTKMKKKKKRGGGGDDDWEAAFREFIAGDVDDDDDGVSMFPSGAGTMETTTEVAVVERPRRRRRVRWSYPYRGVRQRPWGRWASEIRDPVKGARVWLGTFDTAAEAARAYDAEARRIHGHKARTNFPPDEPPRPAPSQAPFCFLLDDDDDGVARGNSPASSSAPDSTSACTTSSTVASGERGDELILLECCSDDVMDSLLAGFDVSSESRSILGMVN

**>Sub1C protein 1 for haplotype 1 to 8 (Reference type)**

MRRRVSSSPSSSSSSSPARHHKARRSRRKLVADEDWEAAFREFLSRDDDDDDDDDDGHHVVVAPLIRSSNKCVHGHEVVASTVGGGASGGRRRADDDDGERRRRRRRERRSYPYRGIRQRPWGRWASEIRDPVKGIRVWLGTFDTAEGAARAYDDEVRRIYGGNAKTNFPPSPPPPEQPAAPVAAERSPSTTTTTTPSAEDSGDSRILIECCSDDLMDSLLAAFDMTTGDMRFWS

>Sub1C protein 2 for haplotype 9

MRRRVSSSPSSSSSSSPARHHKARRSRRKLVVDEDWEAAFREFLSRDDDDDDDDDDGQHVVVAPLIRGSDKCVHGHEVVASTVGGGASGGRRRADDDDGERRRRRRRERRSYPYRGIRQRPWGRWASEIRDPVKGIRVWLGTFDTAEGAARAYDDEVRRIYGGNAKTNFPPSPPPPEQPAAPVAAERSPSTTPPTTTSAEDSGDSRILIECFSDDLMDSLLAAFDMTTGDMRFWS

>Sub1C protein 3 for haplotype 10
MRRRVSSSSSSSSSSSPARHHKARRSRRKLAVDEDWEAAFREFLSRDDDDDDDDDDGQHVVVAPLIRGSDKCVHGHEVVASTVGGGASGGRRRADDDDGERRRRRRRERRSYPYRGIRQRPWGRWASEIRDPVKGIRVWLGTFDTAEGAARAYDDEVRRIYGGNAKTNFPPSPPPPEQPAAPVAAERSPSTTPPTTTEDSGDSRILIECFSDDLMDSLLAAFDMTTGDMRFWS

>Sub1C protein 4 for haplotype 11
MRRRVSSSSSSSSSSSPARHHKARRSRRKLAVDEDWEAAFREFLSRDHDDDDDDHDGQHVVVAPLIRGSDKCVHGHEVVASTVGGGASGGRRRADDDDGERRRRRRRERRSYPYRGIRQRPWGRWASEIRDPVKGIRVWLGTFDTAEGAARAYDDEVRRIYGGNAKTNFPPSPPPPEQPAAPVAAERSPSTTPPTTTEDSSDSRILIECFSDDLMDSLLAAFDMTTGDMRFWS

> Sub1C protein 5 for haplotype 12
MRRRVSSSSSSSSSSSPARHHKARRSRRKLAVDEDWEAAFREFLSRDHDDDDDDHDGQHVVVAPLIRGSDKCVHGHEVVASTVGGGASGGRRRADDDDGERRRRRRREKRSYPYRGIRQRPWGRWASEIRDPVKGIRVWLGTFDTAEGAARAYDDEVRRIYGGNAKTNFPPSPPPPEQPAAPVAAERSPSTTPPTTTEDSGDSRILIECFSDDLMDSLLAAFDMTTGDMRFWS

>Sub1C protein 6 for haplotype 13
MRRRVSSSSSSSPARHHKARRSRRKLAVDEDWEAAFREFLSRDHDDDDDDHDGQHVVVAPLIRGSDKCVHGHEVVASTVGGGASGGRRRADDDDGERRRRRRREKRSYPYRGIRQRPWGRWASEIRDPVKGIRVWLGTFDTAEGAARAYDDEVRRIYGGNAKTNFPPSPPPPEQPAAPVAAERSPSTTPPTTTEDSGDSRILIECCSDDLMDSLLAAFDMTTGDMRFWS

>Sub1C protein 7 for haplotype 14 MRRRVSSSSSSSSSSSPARHHKARRSRRKLAVDEDWEAAFREFLSRDHDDDDDHHDGQHVVVAPLIRGSDKCVHGHEVVASTVGGGASGGRRRADDDDGERRRRRRREKRSYPYRGIRQRPWGRWASEIRDPVKGIRVWLGTFDTAEGAARAYDDEVRRIYGGNAKTNFPPSPPPPEQPAAPVAAERSPSTTPPTTTEDSGDSRILIECFSDDLMDSLLAAFDMTTGDMRFWS

>Sub1C protein 8 for haplotype 15
MRRRVSSSSSSSSSSSPARHHKARRSRRKLAVDEDWEAAFREFLSRDHDDDDDDHDGQHVVVAPLIRGSDKCVHGHEVVASTVGGGASGGRRRADDDDGERRRRRRREKRSYPYRGIRQRPWGRWASEIRDPVKGIRVWLGTFDTAEGAARAYDDEVRRIYGGNAKTNFPPSPPPPEQPAAPVAAERSPSTTPPTTTEDSGDSRILIECFSDDLMDSLLAAFDMTTGDMRFWS

>Sub1C protein 9 for haplotype 16 MRRRVSSSSSSSSSSSPARHHKARRSRRKLAVDEDWEAAFREFLSRDHDDDDDDHDGQHVVVAPLIRGSDKCVHGHEVVASTVGGGASGGRRRADDDDGERRRRRRRERRSYPYRGIRQRPWGRWASEIRDPVKGIRVWLGTFDTAEGAARAYDDEVRRIYGGNAKTNFPPSPPPPEQPAAPVAAERSPSTTPPTTTEDSGDSRILIECFSDDLMDSLLAAFDMTTGDMRFWS

>Sub1C protein 10 for haplotype 18
MRRRVSSSSSSSSSSSPARHHKARRSRRKLAVDEDWEAAFREFLSRDHDDDDDDHDGQHVVVAPLIRGSDKCVHGHEVVASTVGGGASGGRRRADDDDGERRRRRRREKRSYPYRGIRQRPWGRWASEIRDPVKGIRVWLGTFDTAEGAARAYDDEVRRIYGGNAKTNFPPSPPPPEQPAAPVAAERSPSTTPPTTTEDSGDSRILIECFSDDLMDSLLAAFDMTTGDMRFWS

>Sub1C protein 11 for haplotype 19
MRRRVSSSSSSSSSSSPARHHKARRSRRKLAVDEDWEAAFREFLSRDHDDDDDDHDGQHVVVAPLIRGSDKCVHGHEVVASTVGGGASGGRRRADDDDGERRRRRRREKRSYPYRGIRQRPWGRWASEIRDPVKGIRVWLGTFDTAEGAARAYDDEVRRIYGGNAKTNFPPSPPPPEQPAAPVAAERSPSTTPPTTTEDSGDSRILIECCSDDLMDSLLAAFDMTTGDMRFWS

>Sub1C protein 12 for haplotypes 21,22,24,27,28,29,30,32,37,38,44,57,58,62,74,75,94,95,102,113,114,122,125,132,51
MRRRVSSSSSSSSSSSPARHHKARRSRRKLAVDEDWEAAFREFLSRDHDDDDDDHDGQHVVVAPLIRGSDKCVHGHEVVASTVGGGASGGRRRADDDDGERRRRRRREKRSYPYRGIRQRPWGRWASEIRDPVKGIRVWLGTFDTAEGAARAYDDEVRRIYGGNAKTNFPPSPPPPEQPAAPVAAERSPSTTPPTTTEDSGDSRILIECFSDDLMDSLLAAFDMTTGDMRFWS

>Sub1C protein 13 for haplotype 23,25,26,31,48,68,129,76,78,59,82,84,93,100,101,111,128,103,109,33
MRRRVSSSSSSSSSSSPARHHKARRSRRKLAVDEDWEAAFREFLSRDHDDDDDDHDGQHVVVAPLIRGSDKCVHGHEVVASTVGGGASGGRRRADDDDGERRRRRRREKRSYPYRGIRQRPWGRWASEIRDPVKGIRVWLGTFDTAEGAARAYDDEVRRIYGGNAKTNFPPSPPPPEQPAAPVAAERSPSTTPPTTTEDSGDSRILIECFSDDLMDSLLAAFDMTTGDMRFWS

>Sub1C protein 14 for haplotype 34
MRRRVSSSSSSSSSSSPARHHKARRSRRKLAVDEDWEAAFREFLSRDHDDDDDDHDGQHVVVAPLIRGSDKCVHGHEVVASTVGGGASGGRRRADDDDGERRRRRRRERRSYPYRGIRQRPWGRWASEIRDPVKGIRVWLGTFDTAEGAARAYDDEVRRIYGGNAKTNFPPSPPPPEQPAAPVAAERSPSTTPPTTTSAEDSGDSRILIECFSDDLMDSLLAAFDMTTGDMRFWS

>Sub1C protein 15 for haplotype 35,40,41,45,47,49,52,54,55,69,39
MRRRVSSSSSSSSSSSPARHHKARRSRRKLAVDEDWEAAFREFLSRDHDDDDDDHDGQHVVVAPLIRGSDKCVHGHEVVASTVGGGASGGRRRADDDDGERRRRRRREKRSYPYRGIRQRPWGRWASEIRDPVKGIRVWLGTFDTAEGAARAYDDEVRRIYGGNAKTNFPPSPPPPEQPAAPVAAERSPSTTPTTTTEDSGDSRILIECCSDDLMDSLLAAFDMTTGDMRFWS

>Sub1C protein 16 for haplotype 36 MRRRVSSSSSSSSSSSPARHHKARRSRRKLAVDEDWEAAFREFLSRDHDDDDDDHDGQHVVVAPLIRGSDKCVHGHEVVASTVGGGASGGRRRADDDDGERRRRRRREKRSYPYRGIRQRPWGRWASEIRDPVKGIRVWLGTFDTAEGAARAYDDEVRRIYGGNAKTNFPPSPPPPEQPAAPVAAERSPSTTPPTTTEDSGDSRILIECCSDDLMDSLLAAFDMTTGDMRFWS

>Sub1C protein 17 for haplotype 42
MRRRVSSSSSSSSSSSPARHHKARRSRRKLAVDEDWEAAFREFLSRDHDDDDDDHDGQHVVVAPLIRGSDKCVHGHEVVASTVGGGASGGRRRADDDDGERRRRRRREKRSYPYRGIRQRPWGRWASEIRDPVKGIRVWLGTFDTAEGAARAYDDEVRRIYGGNAKTNFPPSPPPPEQPAAPVAAERSPSTTPTTTTEDSGDSRILIECCSDDLMDSLLAAFDMTTGDMRFWS

>Sub1C protein 18 for haplotype 43
MRRRVSSSPSSSSSSSPARHHKARRSRRKLGADEDWEAAFREFLSRDYDDDDDDHDGHQVVVAPLIRCSDKCVHGHEVVASTVGGGASGGRRRADDDDGERRRRRRRERRSYPYRGIRQRPWGRWASEIRDPVKGIRVWLGTFDTAEGAARAYDDEVRRIYGGNAKTNFPPSPPPPEQPAAPVAAERSPSTTPTPTTEDSGDSRILIECCSDDLMDSLLAAFDMTTGDMRFWS

>Sub1C protein 19 for haplotype 46
MRRRVSSSSSSSSSPARHHKARRSRRKLAVDEDWEAAFREFLSRDHDDDDDDHDGQHVVVAPLIRGSDKCVHGHEVVASTVGGGASGGRRRADDDDGERRRRRRREKRSYPYRGIRQRPWGRWASEIRDPVKGIRVWLGTFDTAEGAARAYDDEVRRIYGGNAKTNFPPSPPPPEQPAAPVAAERSPSTTPTTTTEDSGDSRILIECCSDDLMDSLLAAFDMTTGDMRFWS

>Sub1C protein 20 for haplotype 66,64,67,71,80,81,83,85,88,90,91,96, 104,105,106,107,108,112,115,116,119,120,124,130,133,135,136,50
MRRRVSSSSSSSSSSSPARHHKARRSRRKLAVDEDWEAAFREFLSRDHDDDDDDHDGQHVVVAPLIRGSDKCVHGHEVVASTVGGGASGGRRRADDDDGERRRRWRREKRSYPYRGIRQRPWGRWASEIRDPVKGIRVWLGTFDTAEGAARAYDDEVRRIYGGNAKTNFPPSPPPPEQPAAPVAAERSPSTTPPTTTEDSSDSRILIECCSDDLMDSLLAAFDMTTGDMRFWS

>Sub1C protein 21 for haplotype 53,56
MRRRVSSSSSSSSSSSPARHHKARRSRRKLAVDEDWEAAFREFLSRDHDDDDDDHDGQHVVVAPLIRGSDKCVHGHEVVASTVGGGASGGRRRADDDDGERRRRRRREKRSYPYRGIRQRPWGRWASEIRDPVKGIRVWLGTFDTAEGAARAYDDEVRRIYGGNAKTNFPPSPPPPEQPAAPVAAERSPSTTPPTTTEDSGDSRILIECCSDDLMDSLLAAFDMTTGDMRFWS

>Sub1C protein 22 for haplotype 86,60
MRRRVSSSSSSSSSSSPARHHKARRSRRKLAVDEDWEAAFREFLSRDHDDDDDDHDGQHVVVAPLIRGSDKCVHGHEVVASTVGGGASGGRRRADDDDGERRRRWRREKRSYPYRGIRQRPWGRWASEIRDPVKGIRVWLGTFDTAEGAARAYDDEVRRIYGGNAKTNFPPSPPPPEQPAAPVAAERSPSTTPPTTTEDSGDSRILIECCSDDLMDSLLAAFDMTTGDMRFWS

>Sub1C protein 23 for haplotype 87,89,61
MRRRVSSSSSSSSSSSPARHHKARRSRRKLAVDEDWEAAFREFLSRDHDDDDDDHDGQHVVVAPLIRGSDKCVHGHEVVASTVGGGASGGRRRADDDDGERRRRWRREKRSYPYRGIRQRPWGRWASEIRDPVKGIRVWLGTFDTAEGAARAYDDEVRRIYGGNAKTNFPPSPPPPEQPAAPVAAERSPSTTPPTTTEDSGDSRILIECCSDDLMDSLLAAFDMTTGDMRFWS

>Sub1C protein 24 for haplotype 77,63
MRRRVSSSSSSSSSSSPARHHKARRSRRKLAVDEDWEAAFREFLSRDHDDDDDDHDGQHVVVAPLIRGSDKCVHGHEVVASTVGGGASGGRRRADDDDGERRRRRRREKRSYPYRGIRQRPWGRWASEIRDPVKGIRVWLGTFDTAEGAARAYDDEVRRIYGGNAKTNFPPSPPPPEQPAAPVAAERSPSTTPPTTTEDSGDSRILIECFSDDLMDSLLAAFDMTTGDMRFWS

>Sub1C protein 25 for haplotype 79,65
MRRRVSSSSSSSSSSSPARHHKARRSRRKLAVDEDWEAAFREFLSRDHDDDDDDHDGQHVVVAPLIRGSDKCVHGHEVVASTVGGGASGGRRRADDDDGERRRRWRREKRSYPYRGIRQRPWGRWASEIRDPVKGIRVWLGTFDTAEGAARAYDDEVRRIYGGNAKTNFPPSPPPPEQPAAPVAAERSPSTTPTTTTEDSSDSRILIECCSDDLMDSLLAAFDMTTGDMRFWS

>Sub1C protein 26 for haplotype 70
MRRRVSSSSSSSSSSSPARHHKARRSRRKLAVDEDWEAAFREFLSRDDDDDDDDDDGQHVVVAPLIRGSDKCVHGHEVVASTVGGGASGGRRRADDDDGERRRRRRREKRSYPYRGIRQRPWGRWASEIRDPVKGIRVWLGTFDTAEGAARAYDDEVRRIYGGNAKTNFPPSPPPPEQPAAPVAAERSPSTTPPTTTEDSGDSRILIECFSDDLMDSLLAAFDMTTGDMRFWS

>Sub1C protein 27 for haplotype 97,99,110,131,137,139,140,72
MRRRVSSSSSSSSSSSPARHHKARRSRRKLAVDEDWEAAFREFLSRDHDDDDDDHDGQHVVVAPLIRGSDKCVHGHEVVASTVGGGASGGRRRADDDDGERRRRWRREKRSYPYRGIRQRPWGRWASEIRDPVKGIRVWLGTFDTAEGAARAYDDEVRRIYGGNAKTNFPPSPPPPEQPAAPVAAERSPSTTPPTTTEDSSDSRILIECCSDDLMDSLLAAFDMTTGDMRFWS

>Sub1C protein 28 for haplotype 73,92
MRRRVSSSSSSSSSSSPARHHKARRSRRKLAVDEDWEAAFREFLSRDHDDDDDDHDGQHVVVAPLIRGSDKCVHGHEVVASTVGGGASGGRRRADDDDGERRRRRRREKRSYPYRGIRQRPWGRWASEIRDPVKGIRVWLGTFDTAEGAARAYDDEVRRIYGGNAKTNFPPSPPPPEQPAAPVAAERSPSTTPPTTTEDSGDSRILIECFSDDLMDSLLAAFDMTTGDMRFWS

>Sub1C protein 29 for haplotype 98
MRRRVSSSSSSSSSSSPARHHKARRSRRKLAVDEDWEAAFREFLSRDHDDDDDDHDGQHVVVAPLIRGSDKCVHGHEVVASTVGGGASGGRRRADDDDGERRRRRRREKRSYPYRGIRQRPWGRWASEIRDPVKGIRVWLGTFDTAEGAARAYDDEVRRIYGGNAKTNFPPSPPPPEQPAAPVAAERSPSTTTPTPTEDSGDSRILIECCSDDLMDSLLAAFDMTTGDMRFWS

>Sub1C protein 30 for haplotype 138
MRRRVSSSSSSSSSSSPARHHKARRSRRKLAVDEDWEAAFREFLSRDHDDDDDDHDGQHVVVAPLIRGSDKCVHGHEVVASTVGGGASGGRRRADDDDGERRRRRRREKRSYPYRGIRQRPWGRWASEIRDPVKGIRVWLGTFDTAEGAARAYDDEVRRIYGGNAKTNFPPSPPPPEQPAAPVAAERSPSTTPPTTTEDSGDSRILIECCSDDLMDSLLAAFDMTTGDMRFWS

>Sub1C protein 31 for haplotype 117
MRRRVSSSSSSSSSSSPARHHKARRSRRKLAVDEDWEAAFREFLSRDHDDDDDDHDGQHVVVAPLIRGSDKCVHGHEVVASTVGGGASGGRRRADDDDGERRRRRRREKRSYPYRGIRQRPWGRWASEIRDPVKGIRVWLGTFDTAEGAARAYDDEVRRIYGGNAKTNFPPSPPPPEQPAAPVAAERSPSTTPPTTTEDSGDSRILIECCSDDLMDSLLAAFDMTTGDMRFWS

>Sub1C protein 32 for haplotype 127
MRRRVSSSSSSSSSSSPARHHKARRSRRKLAVDEDWEAAFREFLSRDHDDDDDDHDGQHVVVAPLIRGSDKCVHGHEVVASTVGGGASGGRRRADDDDGERRRRRRREKRSYPYRGIRQRPWGRWASEIRDPVKGICVWLGTFDTAEGAARAYDDEVRRIYGGNAKTNFPPSPPPPEQPAAPVAAERSPSTTPPTTTEDSGDSRILIECCSDDLMDSLLAAFDMTTGDMRFWS

>Sub1C protein 33 for haplotype 118
MRRRVSSSSSSSSSSSPARHHKARRSRRKLAVDEDWEAAFREFLSRDHDDDDDDHDGQHVVVAPLIRGSDKCVHGHEVVASTVGGGASGGRRRADDDDGERRRRWRREKRSYPYRGIRQRPWGRWASEIRDPVKGIRVWLGTFDTAEGAARAYDDEVRRIYGGNAKTNFPPSPPPPEQPAAPVAAERSPSTTPPTPTEDSSDSRILIECCSDDLMDSLLAAFDMTTGDMRFWS

>Sub1C protein 34 for haplotype 126
MRRRVSSSSSSSSSSSPARHHKARRSRRKLAVDEDWEAAFREFLSRDHDDDDDDHDGQHVVVAPLIRGSDKCVHGHEVVASTVGGGASGGRRRADDDDGERRRRWRREKRSYPYRGIRQRPWGRWASEIRDPVKGIRVWLGTFDTAEGAARAYDDEVRRIYGGNAKTNFPPSPPPPEQPAAPVAAERSPSTTPPTPTEDSSDSRILIECCSDDLMDSLLAAFDMTTGDMRFWS

>Sub1C protein 35 for haplotype 123
MRRRVSSSSSSSSSSSPARHHKARRSRRKLAVDEDWEAAFREFLSRDHDDDDDDHDGQHVVVAPLIRGSDKCVHGHEVVASTVGGGASGGRRRADDDDGERRRRRRREKRSYPYRGIRQRPWGRWASEIRDPVKGIRVWLGTFDTAEGAARAYDDEVRRIYGGNAKTNFPPSPPPPEQPAAPVAAERSPSTTPPTPTEDSGDSRILIECFSDDLMDSLLAAFDMTTGDMRFWS

>Sub1C protein 36 for haplotype 134
MRRRVSSSSSSSSSSSPARHHKARRSRRKLAVDEDWEAAFREFLSRDHDDDDDDHDGQHVVVAPLIRGSDKCVHGHEVVASTVGGGASGGRRRADDDDGERRRRWRREKRSYPYRGIRQRPWGRWASEIRDPVKGIRVWLGTFDTAEGAARAYDDEVRRIYGGNAKTNFPPSPPPPEQPAAPVAAERSPSTTPPTTTEDSSDSRILIECFSDDLMDSLLAAFDMTTGDMRFWS

**Table S10.** Survival of plants of 165 rice cultivars after 15 days of vegetative stage submergence at IRRI, Philippines in 2012. Variable numbers of seedlings (up to 20) in large trays were subjected to flooding in a concrete submergence tank and data recorded 21 days after desubmergence.

| **Sr. no.** | **Variety** | **No. of seedlings planted Rep1** | **No. of seedlings survived Rep1** | **% Survival rep 1** | **No of seedlings planted Rep 2** | **No of seedlings planted Rep 2** | **% Survival Rep 2** | **% Survival Mean** |
| --- | --- | --- | --- | --- | --- | --- | --- | --- |
|  | FR 13A | 20 | 20 | 100 | 20 | 20 | 100 | **100** |
|  | IR-36 | 19 | 19 | 100 | 18 | 18 | 100 | **100** |
|  | MAHA DIKWEE | 20 | 19 | 95 | 17 | 17 | 100 | **97.5** |
|  | KAJARAHAWA | 16 | 15 | 94 | 18 | 18 | 100 | **97** |
|  | DHOLAMON 64-3 | 20 | 18 | 90 | 18 | 18 | 100 | **95** |
|  | JAHABHOG | 19 | 18 | 95 | 20 | 19 | 95 | **95** |
|  | PATAIM | 20 | 20 | 100 | 21 | 19 | 90 | **95** |
|  | ARC 614 | 19 | 17 | 89 | 20 | 20 | 100 | **94.5** |
|  | Dihawan | 20 | 20 | 100 | 18 | 16 | 89 | **94.5** |
|  | IR 64 | 20 | 20 | 100 | 19 | 17 | 89 | **94.5** |
|  | FR 43B | 21 | 20 | 95 | 22 | 20 | 91 | **93** |
|  | KATY | 22 | 19 | 86 | 20 | 20 | 100 | **93** |
|  | MEGHI | 22 | 19 | 86 | 19 | 19 | 100 | **93** |
|  | KAMNAM | 19 | 18 | 95 | 20 | 18 | 90 | **92.5** |
|  | KARNAL LOCAL | 20 | 19 | 95 | 20 | 18 | 90 | **92.5** |
|  | NCS 348 | 20 | 18 | 90 | 20 | 19 | 95 | **92.5** |
|  | S-155 | 21 | 19 | 90 | 20 | 19 | 95 | **92.5** |
|  | SINGHARA | 20 | 17 | 85 | 20 | 20 | 100 | **92.5** |
|  | KALUWEE | 19 | 16 | 84 | 20 | 20 | 100 | **92** |
|  | KHAIYAN | 18 | 16 | 89 | 19 | 18 | 95 | **92** |
|  | S-141 | 20 | 19 | 95 | 19 | 17 | 89 | **92** |
|  | SWARNA | 19 | 17 | 89 | 19 | 18 | 95 | **92** |
|  | RAJBHOG | 18 | 15 | 83 | 16 | 16 | 100 | **91.5** |
|  | SIPULUT PANDAN | 20 | 16 | 80 | 18 | 18 | 100 | **90** |
|  | ASAMIA | 21 | 20 | 95 | 19 | 16 | 84 | **89.5** |
|  | MADHUKAR | 20 | 18 | 90 | 19 | 17 | 89 | **89.5** |
|  | KOTTAMALI | 9 | 9 | 100 | 18 | 14 | 78 | **89** |
|  | SUGAPANKH | 18 | 15 | 83 | 20 | 19 | 95 | **89** |
|  | FRG1 | 23 | 22 | 96 | 20 | 16 | 80 | **88** |
|  | TAGBA | 20 | 16 | 80 | 20 | 19 | 95 | **87.5** |
|  | S-138 | 19 | 16 | 84 | 20 | 18 | 90 | **87** |
|  | HOURA KANI | 19 | 17 | 89 | 19 | 16 | 84 | **86.5** |
|  | ARC 12172 | 18 | 18 | 100 | 18 | 13 | 72 | **86** |
|  | BABARAKSHA KARO | 21 | 18 | 86 | 20 | 17 | 85 | **85.5** |
|  | S-148 | 20 | 20 | 100 | 21 | 15 | 71 | **85.5** |
|  | TETEP | 19 | 18 | 95 | 21 | 16 | 76 | **85.5** |
|  | GODA HEENATI | 20 | 16 | 80 | 20 | 18 | 90 | **85** |
|  | SURAHA | 19 | 16 | 84 | 21 | 18 | 86 | **85** |
|  | CHUDI | 18 | 17 | 94 | 20 | 15 | 75 | **84.5** |
|  | Sambha-Sub1 | 23 | 23 | 100 | 21 | 14 | 67 | **83.5** |
|  | MADAL | 21 | 18 | 86 | 20 | 16 | 80 | **83** |
|  | SAVITRI | 20 | 19 | 95 | 21 | 15 | 71 | **83** |
|  | SWARNA-SUB1 | 21 | 18 | 86 | 20 | 16 | 80 | **83** |
|  | BARA SALI | 19 | 19 | 100 | 20 | 13 | 65 | **82.5** |
|  | KARABONKA | 20 | 15 | 75 | 20 | 18 | 90 | **82.5** |
|  | LEMONT | 24 | 23 | 96 | 16 | 11 | 69 | **82.5** |
|  | PANKEI | 19 | 16 | 84 | 19 | 15 | 79 | **81.5** |
|  | SANG KHLA | 22 | 15 | 68 | 19 | 18 | 95 | **81.5** |
|  | SITA MATA | 20 | 16 | 80 | 22 | 18 | 82 | **81** |
|  | CHANGPHAI | 20 | 17 | 85 | 19 | 14 | 74 | **79.5** |
|  | MOTI | 20 | 16 | 80 | 19 | 15 | 79 | **79.5** |
|  | JOHAINEANG(WET ) | 20 | 18 | 90 | 21 | 14 | 67 | **78.5** |
|  | KOLA JOHA | 18 | 15 | 83 | 19 | 14 | 74 | **78.5** |
|  | KRETEK SIRENDAH MERAH | 18 | 16 | 89 | 15 | 10 | 67 | **78** |
|  | PAIAM | 21 | 17 | 81 | 20 | 15 | 75 | **78** |
|  | LATSIKA | 20 | 18 | 90 | 20 | 13 | 65 | **77.5** |
|  | TATAN | 20 | 18 | 90 | 20 | 13 | 65 | **77.5** |
|  | LABELLE | 19 | 15 | 79 | 19 | 14 | 74 | **76.5** |
|  | PAYEMA | 21 | 13 | 62 | 20 | 18 | 90 | **76** |
|  | S-177 | 20 | 16 | 80 | 20 | 14 | 70 | **75** |
|  | S-183 | 20 | 16 | 80 | 20 | 14 | 70 | **75** |
|  | SOSSOKA | 14 | 11 | 79 | 17 | 12 | 71 | **75** |
|  | TAH TONE | 20 | 17 | 85 | 17 | 11 | 65 | **75** |
|  | GOTHAWA | 17 | 16 | 94 | 20 | 11 | 55 | **74.5** |
|  | PANNEL | 19 | 12 | 63 | 20 | 17 | 85 | **74** |
|  | PANIDUBI | 20 | 13 | 65 | 16 | 13 | 81 | **73** |
|  | BAGI BARDHANA | 20 | 11 | 55 | 20 | 18 | 90 | **72.5** |
|  | CR 1009 | 23 | 15 | 65 | 20 | 16 | 80 | **72.5** |
|  | PALENEMA | 20 | 18 | 90 | 25 | 13 | 52 | **71** |
|  | BARA RANGA | 20 | 12 | 60 | 21 | 17 | 81 | **70.5** |
|  | BUNTOK | 20 | 13 | 65 | 20 | 15 | 75 | **70** |
|  | CHULTA BALUNGA (WILD) | 20 | 12 | 60 | 20 | 16 | 80 | **70** |
|  | DULAR | 20 | 12 | 60 | 20 | 16 | 80 | **70** |
|  | HSUNG TIENG | 20 | 12 | 60 | 20 | 16 | 80 | **70** |
|  | MANOHAR SALI | 20 | 9 | 45 | 19 | 18 | 95 | **70** |
|  | TSAO WAN CHING | 17 | 9 | 53 | 15 | 13 | 87 | **70** |
|  | CHANGMAKA | 19 | 14 | 74 | 20 | 13 | 65 | **69.5** |
|  | IR 72 | 19 | 9 | 47 | 18 | 16 | 89 | **68** |
|  | BAD JHARLI | 19 | 14 | 74 | 18 | 11 | 61 | **67.5** |
|  | BINULAWAN | 20 | 12 | 60 | 20 | 15 | 75 | **67.5** |
|  | CHANG MUI | 20 | 14 | 70 | 20 | 13 | 65 | **67.5** |
|  | KARKATI 87 | 18 | 12 | 67 | 19 | 13 | 68 | **67.5** |
|  | MOUSSAYA | 20 | 18 | 90 | 20 | 9 | 45 | **67.5** |
|  | ASHRA LAKI | 24 | 22 | 92 | 19 | 8 | 42 | **67** |
|  | NAALUMOLI KARUPPAN | 14 | 7 | 50 | 19 | 16 | 84 | **67** |
|  | SATHWA | 20 | 18 | 90 | 21 | 9 | 43 | **66.5** |
|  | IC 370811 | 19 | 17 | 89 | 14 | 6 | 43 | **66** |
|  | PANI DHAN 2 | 21 | 11 | 52 | 20 | 16 | 80 | **66** |
|  | KALONCHI | 19 | 13 | 68 | 19 | 12 | 63 | **65.5** |
|  | NIRBOI | 18 | 10 | 56 | 20 | 15 | 75 | **65.5** |
|  | VANAN VELLAI | 20 | 12 | 60 | 17 | 12 | 71 | **65.5** |
|  | CHUMANSAL-39 | 20 | 15 | 75 | 20 | 11 | 55 | **65** |
|  | DUDHA LADU | 20 | 8 | 40 | 20 | 18 | 90 | **65** |
|  | EMPING ARA | 20 | 13 | 65 | 20 | 13 | 65 | **65** |
|  | HEEN SULAI | 20 | 9 | 45 | 20 | 17 | 85 | **65** |
|  | MEGHA BADALA | 20 | 9 | 45 | 20 | 17 | 85 | **65** |
|  | PANIKAKUA | 20 | 17 | 85 | 20 | 9 | 45 | **65** |
|  | ASANA | 22 | 17 | 77 | 21 | 11 | 52 | **64.5** |
|  | M202 | 17 | 10 | 59 | 19 | 13 | 68 | **63.5** |
|  | ASHU BHAJNA | 21 | 10 | 48 | 18 | 14 | 78 | **63** |
|  | KHARSU 80A: Oryza sp | 19 | 11 | 58 | 19 | 13 | 68 | **63** |
|  | TIEBIMAH | 20 | 16 | 80 | 20 | 9 | 45 | **62.5** |
|  | BIRPALA | 20 | 8 | 40 | 19 | 16 | 84 | **62** |
|  | CHAKIA 59 | 22 | 19 | 86 | 21 | 8 | 38 | **62** |
|  | KASALATH | 19 | 13 | 68 | 20 | 11 | 55 | **61.5** |
|  | ZOBGUI | 20 | 12 | 60 | 19 | 12 | 63 | **61.5** |
|  | AUSBORO | 15 | 7 | 47 | 20 | 15 | 75 | **61** |
|  | PAKRIBUNA | 20 | 10 | 50 | 20 | 14 | 70 | **60** |
|  | AUS 287 | 20 | 7 | 35 | 20 | 16 | 80 | **57.5** |
|  | JALKANTHI | 20 | 10 | 50 | 20 | 13 | 65 | **57.5** |
|  | LUNISHREE | 25 | 19 | 76 | 18 | 7 | 39 | **57.5** |
|  | NALDAK | 18 | 9 | 50 | 20 | 13 | 65 | **57.5** |
|  | KARIYWA | 20 | 15 | 75 | 18 | 7 | 39 | **57** |
|  | MEGHARAJ | 21 | 7 | 33 | 20 | 16 | 80 | **56.5** |
|  | VADAI | 20 | 7 | 35 | 18 | 14 | 78 | **56.5** |
|  | USA BATAPOLA AL | 18 | 10 | 56 | 20 | 11 | 55 | **55.5** |
|  | LEMO | 19 | 9 | 47 | 19 | 12 | 63 | **55** |
|  | PANIKEKEUA | 20 | 7 | 35 | 20 | 15 | 75 | **55** |
|  | PANSANKO | 21 | 14 | 67 | 19 | 8 | 42 | **54.5** |
|  | SANTI BAHN | 20 | 14 | 70 | 18 | 7 | 39 | **54.5** |
|  | Jalnidhi | 20 | 14 | 70 | 21 | 8 | 38 | **54** |
|  | MENYARHUNEI | 17 | 15 | 88 | 20 | 4 | 20 | **54** |
|  | JOKHRU | 20 | 15 | 75 | 19 | 6 | 32 | **53.5** |
|  | KALA BUNDE | 20 | 12 | 60 | 17 | 8 | 47 | **53.5** |
|  | JAGANATHA BALLAVA | 20 | 5 | 25 | 20 | 16 | 80 | **52.5** |
|  | THUNMAR HAMARA | 20 | 15 | 75 | 20 | 6 | 30 | **52.5** |
|  | BAD GEDE | 22 | 7 | 32 | 18 | 13 | 72 | **52** |
|  | ASAMIRUPA | 20 | 14 | 70 | 20 | 6 | 30 | **50** |
|  | BR11 | 20 | 9 | 45 | 20 | 11 | 55 | **50** |
|  | JAYMIS | 21 | 14 | 67 | 20 | 6 | 30 | **48.5** |
|  | MANDIRA | 20 | 7 | 35 | 21 | 13 | 62 | **48.5** |
|  | YEIKOYON | 19 | 10 | 53 | 20 | 8 | 40 | **46.5** |
|  | BAD DHANA | 18 | 8 | 44 | 23 | 11 | 48 | **46** |
|  | JC1 | 20 | 7 | 35 | 14 | 8 | 57 | **46** |
|  | BARACHIRAMARA | 20 | 6 | 30 | 20 | 12 | 60 | **45** |
|  | IARI 5825 | 21 | 12 | 57 | 21 | 7 | 33 | **45** |
|  | WAHNG | 20 | 12 | 60 | 20 | 6 | 30 | **45** |
|  | BABAI LACHHA | 20 | 10 | 50 | 18 | 7 | 39 | **44.5** |
|  | POKKALI | 24 | 17 | 71 | 17 | 3 | 18 | **44.5** |
|  | KAOLACK | 20 | 10 | 50 | 21 | 8 | 38 | **44** |
|  | AI CHIH AI KWOH 2 | 18 | 8 | 44 | 19 | 8 | 42 | **43** |
|  | DUB GELONG | 7 | 2 | 29 | 13 | 7 | 54 | **41.5** |
|  | JALDUNGI | 21 | 10 | 48 | 20 | 7 | 35 | **41.5** |
|  | CHANGMAN | 20 | 12 | 60 | 19 | 4 | 21 | **40.5** |
|  | MA-ZHAN(RED) | 24 | 7 | 29 | 21 | 11 | 52 | **40.5** |
|  | CHOW HA LA | 20 | 9 | 45 | 20 | 7 | 35 | **40** |
|  | CHUKKANNA KUMBALOAN | 20 | 4 | 20 | 19 | 11 | 58 | **39** |
|  | MARS | 19 | 9 | 47 | 20 | 6 | 30 | **38.5** |
|  | IR 42 | 21 | 8 | 38 | 19 | 6 | 32 | **35** |
|  | KURUWEE | 18 | 6 | 33 | 19 | 7 | 37 | **35** |
|  | MAINTIMOLOTSY | 17 | 7 | 41 | 18 | 5 | 28 | **34.5** |
|  | KHAO MINE LAR | 21 | 9 | 43 | 20 | 5 | 25 | **34** |
|  | BAGA GOHA | 18 | 12 | 67 | 20 | 0 | 0 | **33.5** |
|  | JADHAN | 20 | 9 | 45 | 17 | 3 | 18 | **31.5** |
|  | KHAO HLAN ON | 20 | 5 | 25 | 20 | 7 | 35 | **30** |
|  | JALMAGNA | 4 | 2 | 50 | 12 | 1 | 8.3 | **29.15** |
|  | CHASING | 18 | 0 | 0 | 19 | 11 | 58 | **29** |
|  | RENIK BATU | 16 | 3 | 19 | 15 | 5 | 33 | **26** |
|  | PECALO | 19 | 3 | 16 | 20 | 6 | 30 | **23** |
|  | AZUCENA | 19 | 7 | 37 | 19 | 0 | 0 | **18.5** |
|  | TUNDAHIYA | 20 | 1 | 5 | 20 | 6 | 30 | **17.5** |
|  | ROM RUNTIK | 19 | 6 | 32 | 12 | 0 | 0 | **16** |
|  | VAN | 20 | 2 | 10 | 21 | 3 | 14 | **12** |
|  | LIU-TIAO-NU | 20 | 4 | 20 | 18 | 0 | 0 | **10** |
|  | MOROBEREKAN | 20 | 2 | 10 | 21 | 2 | 9.5 | **9.75** |

**Population mean: 65.9%**

**Population range: 9.8% to100%**

**Table S11.** Survival of rice plants after 15 days of submergence in a pond at NDUAT, Ayodhya, India during 2012. Total 14 well-established plants one month after transplanting were subjected to submergence stress. Elongating type cultivars were either left intact (with canopy) or cut from the top (without canopy) to prevent air contact.

| **Sr. no.** | **Variety** | **No. survived with canopy** | **% Survial with canopy** | **No. survived without canopy** | **% Survival without canopy** | **Remarks** |
| --- | --- | --- | --- | --- | --- | --- |
| 1. | ARC 614 | 13 | **92.9** | 9 | **64.3** | Elongation |
| 2. | ASAMIA | 9 | **64.3** | 0 | **0.0** | Escape |
| 3. | ASANA | 0 | **0.0** | 0 | **0.0** | Elongated |
| 4. | ASHU BHAJNA | 4 | **28.6** | 2 | **14.3** | Poor recovery |
| 5. | AUSBORO | 14 | **100** | 0 | **0.0** | Escape |
| 6. | AZUCENA | 10 | **71.4** | 3 | **21.4** | Susceptible |
| 7. | BABAI LACHHA | 9 | **64.3** | 5 | **35.7** | Elongation |
| 9. | BAD DHANA | 7 | **50.0** | 5 | **35.7** | Poor recovery |
| 10. | BAD GEDE | 0 | **0.0** | 0 | **0.0** | Poor recovery |
| 11. | BAD JHARLI | 14 | **100** | 0 | **0.0** | Good |
| 12. | BAGA GOHA | 5 | **35.7** | 0 | **0.0** | Escape |
| 13. | BAGI BARDHANA | 9 | **64.3** | 0 | **0.0** | Regeneration |
| 14. | BARA RANGA | 9 | **64.3** | 9 | **64.3** | Regeneration |
| 15. | BARA SALI | 2 | **14.3** | 0 | **0.0** | Escape |
| 16. | BARACHIRAMARA | 1 | **7.1** | 1 | **7.1** | Susceptible |
| 17. | BINULAWAN | 6 | **42.9** | 4 | **28.6** | Old leaves die |
| 18. | BIRPALA | 13 | **92.9** | 4 | **28.6** | 20% leaves die |
| 19. | BR11 | 13 | **92.9** | 10 | **71.4** | Tiller mortality |
| 20. | BUNTOK | 14 | **100.0** | 13 | **92.9** | Regeneration |
| 21. | CHAKIA 59 | 0 | **0** | 0 | **0** | Poor |
| 22. | CHANG MUI | 13 | **92.9** | 6 | **42.9** | Elongated, escape |
| 23. | CHANGMAKA | 9 | **64.3** | 9 | **64.3** | Poor |
| 24. | CHANGMAN | 5 | **35.7** | 1 | **7.1** | Escape |
| 25. | CHOW HA LA | 10 | **71.4** | 6 | **42.9** | Regeneration |
| 26. | CHUDI | 11 | **78.6** | 4 | **28.6** |  |
| 27. | CHUKKANNA KUMBALOAN | 9 | **64.3** | 7 | **50.0** |  |
| 28. | CHULTA BALUNGA (WILD) | 4 | **28.6** | 2 | **14.3** |  |
| 29. | CHUMANSAL-39 | 10 | **71.4** | 8 | **57.1** | Regeneration |
| 30. | CR 1009 | 13 | **92.9** | 0 | **0.0** | Elongation |
| 31. | DHOLAMON 64-3 | 14 | **100.0** | 9 | **64.3** |  |
| 32. | DUDHA LADU | 9 | **64.3** | 10 | **71.4** | Escape |
| 33. | DULAR | 5 | **35.7** | 4 | **28.6** | 20% leaves |
| 34. | EMPING ARA | 10 | **71.4** | 6 | **42.9** | Regeneration |
| 35. | FR 13A | 13 | **92.9** | 13 | **92.9** |  |
| 36. | FR 43B | 13 | **92.9** | 13 | **92.9** |  |
| 37. | FRG1 | 0 | **0.0** | 0 | **0.0** |  |
| 38. | GODA HEENATI | 11 | **78.6** | 5 | **35.7** | 60% leaves damage |
| 39. | GOTA | 0 | **0.0** | 0 | **0.0** |  |
| 40. | GOTHAWA | 13 | **92.9** | 11 | **78.6** | Regeneration |
| 41. | HEEN SULAI | 12 | **85.7** | 9 | **64.3** | Regeneration |
| 42. | HOURA KANI | 13 | **92.9** | 1 | **7.1** | Tips damaged |
| 43. | HSUNG TIENG | 13 | **92.9** | 9 | **64.3** | Regeneration |
| 44. | IC370811 | 0 | **0.0** | 0 | **0.0** | Escape |
| 45. | IR 36 | 3 | **21.4** | 0 | **0.0** | Susceptible |
| 46. | IR 42 | 0 | **0.0** | 0 | **0.0** |  |
| 47. | IR-73707-45-3-23 | 0 | **0.0** | 0 | **0.0** |  |
| 48. | JAGANATHA BALLAVA | 11 | **78.6** | 4 | **28.6** | Good recovery |
| 49. | JAHABHOG | 12 | **85.7** | 3 | **21.4** | Escape |
| 50. | JALDUNGI | 14 | **100** | 7 | **50.0** | Escape |
| 51. | JALKANTHI | 9 | **64.3** | 3 | **21.4** | Poor recovery |
| 52. | Jalnidhi | 14 | **100** | 11 | **78.6** | Elongated |
| 53. | JAYMIS | 12 | **85.7** | 3 | **21.4** | Tips die |
| 54. | JC1 | 7 | **50.0** | 5 | **35.7** | Regeneration |
| 55. | JOHAINEANG (WET) | 0 | **0.0** | 0 | **0.0** | Escape |
| 56. | JOKHRU | 7 | **50.0** | 0 | **0.0** | Escape, lodging |
| 57. | KAJARAHAWA | 14 | **100.0** | 12 | **85.7** | Escape |
| 58. | KALA BUNDE | 7 | **50.0** | 7 | **50.0** | Poor |
| 59. | KALONCHI | 0 | **0.0** | 0 | **0.0** |  |
| 60. | KALUWEE | 12 | **85.7** | 8 | **57.1** | Tiller mortality |
| 61. | KAOLACK | 10 | **71.4** | 1 | **7.1** | Escape |
| 62. | KARABONKA | 8 | **57.1** | 0 | **0.0** | Regeneration |
| 63. | KARIYAWA | 13 | **92.9** | 9 | **64.3** | Escape |
| 64. | KARKATI 87 | 13 | **92.9** | 6 | **42.9** | Elongation |
| 65. | KARNAL LOCAL | 13 | **92.9** | 8 | **57.1** | Elongated lodging |
| 66. | KASALATH | 13 | **92.9** | 0 | **0.0** | Escape |
| 67. | KHAIYAN | 11 | **78.6** | 8 | **57.1** | Apex damaged |
| 68. | KHAO HLAN ON | 12 | **85.7** | 7 | **50.0** | Early, elongation |
| 69. | KOLA JOHA | 8 | **57.1** | 8 | **57.1** | Narrow leaves |
| 70. | KRETEK SIRENDAH MERAH | 7 | **50.0** | 5 | **35.7** | Susceptible |
| 71. | LATSIKA | 7 | **50.0** | 1 | **7.1** | Early flowering , escape |
| 72. | LIU-TIAO-NU | 12 | **85.7** | 6 | **42.9** | Apex damaged |
| 73. | LUNISHREE | 1 | **7.1** | 0 | **0.0** | Good recovery |
| 74. | MA-ZHAN(RED) | 14 | **100** | 1 | **7.1** | Escape |
| 75. | MADAL | 13 | **92.9** | 7 | **50.0** | Older leaves die |
| 76. | MAHA DIKWEE | 13 | **92.9** | 6 | **42.9** | Escape |
| 77. | MANOHAR SALI | 10 | **71.4** | 3 | **21.4** |  |
| 78. | MARS | 12 | **85.7** | 2 | **14.3** | Escape |
| 79. | MEGHARAJ | 11 | **78.6** | 0 | **0.0** | Escape |
| 80. | MEGHI | 7 | **50.0** | 0 | **0.0** | Escape, lodging |
| 81. | MENYAR HUNEI | 12 | **85.7** | 5 | **35.7** | Regeneration |
| 82. | MOTI | 13 | **92.9** | 0 | **0.0** | Regeneration |
| 83. | MOUSSAYA | 14 | **100.0** | 2 | **14.3** | Escape |
| 84. | NAALUMOLI KARUPPAN | 13 | **92.9** | 13 | **92.9** | 70% tips die |
| 85. | NALDAK | 14 | **100.0** | 11 | **78.6** | Tips damaged |
| 86. | NCS 348 | 6 | **42.9** | 4 | **28.6** | Escape |
| 87. | NIRBOI | 14 | **100** | 9 | **64.3** | Escape |
| 88. | PAIAIM | 14 | **100** | 11 | **78.6** | Elongated |
| 89. | PAKRIBUNA | 13 | **92.9** | 7 | **50.0** | Escape |
| 90. | PALENEMA | 11 | **78.6** | 0 | **0.0** |  |
| 91. | PALIANDEH | 0 | **0.0** | 0 | **0.0** |  |
| 92. | PANI DHAN 2 | 6 | **42.9** | 0 | **0.0** | Elongated, poor recovery |
| 93. | PANIDUBI | 11 | **78.6** | 9 | **64.3** | Escape |
| 94. | PANIKAKUA | 11 | **78.6** | Nil | **0.0** | Regeneration |
| 95. | PANIKEKEUA | 14 | **100.0** | 0 | **0.0** | Elongation |
| 96. | PANKEI | 14 | **100.0** | 3 | **21.4** | Escape |
| 97. | PANNEL | 14 | **100.0** | 0 | **0.0** |  |
| 99. | PATAIM | 14 | **100.0** | 0 | **0.0** | Regeneration |
| 101. | PECALO | 13 | **92.9** | 8 | **57.1** | Escape |
| 102. | RAJBHOG | 13 | **92.9** | 13 | **92.9** | Regeneration |
| 103. | RENIK BATU | 6 | **42.9** | 0 | **0.0** | Regeneration |
| 104. | ROM RUNTIK | 12 | **85.7** | 6 | **42.9** | Escape |
| 105. | S-138 | 12 | **85.7** | 9 | **64.3** | Good, short |
| 106. | S-141 | 11 | **78.6** | 9 | **64.3** | Old leaves die |
| 107. | S-148 | 10 | **71.4** | 0 | **0** | Escape |
| 108. | S-155 | 11 | **78.6** | 11 | **78.6** | Leaves green |
| 109. | S-177 | 10 | **71.4** | 9 | **64.3** | Poor regeneration |
| 110. | S-183 | 11 | **78.6** | 0 | **0.0** | Regeneration |
| 111. | Sambha -Sub1 | 14 | **100.0** | 9 | **64.3** | Good recovery |
| 112. | SANG KHLA | 12 | **85.7** | 5 | **35.7** | Escape |
| 113. | SANTI BAHN | 8 | **57.1** | 5 | **35.7** | Escape |
| 114. | SAVITRI | 7 | **50.0** | 0 | **0.0** |  |
| 115. | SINGHARA | 13 | **92.9** | 12 | **85.7** | Escape |
| 116. | SIPULUT PANDAN | 11 | **78.6** | 7 | **50.0** | Regeneration |
| 117. | SUGAPANKH | 11 | **78.6** | 0 | **0.0** | Escape |
| 118. | SWARNA | 0 | **0.0** | 0 | **0.0** | Susceptible |
| 119. | SWARNA-SUB1 | 13 | **92.9** | 0 | **0.0** | Regeneration |
| 120. | TAGBA | 13 | **92.9** | 4 | **28.6** | Elongated |
| 121. | TAH TONE | 10 | **71.4** | 0 | **0.0** | Escape |
| 122. | TATAN | 10 | **71.4** | 4 | **28.6** | Regeneration |
| 123. | TETEP | 14 | **100.0** | 3 | **21.4** | Regeneration |
| 124. | THUNMAR HAMAR | 9 | **64.3** | 3 | **21.4** | Regeneration |
| 125. | TIEBIMAH | 13 | **92.9** | 8 | **57.1** | Tips die |
| 126. | USA BATAPOLA AL | 14 | **100.0** | 0 | **0.0** | Escape |
| 127. | VADAI | 12 | **85.7** | 0 | **0.0** | Escape |
| 128. | VAN | 12 | **85.7** | 8 | **57.1** | Apex damaged |
| 129. | VANAN VELLAI | 14 | **100.0** | 3 | **21.4** | 40% tips die |
| 130. | WAHNG | 12 | **85.7** | 5 | **35.7** | Escape |
| 132. | ZOBGUI | 11 | **78.6** | 7 | **50.0** | Tiller mortality |

**Survival with canopy:**

**Population mean: 69.4%**

**Population range: 0 to 100%**

**Survival without canopy:**

**Population mean: 31.3%**

**Population range: 0 to 92.9%**

**Table S12.** Recovery of 170 varieties of rice plants after 15 days of complete submergence at **NDUAT, Ayodhya,** India in 2013. Total 20 well-established plants one month after direct sowing were submerged naturally in open fileds.

| **Sr. no.** | **Variety** | **No. Survived Rep I** | **% Survived Rep I** | **No. Survived Rep 2** | **% Survied Rep 2** | **% Survived Mean** |
| --- | --- | --- | --- | --- | --- | --- |
|  | AI CHIH AI KWOH 2 | 17 | 85 | 12 | 60 | **72.5** |
|  | ARC 12172 | 6 | 30 | 12 | 60 | **45** |
|  | ARC 614 | 10 | 50 | 14 | 70 | **60** |
|  | ASAMIA | 8 | 40 | 7 | 35 | **37.5** |
|  | ASAMIRUPA | 4 | 20 | 1 | 5 | **12.5** |
|  | ASANA | 2 | 10 | 8 | 40 | **25** |
|  | ASHRA LAKI | 0 | 0 | 7 | 35 | **17.5** |
|  | ASHU BHAJNA | 0 | 0 | 3 | 15 | **7.5** |
|  | AUS 287 | 7 | 35 | 11 | 55 | **45** |
|  | AUSBORO | 7 | 35 | 10 | 50 | **42.5** |
|  | AZUCENA | 5 | 25 | 7 | 35 | **30** |
|  | BABAI LACHHA | 6 | 30 | 2 | 10 | **20** |
|  | BABARAKSHA KARO | 6 | 30 | 1 | 5 | **17.5** |
|  | BAD DHANA | 0 | 0 | 2 | 10 | **5** |
|  | BAD GEDE | 0 | 0 | 1 | 5 | **2.5** |
|  | BAD JHARLI | 13 | 65 | 10 | 50 | **57.5** |
|  | BAD KODI | 0 | 0 | 0 | 0 | **0** |
|  | BAGA GOHA | 8 | 40 | 7 | 35 | **37.5** |
|  | BAGA IKRA | 2 | 10 | 7 | 35 | **22.5** |
|  | BAGI BARDHANA | 12 | 60 | 15 | 75 | **67.5** |
|  | BARA RANGA | 0 | 0 | 6 | 30 | **15** |
|  | BARA SALI | 8 | 40 | 6 | 30 | **35** |
|  | BARACHIRAMARA | 2 | 10 | 10 | 50 | **30** |
|  | BINULAWAN | 0 | 0 | 0 | 0 | **0** |
|  | BIRPALA | 3 | 15 | 14 | 70 | **42.5** |
|  | BR11 | 5 | 25 | 8 | 40 | **32.5** |
|  | BUNTOK | 8 | 40 | 7 | 35 | **37.5** |
|  | CHAKIA 59 | 6 | 30 | 13 | 65 | **47.5** |
|  | CHANG MUI | 3 | 15 | 6 | 30 | **22.5** |
|  | CHANGMAKA | 12 | 60 | 14 | 70 | **65** |
|  | CHANGMAN | 5 | 25 | 2 | 10 | **17.5** |
|  | CHANGNGAT | 0 | 0 | 0 | 0 | **0** |
|  | CHANGPHAI | 2 | 10 | 1 | 5 | **7.5** |
|  | CHASING | 0 | 0 | 0 | 0 | **0** |
|  | CHING CHANG | 1 | 5 | 4 | 20 | **12.5** |
|  | CHOW HA LA | 14 | 70 | 6 | 30 | **50** |
|  | CHOWMAI | 2 | 10 | 4 | 20 | **15** |
|  | CHUDI | 15 | 75 | 5 | 25 | **50** |
|  | CHUKKANNA KUMBALOAN | 9 | 45 | 10 | 50 | **47.5** |
|  | CHUMANSAL-39 | 2 | 10 | 7 | 35 | **22.5** |
|  | CR 1009 | 11 | 55 | 10 | 50 | **52.5** |
|  | DHOLAMON 64-3 | 13 | 65 | 12 | 60 | **62.5** |
|  | DHUSARI AHU | 1 | 5 | 6 | 30 | **17.5** |
|  | DIHAWAN | 9 | 45 | 3 | 15 | **30** |
|  | DUB GELONG | 0 | 0 | 0 | 0 | **0** |
|  | DUDHA LADU | 15 | 75 | 12 | 60 | **67.5** |
|  | DULAR | 12 | 60 | 12 | 60 | **60** |
|  | EMPING ARA | 0 | 0 | 0 | 0 | **0** |
|  | FR 13A | 11 | 55 | 15 | 75 | **65** |
|  | FR 43B | 17 | 85 | 13 | 65 | **75** |
|  | FRG1 | 11 | 55 | 14 | 70 | **62.5** |
|  | GODA HEENATI | 11 | 55 | 12 | 60 | **57.5** |
|  | GOTA | 0 | 0 | 0 | 0 | **0** |
|  | GOTHAWA | 0 | 0 | 0 | 0 | **0** |
|  | HEEN SULAI | 9 | 45 | 11 | 55 | **50** |
|  | HOURA KANI | 6 | 30 | 14 | 70 | **50** |
|  | HSUNG TIENG | 11 | 55 | 6 | 30 | **42.5** |
|  | IARI 5829 | 2 | 10 | 1 | 5 | **7.5** |
|  | IC 370811 | 0 | 0 | 0 | 0 | **0** |
|  | IR 36 | 12 | 60 | 8 | 40 | **50** |
|  | IR 42 | 5 | 25 | 8 | 40 | **32.5** |
|  | IR 64 | 11 | 55 | 16 | 80 | **67.5** |
|  | IR 72 | 9 | 45 | 8 | 40 | **42.5** |
|  | IR-73707-45-3-23 | 11 | 55 | 9 | 45 | **50** |
|  | JADAHAN | 6 | 30 | 1 | 5 | **17.5** |
|  | JAGANATHA BALLAVA | 1 | 5 | 6 | 30 | **17.5** |
|  | JAHABHOG | 4 | 20 | 7 | 35 | **27.5** |
|  | JALDUNGI | 0 | 0 | 0 | 0 | **0** |
|  | JALKANTHI | 1 | 5 | 5 | 25 | **15** |
|  | JALMAGNA | 9 | 45 | 10 | 50 | **47.5** |
|  | JALNIDHI | 1 | 5 | 9 | 45 | **25** |
|  | JAYMIS | 13 | 65 | 13 | 65 | **65** |
|  | JOHAINEANG (WET ) | 13 | 65 | 6 | 30 | **47.5** |
|  | JOKHRU | 3 | 15 | 1 | 5 | **10** |
|  | KAJARAHAWA | 4 | 20 | 3 | 15 | **17.5** |
|  | KALA BUNDE | 0 | 0 | 6 | 30 | **15** |
|  | KALONCHI | 12 | 60 | 14 | 70 | **65** |
|  | KALUWEE | 12 | 60 | 13 | 65 | **62.5** |
|  | KAOLACK | 5 | 25 | 11 | 55 | **40** |
|  | KARABONKA | 4 | 20 | 2 | 10 | **15** |
|  | KARIYAWA | 4 | 20 | 13 | 65 | **42.5** |
|  | KARKATI 87 | 12 | 60 | 13 | 65 | **62.5** |
|  | KARNAL LOCAL | 0 | 0 | 5 | 25 | **12.5** |
|  | KASALATH | 12 | 60 | 7 | 35 | **47.5** |
|  | KATY | 8 | 40 | 8 | 40 | **40** |
|  | KHAIYAN | 3 | 15 | 9 | 45 | **30** |
|  | KHAO HLAN ON | 2 | 10 | 7 | 35 | **22.5** |
|  | KHAO MINE LAR | 8 | 40 | 4 | 20 | **30** |
|  | KHARSU 80A: *Oryza spp* | 5 | 25 | 8 | 40 | **32.5** |
|  | KHAWJII | 9 | 45 | 4 | 20 | **32.5** |
|  | KOLA JOHA | 1 | 5 | 4 | 20 | **12.5** |
|  | KOTTAMALI | 14 | 70 | 11 | 55 | **62.5** |
|  | KRETEK SIRENDAH MERAH | 5 | 25 | 4 | 20 | **22.5** |
|  | KURUWEE | 1 | 5 | 6 | 30 | **17.5** |
|  | LABELLE | 10 | 50 | 9 | 45 | **47.5** |
|  | LATSIKA | 2 | 10 | 8 | 40 | **25** |
|  | LIU-TIAO-NU | 1 | 5 | 1 | 5 | **5** |
|  | LUNISHREE | 8 | 40 | 7 | 35 | **37.5** |
|  | MA-ZHAN(RED) | 3 | 15 | 6 | 30 | **22.5** |
|  | MADAL | 7 | 35 | 12 | 60 | **47.5** |
|  | MAHA DIKWEE | 13 | 65 | 12 | 60 | **62.5** |
|  | MAINTIMOLOTSY | 0 | 0 | 8 | 40 | **20** |
|  | MANDIRA | 1 | 5 | 4 | 20 | **12.5** |
|  | MANOHAR SALI | 0 | 0 | 10 | 50 | **25** |
|  | MARS | 5 | 25 | 3 | 15 | **20** |
|  | MEGHA BADALA | 4 | 20 | 1 | 5 | **12.5** |
|  | MEGHARAJ | 6 | 30 | 10 | 50 | **40** |
|  | MEGHI | 1 | 5 | 7 | 35 | **20** |
|  | MENYARHUNEI | 10 | 50 | 7 | 35 | **42.5** |
|  | MOROBEREKAN | 16 | 80 | 12 | 60 | **70** |
|  | MOTI | 12 | 60 | 1 | 5 | **32.5** |
|  | MOUSSAYA | 9 | 45 | 10 | 50 | **47.5** |
|  | NAALUMOLI KARUPPAN | 12 | 60 | 10 | 50 | **55** |
|  | NALDAK | 7 | 35 | 11 | 55 | **45** |
|  | NCS 348 | 11 | 55 | 10 | 50 | **52.5** |
|  | NIRBOI | 10 | 50 | 11 | 55 | **52.5** |
|  | PAIAIM | 13 | 65 | 1 | 5 | **35** |
|  | PAKRIBUNA | 6 | 30 | 5 | 25 | **27.5** |
|  | PALENEMA | 3 | 15 | 6 | 30 | **22.5** |
|  | PALIANDEH | 5 | 25 | 9 | 45 | **35** |
|  | PANI DHAN 2 | 1 | 5 | 7 | 35 | **20** |
|  | PANIDUBI | 12 | 60 | 6 | 30 | **45** |
|  | PANIKAKUA | 7 | 35 | 7 | 35 | **35** |
|  | PANIKEKEUA | 0 | 0 | 3 | 15 | **7.5** |
|  | PANKEI | 0 | 0 | 2 | 10 | **5** |
|  | PANNEL | 4 | 20 | 10 | 50 | **35** |
|  | PATAIM | 12 | 60 | 11 | 55 | **57.5** |
|  | PAYEMA | 7 | 35 | 12 | 60 | **47.5** |
|  | PECALO | 10 | 50 | 9 | 45 | **47.5** |
|  | POKKALI | 14 | 70 | 9 | 45 | **57.5** |
|  | RAJBHOG | 4 | 20 | 2 | 10 | **15** |
|  | RENIK BATU | 15 | 75 | 8 | 40 | **57.5** |
|  | ROM RUNTIK | 5 | 25 | 3 | 15 | **20** |
|  | S-138 | 10 | 50 | 10 | 50 | **50** |
|  | S-141 | 5 | 25 | 1 | 5 | **15** |
|  | S-148 | 0 | 0 | 12 | 60 | **30** |
|  | S-155 | 8 | 40 | 7 | 35 | **37.5** |
|  | S-177 | 11 | 55 | 8 | 40 | **47.5** |
|  | S-183 | 2 | 10 | 7 | 35 | **22.5** |
|  | SAMBHA-SUB1 | 3 | 15 | 13 | 65 | **40** |
|  | SANG KHLA | 4 | 20 | 10 | 50 | **35** |
|  | SANTI BAHN | 0 | 0 | 5 | 25 | **12.5** |
|  | SATHWA | 6 | 30 | 5 | 25 | **27.5** |
|  | SAVITRI | 0 | 0 | 6 | 30 | **15** |
|  | SINGHARA | 6 | 30 | 10 | 50 | **40** |
|  | SIPULUT PANDAN | 1 | 5 | 3 | 15 | **10** |
|  | SITA MATA | 7 | 35 | 13 | 65 | **50** |
|  | SOSSOKA | 7 | 35 | 11 | 55 | **45** |
|  | SUGAPANKH | 16 | 80 | 5 | 25 | **52.5** |
|  | SURAHA | 2 | 10 | 6 | 30 | **20** |
|  | SWARNA | 5 | 25 | 8 | 40 | **32.5** |
|  | SWARNA-SUB1 | 13 | 65 | 7 | 35 | **50** |
|  | TAGBA | 5 | 25 | 7 | 35 | **30** |
|  | TAH TONE | 0 | 0 | 4 | 20 | **10** |
|  | TATAN | 12 | 60 | 11 | 55 | **57.5** |
|  | TETEP | 8 | 40 | 13 | 65 | **52.5** |
|  | THUNMAR HAMARA | 4 | 20 | 10 | 50 | **35** |
|  | TIEBIMAH | 3 | 15 | 5 | 25 | **20** |
|  | USA BATAPOLA AL | 2 | 10 | 3 | 15 | **12.5** |
|  | VADAI | 10 | 50 | 10 | 50 | **50** |
|  | VAN | 0 | 0 | 7 | 35 | **17.5** |
|  | VANAN VELLAI | 11 | 55 | 14 | 70 | **62.5** |
|  | WAHNG | 5 | 25 | 2 | 10 | **17.5** |
|  | YEIKOYON | 6 | 30 | 8 | 40 | **35** |

**Population mean: 33.4%**

**Population range: 0 to 75%**

**Table S13.** Percent survival of rice plants of 126 cultivars after 14 days of complete submergence in a pond at **BHU, Varanasi,** India in 2012. Variable numbers of up to 20 well-established plants after 21 days of seedling growth were submerged with flood water.

| **Sr. no.** | **Variety** | **No. of**  **plants treated** | **No. of plants survived** | **%**  **Survival** |
| --- | --- | --- | --- | --- |
|  | ARC 614 | 20 | 0 | **0** |
|  | ASAMIA | 18 | 3 | **16.6** |
|  | ASANA | 20 | 0 | **0** |
|  | ASHU BHAJNA | 20 | 0 | **0** |
|  | AUSBORO | 20 | 16 | **80** |
|  | AZUCENA | 18 | 0 | **0** |
|  | BABAI LACHHA | 20 | 0 | **0** |
|  | BABARAKSHA KARO | 20 | 0 | **0** |
|  | BAD DHANA | 20 | 0 | **0** |
|  | BAD GEDE | 20 | 0 | **0** |
|  | BAD JHARLI | 18 | 0 | **0** |
|  | BAGA GOHA | 17 | 1 | **5.8** |
|  | BAGI BARDHANA | 20 | 0 | **0** |
|  | BARA RANGA | 20 | 1 | **5** |
|  | BARA SALI | 20 | 1 | **5** |
|  | BARACHIRAMARA | 12 | 0 | **0** |
|  | BINULAWAN | 18 | 0 | **0** |
|  | BIRPALA | 20 | 13 | **65** |
|  | BR11 | 20 | 1 | **5** |
|  | BUNTOK | 20 | 0 | **0** |
|  | CHANG MUI | 20 | 0 | **0** |
|  | CHANGMAKA | 20 | 1 | **5** |
|  | CHANGMAN | 19 | 0 | **0** |
|  | CHOW HA LA | 20 | 1 | **5** |
|  | CHUDI | 20 | 1 | **5** |
|  | CHUKKANNA KUMBALOAN | 20 | 0 | **0** |
|  | CHULTA BALUNGA (WILD) | 20 | 0 | **0** |
|  | CHUMANSAL-39 | 20 | 1 | **5** |
|  | CR 1009 | 20 | 6 | **30** |
|  | DHOLAMON 64-3 | 11 | 0 | **0** |
|  | DUDHA LADU | 20 | 0 | **0** |
|  | DULAR | 10 | 6 | **60** |
|  | EMPING ARA | 20 | 0 | **0** |
|  | FR 13A | 20 | 20 | **100** |
|  | FR 43B | 20 | 20 | **100** |
|  | GODA HEENATI | 20 | 13 | **65** |
|  | GOTHAWA | 19 | 0 | **0** |
|  | HEEN SULAI | 20 | 8 | **40** |
|  | HOURA KANI | 20 | 15 | **75** |
|  | HSUNG TIENG | 20 | 0 | **0** |
|  | IC370811 | 20 | 0 | **0** |
|  | IR 36 | 20 | 0 | **0** |
|  | IR 42 | 20 | 0 | **0** |
|  | IR 64 | 20 | 3 | **15** |
|  | IR-73707-45-3-23 | 20 | 0 | **0** |
|  | JAGANATHA BALLAVA | 19 | 1 | **5.2** |
|  | JAHABHOG | 13 | 0 | **0** |
|  | JALDUNGI | 20 | 6 | **30** |
|  | JALKANTHI | 18 | 1 | **5.5** |
|  | JALNIDHI | 19 | 0 | **0** |
|  | JAYMIS | 16 | 0 | **0** |
|  | JC1 | 19 | 0 | **0** |
|  | JOHAINEANG(WET ) | 20 | 0 | **0** |
|  | JOKHRU | 20 | 0 | **0** |
|  | KAJARAHAWA | 20 | 0 | **0** |
|  | KALA BUNDE | 20 | 0 | **0** |
|  | KALUWEE | 19 | 1 | **5.26** |
|  | KAOLACK | 19 | 0 | **0** |
|  | KARABONKA | 19 | 0 | **0** |
|  | KARIYAWA | 20 | 7 | **35** |
|  | KARNAL LOCAL | 20 | 0 | **0** |
|  | KASALATH | 20 | 3 | **15** |
|  | KHAIYAN | 20 | 0 | **0** |
|  | KHAO HLAN ON | 19 | 1 | **5.26** |
|  | KOLA JOHA | 20 | 0 | **0** |
|  | KRETEK SIRENDAH MERAH | 20 | 1 | **5** |
|  | LATSIKA | 20 | 0 | **0** |
|  | LEMO | 20 | 0 | **0** |
|  | LIU-TIAO-NU | 20 | 0 | **0** |
|  | LUNISHREE | 20 | 4 | **20** |
|  | MA-ZHAN(RED) | 20 | 0 | **0** |
|  | MADAL | 20 | 7 | **35** |
|  | MAHA DIKWEE | 20 | 17 | **85** |
|  | MAINTIMOLOTSY | 18 | 8 | **44.44** |
|  | MANDIRA | 20 | 0 | **0** |
|  | MANOHAR SALI | 20 | 1 | **5** |
|  | MARS | 20 | 2 | **10** |
|  | MEGHARAJ | 20 | 0 | **0** |
|  | MEGHI | 20 | 1 | **5** |
|  | MENYARHUNEI | 20 | 0 | **0** |
|  | MOTI | 20 | 1 | **5** |
|  | MOUSSAYA | 20 | 1 | **5** |
|  | NAALUMOLI KARUPPAN | 20 | 0 | **0** |
|  | NALDAK | 20 | 15 | **75** |
|  | NCS 348 | 20 | 0 | **0** |
|  | NIRBOI | 20 | 8 | **40** |
|  | PAIAIM | 20 | 0 | **0** |
|  | PAKRIBUNA | 20 | 3 | **15** |
|  | PALENEMA | 20 | 0 | **0** |
|  | PANI DHAN 2 | 19 | 1 | **5.26** |
|  | PANIDUBI | 20 | 2 | **10** |
|  | PANIKAKUA | 20 | 0 | **0** |
|  | PANIKEKEUA | 20 | 1 | **5** |
|  | PANKEI | 16 | 2 | **12.5** |
|  | PANNEL | 20 | 1 | **5** |
|  | PATAIM | 20 | 1 | **5** |
|  | PECALO | 20 | 0 | **0** |
|  | RAJBHOG | 19 | 1 | **5.26** |
|  | RENIK BATU | 20 | 0 | **0** |
|  | ROM RUNTIK | 20 | 0 | **0** |
|  | S-138 | 20 | 2 | **10** |
|  | S-141 | 19 | 1 | **5.26** |
|  | S-148 | 20 | 1 | **5** |
|  | S-155 | 20 | 4 | **20** |
| 1. - | S-177 | 19 | 1 | **5.26** |
|  | S-183 | 20 | 0 | **0** |
|  | SAMBHA -SUB1 | 20 | 19 | **95** |
|  | SANG KHLA | 20 | 1 | **5** |
|  | SANTI BAHN | 20 | 1 | **5** |
|  | SAVITRI | 20 | 0 | **0** |
|  | SINGHARA | 19 | 3 | **15.7** |
|  | SIPULUT PANDAN | 20 | 1 | **5** |
|  | SUGAPANKH | 20 | 1 | **5** |
|  | SWARNA | 20 | 0 | **0** |
|  | SWARNA-SUB1 | 20 | 5 | **25** |
|  | TAGBA | 19 | 0 | **0** |
|  | TAH TONE | 20 | 1 | **5** |
|  | TATAN | 20 | 0 | **0** |
|  | TETEP | 20 | 8 | **40** |
|  | TIEBIMAH | 20 | 0 | **0** |
|  | USA BATAPOLA AL | 20 | 0 | **0** |
|  | VADAI | 20 | 4 | **20** |
|  | VAN | 20 | 0 | **0** |
|  | VANAN VELLAI | 10 | 0 | **0** |
|  | WAHNG | 20 | 1 | **5** |
|  | ZOBGUI | 20 | 0 | **0** |

**Population mean: 11.6%**

**Population range: 0 to 100%**

**Table S14.**  Survival data on 175 rice cultivars after complete submergence in muddy water for 12 days in a field submergence pond at RPCAU, Samastipur, India in 2012

| **S. No.** | **Variety** | **No. of**  **seeds sown** | **No. of seed germinated** | **No. of plants survived** | **Survival**  **(%)** |
| --- | --- | --- | --- | --- | --- |
|  | AI CHIH AI KWOH 2 | 50 | 25 | 10 | **40** |
|  | AKITAKOMAHCI | 50 | 8 | 8 | **100** |
|  | ARC 12172 | 50 | 28 | 20 | **72.73** |
|  | ARC 614 | 50 | 25 | 25 | **100** |
|  | ASAMIRUPA | 50 | 13 | 13 | **100** |
|  | ASANA | 50 | 25 | 25 | **100** |
|  | ASHRA LAKI | 50 | 15 | 8 | **50** |
|  | ASHUBHAJNA | 50 | 35 | 25 | **71.43** |
|  | ASMIA | 50 | 30 | 18 | **58.33** |
|  | AUS 287 | 50 | 28 | 28 | **100** |
|  | AUSBORO | 50 | 35 | 25 | **71.43** |
|  | AZUCENA | 50 | 28 | 28 | **100** |
|  | BABAI LACHHA | 50 | 28 | 18 | **63.64** |
|  | BABARAKSHA | 50 | 28 | 25 | **90.91** |
|  | BAD JHARLI | 50 | 40 | 30 | **75** |
|  | BAD KODI | 50 | 18 | 18 | **100** |
|  | BADDHANA | 50 | 20 | 18 | **87.5** |
|  | BADGEDE | 50 | 30 | 23 | **75** |
|  | BAGA IKRA | 50 | 15 | 10 | **66.67** |
|  | BAGAGOHA | 50 | 25 | 20 | **80** |
|  | BAGI BARDHANA | 50 | 38 | 25 | **66.67** |
|  | BARA SALI | 50 | 33 | 20 | **61.54** |
|  | BARACHIRAMARA | 50 | 23 | 18 | **77.78** |
|  | BARARANGA | 50 | 30 | 28 | **91.67** |
|  | BINULAWAN | 50 | 20 | 15 | **75** |
|  | BIRPALA | 50 | 23 | 18 | **77.78** |
|  | BR 11 | 50 | 20 | 20 | **100** |
|  | BUNTOK | 50 | 18 | 18 | **100** |
|  | CHAKIA 59 | 50 | 23 | 23 | **100** |
|  | CHANGMAKA | 50 | 33 | 30 | **92.31** |
|  | CHANGMAN | 50 | 25 | 25 | **100** |
|  | CHANGMUI | 50 | 25 | 20 | **80** |
|  | CHANGNGAT | 50 | 18 | 18 | **100** |
|  | CHANGPHAI | 50 | 35 | 25 | **71.43** |
|  | CHASING | 50 | 25 | 25 | **100** |
|  | CHING CHANG | 50 | 23 | 23 | **100** |
|  | CHOWHALA | 50 | 23 | 18 | **77.78** |
|  | CHOWMAI | 50 | 48 | 35 | **73.68** |
|  | CHUDI | 50 | 23 | 23 | **100** |
|  | CHUKKANNA KUMBALOAN | **50** | 30 | 15 | **50** |
|  | CHULTA BALUNGA | 50 | 33 | 30 | **92.31** |
|  | CHUMANSAL-39 | 50 | 30 | 18 | **58.33** |
|  | CR1009 | 50 | 18 | 13 | **71.43** |
|  | DHOLAMON 64-3 | 50 | 28 | 23 | **81.82** |
|  | DHUSARI AHU | 50 | 23 | 23 | **100** |
|  | DIHAWAN | 50 | 28 | 25 | **90.91** |
|  | DUB GELONG | 50 | 35 | 23 | **64.29** |
|  | DUDHA LADU | 50 | 25 | 25 | **100** |
|  | DULAR | 50 | 33 | 28 | **84.62** |
|  | EMPING ARA | 50 | 33 | 23 | **69.23** |
|  | FR13A | 50 | 38 | 35 | **92.10** |
|  | FR43B | 50 | 33 | 33 | **100** |
|  | FRG1 | 50 | 13 | 13 | **100** |
|  | GODA HEENATI | 50 | 18 | 10 | **57.14** |
|  | GOTA | 50 | 25 | 25 | **100** |
|  | GOTHAWA | 50 | 28 | 20 | **72.73** |
|  | HEEN SULAI | 50 | 10 | 8 | **75** |
|  | HOURA | 50 | 30 | 23 | **75** |
|  | HSUNG TIENG | 50 | 38 | 30 | **80** |
|  | IC 370811 | 50 | 20 | 20 | **100** |
|  | IARI 5829 | 50 | 25 | 20 | **80** |
|  | IR-36 | 50 | 23 | 20 | **88.89** |
|  | IR-73707-45-3-23 | 50 | 30 | 20 | **66.67** |
|  | IR42 | 50 | 25 | 15 | **60** |
|  | IR64 | 50 | 25 | 23 | **90** |
|  | IR72 | 50 | 28 | 20 | **72.73** |
|  | JADHAN | 50 | 28 | 28 | **100** |
|  | JAGANATHA BALLAVA | 50 | 23 | 23 | **100** |
|  | JAHABHOG | 50 | 15 | 13 | **83.33** |
|  | JALDUNGI | 50 | 30 | 25 | **83.33** |
|  | JALKANTHI | 50 | 25 | 20 | **80** |
|  | JALMAGNA | 50 | 35 | 33 | **92.86** |
|  | JALNIDHI | 50 | 25 | 20 | **80** |
|  | JAYMIS | 50 | 10 | 5 | **50** |
|  | JCI | 50 | 23 | 20 | **88.89** |
|  | JOHAINEANG (WET) | 50 | 18 | 15 | **85.71** |
|  | JOKHRU | 50 | 23 | 15 | **66.67** |
|  | KAJARAHAWA | 50 | 33 | 25 | **76.92** |
|  | KALONCHI | 50 | 23 | 18 | **77.78** |
|  | KALUWEE | 50 | 13 | 10 | **80** |
|  | KAMNAM | 50 | 3 | 3 | **100** |
|  | KAOLACK | 50 | 35 | 28 | **78.57** |
|  | KARABONKA | 50 | 25 | 25 | **100** |
|  | KARIYWA | 50 | 30 | 25 | **83.33** |
|  | KARKATI 87 | 50 | 45 | 40 | **88.89** |
|  | KARNAL LOCAL | 50 | 18 | 15 | **85.71** |
|  | KASALATH | 50 | 20 | 13 | **62.5** |
|  | KATY | 50 | 20 | 13 | **62.5** |
|  | KHAIYAN | 50 | 23 | 20 | **88.89** |
|  | KHAO HLAO ON | 50 | 15 | 10 | **66.67** |
|  | KHAO MINE LAR | 50 | 30 | 25 | **83.33** |
|  | KHARSU 80A | 50 | 28 | 18 | **63.64** |
|  | KHAWJII | 50 | 10 | 10 | **100** |
|  | KOTTAMALI | 50 | 15 | 15 | **100** |
|  | KRETEK SIRENDAH MERAH | 50 | 15 | 15 | **100** |
|  | KURUWEE | 50 | 28 | 23 | **81.82** |
|  | LABELLE | 50 | 23 | 20 | **88.89** |
|  | LATSIKA | 50 | 35 | 33 | **92.86** |
|  | LEMO | 50 | 13 | 10 | **80** |
|  | LEMONT | 50 | 23 | 20 | **88.89** |
|  | LIU-TIAO-NU | 50 | 23 | 23 | **100** |
|  | LUNISHREE | 50 | 23 | 18 | **77.78** |
|  | M202 | 50 | 28 | 20 | **72.73** |
|  | MA-ZHAN(RED) | 50 | 30 | 30 | **100** |
|  | MADAL | 50 | 23 | 13 | **55.56** |
|  | MAHADIKWEE | 50 | 15 | 15 | **100** |
|  | MAINTI MOLOTSY | 50 | 35 | 15 | **42.86** |
|  | MANDIRA | 50 | 35 | 28 | **78.57** |
|  | MANOHAR SALI | 50 | 25 | 18 | **70** |
|  | MARS | 50 | 15 | 15 | **100** |
|  | MEGHA BADALA | 50 | 35 | 23 | **64.29** |
|  | MEGHARAJ | 50 | 23 | 20 | **88.89** |
|  | MEGHI | 50 | 35 | 25 | **71.43** |
|  | MENYARHUNEI | 50 | 38 | 33 | **86.67** |
|  | MOROBEREKAN | 50 | 18 | 18 | **100** |
|  | MOTI | 50 | 18 | 15 | **85.71** |
|  | MOUSSAYA | 50 | 28 | 25 | **90.91** |
|  | NAALUMOLI | 50 | 25 | 23 | **90** |
|  | NALDAK | 50 | 13 | 13 | **100** |
|  | NCS 348 | 50 | 25 | 23 | **90** |
|  | NIRBOI | 50 | 25 | 23 | **90** |
|  | PADMA | 50 | 3 | 3 | **100** |
|  | PAIAIM | 50 | 25 | 25 | **100** |
|  | PAKRIBUNA | 50 | 23 | 20 | **88.89** |
|  | PALENEMA | 50 | 20 | 20 | **100** |
|  | PALIANDEH | 50 | 23 | 18 | **77.78** |
|  | PANIDHAN 2 | 50 | 30 | 18 | **58.33** |
|  | PANIDUBI | 50 | 38 | 30 | **80** |
|  | PANIKAKUA | 50 | 23 | 23 | **100** |
|  | PANIKEKEUA | 50 | 10 | 10 | **100** |
|  | PANKEI | 50 | 33 | 33 | **100** |
|  | PANNEL | 50 | 28 | 25 | **90.91** |
|  | PANSANKO | 50 | 13 | 13 | **100** |
|  | PATAIM | 50 | 13 | 8 | **60** |
|  | PAYEMA | 50 | 25 | 23 | **90** |
|  | PECALO | 50 | 23 | 18 | **77.78** |
|  | POKKALI | 50 | 10 | 8 | **75** |
|  | RAJBHOG | 50 | 18 | 18 | **100** |
|  | RENIK BATU | 50 | 23 | 23 | **100** |
|  | ROM RUNTIK | 50 | 13 | 13 | **100** |
|  | S-138 | 50 | 28 | 25 | **90.91** |
|  | S-141 | 50 | 28 | 28 | **100** |
|  | S-148 | 50 | 30 | 28 | **91.67** |
|  | S-150 | 50 | 23 | 23 | **100** |
|  | S-155 | 50 | 25 | 25 | **100** |
|  | S-172 | 50 | 33 | 28 | **84.62** |
|  | S-177 | 50 | 23 | 18 | **77.78** |
|  | S-183 | 50 | 30 | 18 | **58.33** |
|  | SAMBHA | 50 | 18 | 18 | **100** |
|  | SANG KHLA | 50 | 33 | 30 | **92.31** |
|  | SANTI BAHN | 50 | 40 | 33 | **81.25** |
|  | SATHWA | 50 | 15 | 10 | **66.67** |
|  | SAVITRI | 50 | 28 | 25 | **90.91** |
|  | SINGHARA | 50 | 30 | 28 | **91.67** |
|  | SIPULUT PANDAN | 50 | 15 | 10 | **66.67** |
|  | SITA MATA | 50 | 30 | 25 | **83.33** |
|  | SOSSOKA | 50 | 25 | 23 | **90** |
|  | SUGAPANKH | 50 | 33 | 23 | **69.23** |
|  | SURAHA | 50 | 3 | 3 | **100** |
|  | SWARNA | 50 | 15 | 15 | **100** |
|  | SWARNA SUB 1 | 50 | 15 | 13 | **83.33** |
|  | TAGBA | 50 | 30 | 28 | **91.67** |
|  | TAH TONE | 50 | 43 | 33 | **76.47** |
|  | TATAN | 50 | 25 | 25 | **100** |
|  | TETEP | 50 | 3 | 3 | **100** |
|  | THUNMAR HUMARA | 50 | 20 | 13 | **62.5** |
|  | TIEBIMAH | 50 | 25 | 25 | **100** |
|  | TUNDAHIYA | 50 | 13 | 13 | **100** |
|  | USA BATAPOLA AL | 50 | 18 | 10 | **57.14** |
|  | VADAI | 50 | 20 | 20 | **100** |
|  | VAN | 50 | 18 | 18 | **100** |
|  | VANAN VELLAI | 50 | 15 | 15 | **100** |
|  | WAHNG | 50 | 45 | 40 | **88.89** |
|  | YEIKOYON | 50 | 8 | 5 | **66.67** |
|  | ZOBGUI | 50 | 25 | 25 | **100** |

**Population mean: 84.6%**

**Population range: 42.9 to 100%**

**Supplementary Table S15.** Comparison of the best and the worse performing cultivars in the six submergence tolerance trials at four locations during 2012 and 2013

**(Provided as separate Excel file)**

**Table S16.** Data on population STRUCTURE (at K= 4), *Sub1A1* allele-specific AEX marker amplification, re-sequencing based SNP haplotypes of the *Sub1* genes, and percent survival of plants after 14 days of complete submergence of 179 rice cultivars at IRRI, Philippines in 2012, used in the development of 3D plots for visualization of their association.

| **Sr. No.** | **Variety** | **Sub-population (K-4)** | **AEX1 Amplification** | **Sub1A Haplotype** | **Sub1B Haplotype** | **Sub1C Haplotype** | **Percent survival Rep 1** | **Percent survival Rep 2l** | **Percent survival Average** |
| --- | --- | --- | --- | --- | --- | --- | --- | --- | --- |
|  | AI CHIH AI KWOH | 2 |  |  |  | H67 | 44.4 | 42.1 | **43.3** |
|  | AKITAKOMACHI | 4 |  |  |  | H6 |  |  |  |
|  | ARC 12172 | 3 |  | H8 | H8 | H32 | 100.0 | 72.2 | **86.1** |
|  | ARC 614 | 1 |  | H2 | H6 | H31 | 89.5 | 100.0 | **94.7** |
|  | ASAMIA | 2 |  | H8 | H9 | H1 | 95.2 | 84.2 | **89.7** |
|  | ASAMIRUPA | 1 |  |  |  | H39 | 70.0 | 30.0 | **50.0** |
|  | ASANA | 2 |  | H2 | H6 | H47 | 77.3 | 52.4 | **64.8** |
|  | ASHRA LAKI | 1 |  | H8 | H9 | H1 | 91.7 | 42.1 | **66.9** |
|  | ASHU BHAJNA | 2 |  | H8 | H5 | H60 | 47.6 | 77.8 | **62.7** |
|  | AUS 287 | 3 |  |  | H9 | H20 | 35.0 | 80.0 | **57.5** |
|  | AUSBORO | 3 |  | H8 | H9 | H1 | 46.7 | 75.0 | **60.8** |
|  | AZUCENA | 4 |  | H8 | H6 | H6 | 36.8 | 0.0 | **18.4** |
|  | BABAI LACHHA | 2 |  | H8 |  | H38 | 50.0 | 38.9 | **44.4** |
|  | BABARAKSHA KARO | 2 |  | H8 |  | H52 | 85.7 | 85.0 | **85.4** |
|  | BAD DHANA | 4 |  |  |  | H16 | 44.4 | 47.8 | **46.1** |
|  | BAD GEDE | 2 |  | H8 |  | H64 | 31.8 | 72.2 | **52.0** |
|  | BAD JHARLI | 1 |  | H2 | H6 | H42 | 73.7 | 61.1 | **67.4** |
|  | BAD KODI | 4 |  | H8 |  | H22 |  |  |  |
|  | BAGA GOHA | 4 |  |  |  | H6 | 66.7 | 0.0 | **33.3** |
|  | BAGA IKRA | 3 |  |  |  | H55 |  |  |  |
|  | BARA RANGA | 1 |  | H3 | H6 | H47 | 60.0 | 81.0 | **70.5** |
|  | BARA SALI | 1 |  | H2 | H6 | H61 | 100.0 | 65.0 | **82.5** |
|  | BARACHIRAMARA | 1 |  | H8 | H9 | H1 | 30.0 | 60.0 | **45.0** |
|  | BINULAWAN | 4 |  |  |  | H6 | 60.0 | 75.0 | **67.5** |
|  | BIRPALA | 1 |  | H3 | H6 | H26 | 40.0 | 84.2 | **62.1** |
|  | BR11 | 1 |  | H8 | H6 | H1 | 45.0 | 55.0 | **50.0** |
|  | BUNTOK | 1 |  | H8 |  | H36 | 65.0 | 75.0 | **70.0** |
|  | CHAKIA 59 | 2 |  | H8 | H9 | H1 | 86.4 | 38.1 | **62.2** |
|  | CHANG MUI | 1 |  | H3 | H6 | H39 | 70.0 | 65.0 | **67.5** |
|  | CHANGMAKA | 1 |  | H5 | H9 | H4 | 73.7 | 65.0 | **69.3** |
|  | CHANGMAN | 2 |  | H8 |  | H10 | 60.0 | 21.1 | **40.5** |
|  | CHANGNGAT | 2 |  |  |  | H37 |  |  |  |
|  | CHANGPHAI | 1 |  | H8 |  | H25 | 85.0 | 73.7 | **79.3** |
|  | CHASING | 4 |  |  |  | H54 | 0.0 | 57.9 | **28.9** |
|  | CHING CHANG | 2 |  | H8 |  | H23 |  |  |  |
|  | CHOW HA LA | 2 |  |  |  | H66 | 45.0 | 35.0 | **40.0** |
|  | CHOWMAI | 2 |  | H8 |  | H6 |  |  |  |
|  | CHUDI | 2 |  | H8 | H9 | H1 | 94.4 | 75.0 | **84.7** |
|  | CHUMANSAL 39 | 2 |  | H2 | H6 | H61 | 75.0 | 55.0 | **65.0** |
|  | CR 1009 | 2 |  | H2 | H6 | H17 | 65.2 | 80.0 | **72.6** |
|  | DHOLAMON 64-3 | 1 |  |  | H6 |  | 90.0 | 100.0 | **95.0** |
|  | DHUSARI AHU | 1 |  | H6 | H6 | H60 |  |  |  |
|  | DIHAWAN | 3 |  |  | H9 |  | 100.0 | 88.9 | **94.4** |
|  | DUB GELONG | 3 |  |  |  | H33 | 28.6 | 53.8 | **41.2** |
|  | DUDHA LADU | 1 |  | H2 | H6 | H61 | 40.0 | 90.0 | **65.0** |
|  | DULAR | 3 |  |  |  | H1 | 60.0 | 80.0 | **70.0** |
|  | EMPING ARA | 1 |  |  |  | H13 | 65.0 | 65.0 | **65.0** |
|  | FR 13 A | 3 |  | H8 | H9 | H1 | 95.2 | 90.9 | **93.1** |
|  | FR 43 B | 1 |  |  | H9 |  | 100.0 | 100.0 | **100.0** |
|  | FRG 1 | 2 |  |  | H6 |  | 95.7 | 80.0 | **87.8** |
|  | GODA HEENATI | 2 |  |  | H9 | H1 | 80.0 | 90.0 | **85.0** |
|  | GOTA | 2 |  |  | H6 | H44 |  |  |  |
|  | GOTHAWA | 2 |  | H2 | H6 | H61 | 94.1 | 55.0 | **74.6** |
|  | HEEN SULAI | 2 |  | H8 | H9 | H1 | 45.0 | 85.0 | **65.0** |
|  | HOURA KANI | 1 |  |  | H6 | H45 | 89.5 | 84.2 | **86.8** |
|  | HSUNG TIENG | 2 |  |  |  | H50 | 60.0 | 80.0 | **70.0** |
|  | CHUKKANNA KUMBALOA | 2 |  | H8 | H6 | H30 | 20.0 | 57.9 | **38.9** |
|  | CHULTA BALUNGA | 1 |  | H2 |  | H52 | 60.0 | 80.0 | **70.0** |
|  | IARI 5825 | 2 |  |  |  | H13 | 57.1 | 33.3 | **45.2** |
|  | IC370811 | 2 |  | H2 | H6 | H61 | 89.5 | 42.9 | **66.2** |
|  | IR 36 | 2 |  | H2 | H6 | H47 | 100.0 | 100.0 | **100.0** |
|  | IR 42 | 2 |  |  |  | H13 | 38.1 | 31.6 | **34.8** |
|  | IR 64 | 2 |  | H2 | H6 | H29 | 100.0 | 89.5 | **94.7** |
|  | IR 72 | 2 |  |  |  | H37 | 47.4 | 88.9 | **68.1** |
|  | IR 73707-45-3-23 | 2 |  |  | H6 | H57 |  |  |  |
|  | JADHAN | 2 |  | H3 | H6 | H47 | 45.0 | 17.6 | **31.3** |
|  | JAGANATHA BALLAVA | 1 |  | H2 | H6 | H59 | 25.0 | 80.0 | **52.5** |
|  | JAHABHOG | 4 |  | H3 | H6 | H45 | 94.7 | 95.0 | **94.9** |
|  | JALDUNGI | 1 |  | H2 | H6 | H31 | 47.6 | 35.0 | **41.3** |
|  | JALKANTHI | 2 |  |  |  | H50 | 50.0 | 65.0 | **57.5** |
|  | JALMAGNA | 4 |  | H8 | H2 | H14 | 50.0 | 8.3 | **29.2** |
|  | JALNIDHI | 1 |  |  |  | H50 | 70.0 | 38.1 | **54.0** |
|  | JAYMIS | 2 |  | H2 | H6 | H47 | 66.7 | 30.0 | **48.3** |
|  | JC1 | 4 |  |  |  | H5 | 35.0 | 57.1 | **46.1** |
|  | JOHAINEANG (WET) | 2 |  | H8 |  | H50 | 90.0 | 66.7 | **78.3** |
|  | JOKHRU | 4 |  |  |  | H37 | 75.0 | 31.6 | **53.3** |
|  | KAJARAHAWA | 3 |  | H2 | H9 | H43 | 93.8 | 105.6 | **99.7** |
|  | KALA BUNDE | 2 |  |  |  | H48 | 60.0 | 47.1 | **53.5** |
|  | KALONCHI | 3 |  | H8 | H9 | H1 | 68.4 | 63.2 | **65.8** |
|  | KALUWEE | 2 |  | H2 | H6 | H60 | 84.2 | 100.0 | **92.1** |
|  | KAMNAM | 4 |  |  |  | H58 | 94.7 | 90.0 | **92.4** |
|  | KAOLACK | 1 |  | H2 | H4 |  | 50.0 | 38.1 | **44.0** |
|  | KARABONKA | 1 |  |  |  | H52 | 75.0 | 90.0 | **82.5** |
|  | KARIYWA | 2 |  | H1 | H6 | H29 | 75.0 | 38.9 | **56.9** |
|  | KARKATI 87 | 2 |  | H8 | H6 | H52 | 66.7 | 68.4 | **67.5** |
|  | KARNAL LOCAL | 4 |  | H8 | H8 | H11 | 95.0 | 90.0 | **92.5** |
|  | KASALATH | 3 |  | H8 | H9 | H1 | 68.4 | 55.0 | **61.7** |
|  | KATY | 4 |  | H8 | H6 | H9 | 86.4 | 100.0 | **93.2** |
|  | KHAIYAN | 3 |  | H8 |  | H1 | 88.9 | 94.7 | **91.8** |
|  | KHAO HLAN ON | 2 |  | H8 | H6 | H28 | 25.0 | 35.0 | **30.0** |
|  | KHAO MINE LAR | 2 |  |  | H6 | H43 | 42.9 | 25.0 | **33.9** |
|  | KHARSU 80A | 3 |  |  |  | H34 | 57.9 | 68.4 | **63.2** |
|  | KHAWJII | 1 |  | H3 | H9 | H29 |  |  |  |
|  | KOLA JOHA | 4 |  | H8 | H3 | H21 | 83.3 | 73.7 | **78.5** |
|  | KOTTAMALI | 2 |  |  | H6 |  | 100.0 | 77.8 | **88.9** |
|  | KURUWEE | 2 |  |  |  | H40 | 33.3 | 36.8 | **35.1** |
|  | LABELLE | 4 |  |  | H6 | H35 | 78.9 | 73.7 | **76.3** |
|  | LATSIKA | 4 |  |  |  | H13 | 90.0 | 65.0 | **77.5** |
|  | LEMO | 1 |  | H8 |  | H37 | 47.4 | 63.2 | **55.3** |
|  | LEMONT | 4 |  |  |  | H13 | 95.8 | 68.8 | **82.3** |
|  | LIU-TIAO-NU | 2 |  |  |  | H13 | 20.0 | 0.0 | **10.0** |
|  | LUNISHREE | 2 |  |  | H9 | H53 | 76.0 | 38.9 | **57.4** |
|  | M202 | 4 |  |  |  | H6 | 58.8 | 68.4 | **63.6** |
|  | MA ZHAN (RED) | 2 |  |  |  | H7 | 29.2 | 52.4 | **40.8** |
|  | MADAL | 2 |  |  | H6 | H29 | 85.7 | 80.0 | **82.9** |
|  | MADHUKAR | 2 |  | H2 | H6 | H36 | 90.0 | 89.5 | **89.7** |
|  | MAHA DIKWEE | 2 |  | H5 | H6 | H2 | 95.0 | 100.0 | **97.5** |
|  | MAINTIMOLOTSY | 4 |  | H4 | H6 | H61 | 41.2 | 27.8 | **34.5** |
|  | MANDIRA | 1 |  | H8 | H6 | H41 | 35.0 | 61.9 | **48.5** |
|  | MANOHAR SALI | 1 |  | H8 | H9 | H28 | 45.0 | 94.7 | **69.9** |
|  | MARS | 4 |  |  |  | H37 | 47.4 | 30.0 | **38.7** |
|  | MEGHA BADALA | 2 |  | H7 | H6 | H42 | 45.0 | 85.0 | **65.0** |
|  | MEGHARAJ | 4 |  |  | H9 | H47 | 33.3 | 80.0 | **56.7** |
|  | MEGHI | 3 |  |  |  | H56 | 86.4 | 100.0 | **93.2** |
|  | MENYAR HUNEI | 1 |  | H8 | H6 | H15 | 88.2 | 20.0 | **54.1** |
|  | MOROBEREKAN | 4 |  | H2 | H1 | H61 | 10.0 | 9.5 | **9.8** |
|  | MOTI | 2 |  |  |  | H37 | 80.0 | 78.9 | **79.5** |
|  | MOUSSAYA | 1 |  |  |  | H1 | 90.0 | 45.0 | **67.5** |
|  | NAALUMOLO KARUPPAN | 2 |  |  |  | H3 | 50.0 | 84.2 | **67.1** |
|  | NALDAK | 2 |  | H8 | H9 | H1 | 50.0 | 65.0 | **57.5** |
|  | NCS 348 | 3 |  | H8 | H9 | H1 | 90.0 | 95.0 | **92.5** |
|  | NIRBOI | 1 |  |  | H6 | H61 | 55.6 | 75.0 | **65.3** |
|  | PADMA | 2 |  |  |  | H50 |  |  |  |
|  | PAIAIM | 2 |  |  |  | H37 | 81.0 | 75.0 | **78.0** |
|  | PAKRIBUNA | 1 |  | H8 | H8 | H24 | 50.0 | 70.0 | **60.0** |
|  | PALENEMA | 1 |  |  |  | H37 | 90.0 | 52.0 | **71.0** |
|  | PALIANDEH | 1 |  |  |  | H39 |  |  |  |
|  | PANI DHAN 2 | 2 |  | H2 | H6 | H47 | 52.4 | 80.0 | **66.2** |
|  | PANIDUBI | 1 |  | H8 | H9 | H56 | 65.0 | 81.3 | **73.1** |
|  | PANIKAKUA | 2 |  | H2 | H6 | H61 | 85.0 | 45.0 | **65.0** |
|  | PANIKEKEUA | 2 |  | H2 | H6 | H46 | 35.0 | 75.0 | **55.0** |
|  | PANKEI | 1 |  | H8 | H9 | H1 | 84.2 | 78.9 | **81.6** |
|  | PANNEL | 1 |  |  |  | H53 | 63.2 | 85.0 | **74.1** |
|  | PANSANKO | 1 |  |  |  | H65 | 66.7 | 42.1 | **54.4** |
|  | PATAIM | 1 |  | H5 | H6 | H41 | 100.0 | 90.5 | **95.2** |
|  | PAYEMA | 1 |  | H3 | H6 | H47 | 61.9 | 90.0 | **76.0** |
|  | PECALO | 1 |  |  |  | H50 | 15.8 | 30.0 | **22.9** |
|  | POKKALI | 2 |  |  |  | H52 | 70.8 | 17.6 | **44.2** |
|  | RAJBHOG | 2 |  |  | H6 | H42 | 83.3 | 100.0 | **91.7** |
|  | RENIK BATU | 1 |  |  |  | H37 | 18.8 | 33.3 | **26.0** |
|  | RETEK SIRENDAH MERA | 1 |  |  |  | H50 | 88.9 | 66.7 | **77.8** |
|  | ROM RUNTIK | 1 |  | H3 | H4 | H61 | 31.6 | 0.0 | **15.8** |
|  | S 138 | 2 |  | H2 | H6 | H61 | 84.2 | 90.0 | **87.1** |
|  | S 141 | 2 |  | H2 | H6 | H61 | 95.0 | 89.5 | **92.2** |
|  | S 148 | 2 |  |  |  | H37 | 100.0 | 71.4 | **85.7** |
|  | S 150 | 2 |  |  | H9 | H49 |  |  |  |
|  | S 155 | 2 |  |  | H9 | H51 | 90.5 | 95.0 | **92.7** |
|  | S 172 | 2 |  | H8 |  | H63 |  |  |  |
|  | S 177 | 2 |  | H2 | H6 | H61 | 80.0 | 70.0 | **75.0** |
|  | S 183 | 2 |  |  |  | H37 | 80.0 | 70.0 | **75.0** |
|  | SAMBHA-SUB1 | 2 |  | H8 | H9 | H1 | 100.0 | 66.7 | **83.3** |
|  | SANG KHLA | 1 |  |  |  | H37 | 68.2 | 94.7 | **81.5** |
|  | SANTI BAHN | 2 |  |  |  | H48 | 70.0 | 38.9 | **54.4** |
|  | SATHWA | 2 |  |  |  | H18 | 90.0 | 42.9 | **66.4** |
|  | SAVITRI | 2 |  | H2 | H6 | H17 | 95.0 | 71.4 | **83.2** |
|  | SINGHARA | 2 |  | H1 | H6 | H43 | 85.0 | 100.0 | **92.5** |
|  | SIPULUT PANDAN | 1 |  |  |  | H12 | 80.0 | 100.0 | **90.0** |
|  | SITA MATA | 2 |  | H8 |  | H63 | 80.0 | 81.8 | **80.9** |
|  | SOSSOKA | 1 |  | H5 | H4 | H8 | 78.6 | 70.6 | **74.6** |
|  | SUGAPANKH | 2 |  | H1 | H6 | H41 | 83.3 | 95.0 | **89.2** |
|  | SURAHA | 3 |  | H8 | H6 | H43 | 84.2 | 85.7 | **85.0** |
|  | SWARNA | 2 |  |  |  | H37 | 89.5 | 94.7 | **92.1** |
|  | SWARNA-SUB1 | 2 |  | H8 | H9 | H1 | 85.7 | 80.0 | **82.9** |
|  | TAGBA | 2 |  |  |  | H50 | 80.0 | 95.0 | **87.5** |
|  | TAH TONE | 1 |  |  |  | H50 | 85.0 | 64.7 | **74.9** |
|  | TATAN | 1 |  |  | H4 | H47 | 90.0 | 65.0 | **77.5** |
|  | TETEP | 2 |  | H3 | H6 | H41 | 94.7 | 76.2 | **85.5** |
|  | THUNMAR HAMARA | 2 |  |  |  | H62 | 75.0 | 30.0 | **52.5** |
|  | TIEBIMAH | 1 |  |  |  | H19 | 80.0 | 45.0 | **62.5** |
|  | TSAO WAN CHING | 2 |  |  |  | H50 | 52.9 | 86.7 | **69.8** |
|  | TULASI | 1 |  |  |  | H37 |  |  |  |
|  | TUNDAHIYA | 3 |  | H8 | H8 | H11 | 5.0 | 30.0 | **17.5** |
|  | USA BATAPOLA AL | 2 |  | H8 | H7 | H1 | 55.6 | 55.0 | **55.3** |
|  | VADAI | 1 |  | H2 | H6 | H31 | 35.0 | 77.8 | **56.4** |
|  | VAN | 1 |  |  |  | H1 | 10.0 | 14.3 | **12.1** |
|  | VANAN VELLAI | 1 |  |  | H6 | H17 | 60.0 | 70.6 | **65.3** |
|  | WAHNG | 2 |  |  |  | H50 | 60.0 | 30.0 | **45.0** |
|  | YEIKOYON | 1 |  |  |  | H39 | 52.6 | 40.0 | **46.3** |
|  | ZOBGUI | 2 |  | H8 | H9 | H1 | 60.0 | 63.2 | **61.6** |

**Table S17:** AEX (Sub1A1 allele-specific) marker amplification summary. Haplotypes H1 and H2 with a C at base pair position 556 code for Proline_186_, whereas haplotypes H3-H9 with T at the base pair position 556 code for Serine_186_ for the functional *Sub1A1* allele

| **Sub1A Allele** | **H1** | **H2** | **H3** | **H4** | **H5** | **H6** | **H7** | **H8** | **Total** | **Null or Unknown** |
| --- | --- | --- | --- | --- | --- | --- | --- | --- | --- | --- |
| **Allele frequency** | 3 | 28 | 9 | 1 | 4 | 1 | 1 | 49 | 96 | 83 |
| **Expected amplification** | 0 | 0 | 9 | 1 | 4 | 1 | 1 | 49 | 65 | 0 or  unknown |
| **Observed amplification** | 0 | 0 | 5 | 0 | 2 | 1 | 0 | 33 | 41 | 9 |
